# Coronal mass ejections and their sheath regions in interplanetary space

**DOI:** 10.1007/s41116-017-0009-6

**Published:** 2017-11-24

**Authors:** Emilia Kilpua, Hannu E. J. Koskinen, Tuija I. Pulkkinen

**Affiliations:** 1 Department of Physics, University of Helsinki, Helsinki, Finland; 2 Finnish Meteorological Institute, Espoo, Finland; 3 Department of Electronics and Nanoengineering, Aalto University, Espoo, Finland

**Keywords:** Coronal mass ejections, Solar wind, Space weather, Interplanetary shocks, Magnetic clouds

## Abstract

Interplanetary coronal mass ejections (ICMEs) are large-scale heliospheric transients that originate from the Sun. When an ICME is sufficiently faster than the preceding solar wind, a shock wave develops ahead of the ICME. The turbulent region between the shock and the ICME is called the sheath region. ICMEs and their sheaths and shocks are all interesting structures from the fundamental plasma physics viewpoint. They are also key drivers of space weather disturbances in the heliosphere and planetary environments. ICME-driven shock waves can accelerate charged particles to high energies. Sheaths and ICMEs drive practically all intense geospace storms at the Earth, and they can also affect dramatically the planetary radiation environments and atmospheres. This review focuses on the current understanding of observational signatures and properties of ICMEs and the associated sheath regions based on five decades of studies. In addition, we discuss modelling of ICMEs and many fundamental outstanding questions on their origin, evolution and effects, largely due to the limitations of single spacecraft observations of these macro-scale structures. We also present current understanding of space weather consequences of these large-scale solar wind structures, including effects at the other Solar System planets and exoplanets. We specially emphasize the different origin, properties and consequences of the sheaths and ICMEs.

## Introduction

Interplanetary coronal mass ejections (ICMEs) are macro-scale structures that are fundamentally important in shaping the heliospheric plasma and magnetic field, and driving space weather disturbances. They originate from gigantic magnetized plasma clouds, coronal mass ejections (CMEs^1^), which are launched from the Sun at a quasi-regular basis. These eruptions were first revealed with space-based optical coronagraphs in the 1970s (Tousey 1973; Hildner 1977) and defined as distinct white-light features propagating through the coronagraph’s field-of-view in time-scales from a few minutes to hours (e.g., Munro et al. 1979; Hundhausen et al. 1984).

**Fig. 1:**
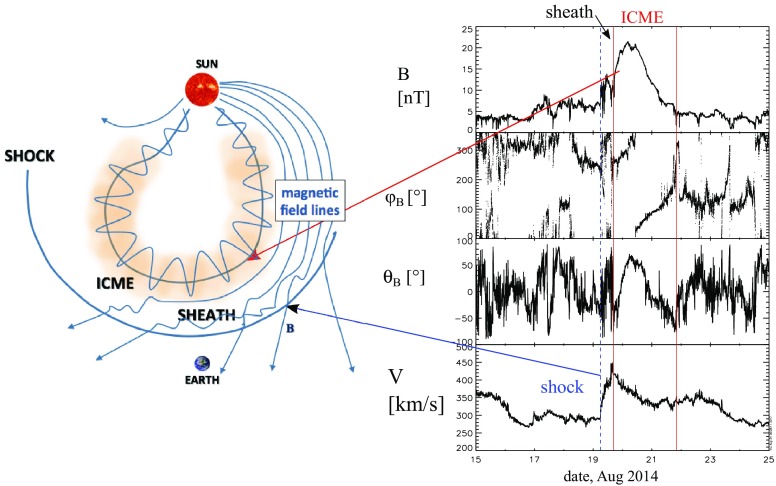
(Left) A schematic of an ICME showing also the leading fast forward shock (arc), and the sheath region. The ICME shown here is depicted to have a flux rope structure, not always detected in-situ. (Right) Solar wind observations during an ICME from the ACE spacecraft located at the Lagrangian point L1. The panels show from top to bottom: the magnetic field magnitude, the longitude and latitude angles of the magnetic field in the Geocentric Solar Magnetospheric (GSM) coordinate system, and the solar wind speed. The blue dashed line marks the shock and the ICME is bounded by the pair of red lines

The existence of interplanetary plasma clouds was suggested already before the space era and before discovery of CMEs. These speculations stemmed from the attempts to explain geomagnetic disturbances (e.g., Lindeman 1911; Chapman and Ferraro 1929; Bartels 1932) and so-called Forbush decreases in cosmic ray intensities (Forbush 1937; Morrison 1956; Cocconi et al. 1958; Piddington 1958). The first ICME observations emerged in the 1970s suggesting loop- or bubble-like structures behind interplanetary shocks (e.g., Hirshberg et al. 1970; Gosling et al. 1973; Palmer et al. 1978). For a more detailed historical review on ICMEs, and their role in understanding solar—terrestrial relationships, we guide the reader to Gopalswamy (2016). Since the discovery of ICMEs an extensive fleet of spacecraft and instrumentation has monitored the solar wind and its transient structures. In particular, from the mid-1990s the spacecraft near the Lagrangian point L1 (e.g., Wind, ACE, SOHO and DSCOVR) have provided continuous observations of the near-Earth solar wind.

Figure 1 shows a schematic picture and solar wind measurements during an ICME event. At the orbit of the Earth, the passage of an ICME past the observing spacecraft takes approximately one day, corresponding to a spatial structure of nearly one-third of the astronomical unit (AU). Signatures of ICMEs vary greatly, but on average, they are distinguished from the ambient solar wind by specific plasma, compositional and magnetic field signatures (e.g., Zurbuchen and Richardson 2006; Wimmer-Schweingruber et al. 2006). The ICME in Fig. 1 is clearly identified from the ambient solar wind by the enhanced magnetic field, which rotates in direction. As we will discuss later in this review, ICMEs with these specific signatures can be described in terms of a magnetic flux rope, i.e., a flux tube with helical magnetic field lines winding about the axis.

The sudden simultaneous increase of the magnetic field and speed in the data plot in Fig. 1 marks a fast mode shock wave, while the sheath extends from the shock to the ICME leading edge. ICME-driven fast shocks are particularly important structures in the collisionless solar wind plasma and effective accelerators of charged particles. The sheath regions in turn provide a unique natural plasma laboratory to study many important plasma properties such as turbulence and magnetic reconnection, and together with ICMEs they drive space weather disturbances. In particular, sheaths and ICMEs are the only interplanetary structures that can cause extreme geomagnetic storms.

In this article we give a review of ICMEs and their sheath regions, mostly based on observations, and we discuss their role in driving space weather disturbances. For information on CME properties and signatures in remote-sensing observations and their modelling we will guide the reader to, e.g., Living Reviews articles by Chen (2011), Webb and Howard (2012), and Schwenn (2006). We start by briefly describing signatures and observational properties of ICMEs, and their connection to CMEs (Sect. 2) followed by different modelling approaches (Sect. 3). In Sect. 4 we describe signatures and properties of sheath regions. Section 5 is devoted to the heliospheric and solar cycle evolution of ICMEs. Finally, Sect. 6 discusses space weather response of ICMEs and their sheaths at the Earth covering geomagnetic storms, solar energetic particles, radiation belts and the effects on planetary atmospheres and exoplanets.

## ICME signatures and properties

We start this section by reviewing general ICME signatures in the solar wind and move on to discuss the subset of ICMEs that contain magnetic flux ropes and the problem of defining boundaries of these structures. We continue by discussing average ICME properties, (e.g., average magnetic field magnitude, speed, and duration) near the Earth’s orbit and then give an overview of the current understanding of the large-scale morphology of ICMEs based on multi-spacecraft in-situ observations. We conclude this section by discussing briefly the connection between ICMEs and CMEs.

### General ICME signatures

**Fig. 2:**
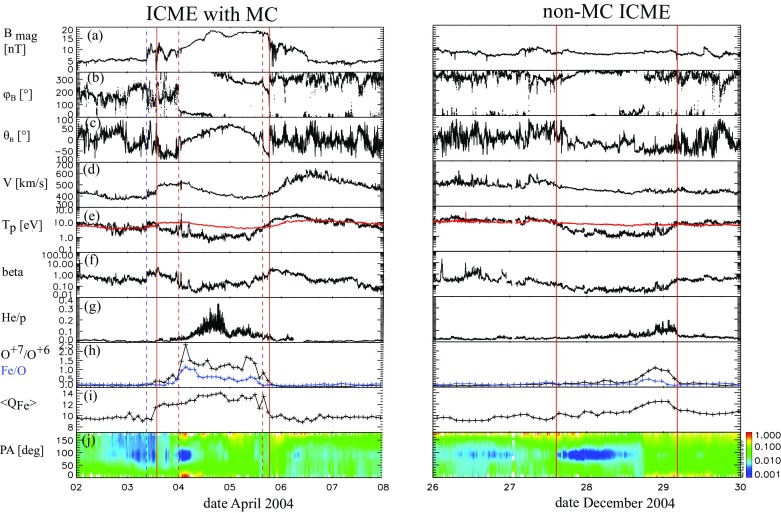
(Left) An ICME with a magnetic cloud (MC) structure and (right) a non-magnetic cloud ICME. The panels show from top to bottom: **a** magnetic field magnitude, the *b* longitude and *c* latitude angles of the magnetic field in GSM coordinates, $\phi _B=90^{\circ }$ ($\phi _B=270^{\circ }$) is defined eastward (westward), and $\theta _B=+90^{\circ }$ ($\theta _B=-90^{\circ }$) is defined northward (southward), solar wind *d* speed, *e* proton temperature (black: measured temperature, red: expected temperature, see Sect. 2.1), *f* plasma beta, *g* helium to proton ratio, *h* O$^{+7}/$O$^{+6}$ ratio (black) and Fe/O ratio (red), *i* iron average charge state, and *j* pitch angle spectrogram of 272-eV electrons. Pitch angle $0^{\circ }$ ($180^{\circ }$) refers to the particles that stream parallel (anti-parallel) to the magnetic field. A pair of red solid lines bound the ICME interval and the dashed blue line shows a shock. The red dashed lines bound the interval with MC signatures. The times are from the Richardson and Cane ICME list. The measurements are from the ACE spacecraft. Data have been obtained through the ACE Data Center (http://www.srl.caltech.edu/ACE/ASC/)

Figure 2 presents two distinctly different large-scale solar wind transients that both are classified as ICMEs. The solid red lines mark the start and end times of these ICMEs as defined in the Richardson and Cane ICME list^2^ (see also Richardson and Cane 1993, 2010). The ICME on the left drove a shock wave (dashed blue line), featured by abrupt and simultaneous increases of the magnetic field magnitude, solar wind speed and temperature.

The region limited by a pair of red dashed lines on the left-hand side of Fig. 2 features strong and smooth magnetic field and coherent rotation of the magnetic field components. These are the key characteristics of a magnetic cloud (MC), first defined by Burlaga et al. (1981) and Klein and Burlaga (1982) as a solar wind structure with (1) enhanced magnetic field when compared with the surroundings ($> 10$ nT), (2) smooth rotation of the magnetic field direction over a large angle in about one day, and (3) depressed proton temperature and plasma beta (i.e., the ratio of the plasma to magnetic pressure).^3^ The ICME on the right in turn lacks both the enhanced magnetic field and smooth rotation of the field direction throughout the whole interval. The field variability is, however, slightly depressed when compared to the surroundings.

During both our example ICMEs solar wind speed (panel 2d) decreases from the front to the rear edge. This is a common ICME signature indicating that these structures expand as they travel past the observing spacecraft (e.g., Klein and Burlaga 1982). The expansion leads to a low proton temperature. The regions of low proton temperature following some interplanetary shocks were first noted by Gosling et al. (1973) and this is nowadays among the most commonly used ICME proxies. Inspired by this observation, Richardson and Cane (1995) used a solar wind speed-temperature relationship obtained from OMNI data by Lopez (1987) to calculate an “expected temperature” ($T_\mathrm{exp}$) from the observed solar wind speed. They then demonstrated that low temperature intervals ($T_p < 0.5\,T_\mathrm{exp}$) that were not associated with the heliospheric plasma sheet were predominantly associated with regions where other ICME signatures were present. The red horizontal line in Fig. 2e shows the expected temperature, which is clearly higher than the temperature that was measured within both our example ICMEs, including the non-MC parts of the ICME on the left-hand side.

Figure 2g shows the helium to proton ratio in the solar wind. Enhanced abundances of helium relative to protons following shock waves were among the earliest identified ICME signatures, although at the time, they were identified with a solar flare source (Hirshberg et al. 1970, 1972; Borrini et al. 1982). While some individual ICMEs indeed show clear enhancements of the alpha to proton ratio (larger than 0.08–0.1), statistical studies have shown that on average, this ratio in ICMEs is not that distinct from values found in the slow solar wind (e.g., Lynch et al. 2003; Richardson and Cane 2004; Rodriguez et al. 2016). The next panels gives the $\hbox {O}^{+7}/\hbox {O}^{+6}$ and Fe/O ratios, and the iron average charge state. The solar wind ion charge states “freeze-in” beyond a few solar radii from the Sun where the propagation time of the solar wind is short compared to the recombination–ionisation time-scale (e.g., Zhao et al. 2009). ICMEs show increased abundance of high charge states. For example, the $\hbox {O}^{+7}/\hbox {O}^{+6}$ ratio $> 1.0$ (e.g., Henke et al. 2001), and average iron charge states $> 12$ (e.g., Lepri et al. 2001) are good signatures of the ICME-plasma. On the other hand, the elemental ratios depend, in a complex way, on chromospheric temperatures, the magnetic field configuration at the origin of the plasma, and the plasma confinement time before the release (e.g., Laming (2015), and references therein). In ICMEs, increased amounts of high charge states of elements such as Fe, Ne, Si, Mg are often observed suggesting extended confinement times (e.g., Lepri et al. 2001; Zurbuchen et al. 2016).

Charge state and compositional anomalies are most common in fast ICMEs and in MCs (e.g., Burlaga et al. 2001; Henke et al. 2001; Richardson and Cane 2004; Aguilar-Rodriguez et al. 2006; Rodriguez et al. 2016). The helium to proton, O$^{+7} /$ O$^{+6}$ and Fe/O ratios are indeed considerably higher within the MC than non-MC parts for the event shown on the left in Fig. 2. The average iron charge states are however elevated throughout the whole ICME interval. In turn, the ICME on the right that did not contain MC at all shows enhanced values only towards its end.

The bottom panel of Fig. 2 shows the pitch angle spectrogram of suprathermal ($\gtrsim 100$ eV, here the 272-eV channel is chosen) electrons that carry the solar heat flux and give information of the magnetic connectivity. If the magnetic field lines are attached to the Sun only from one end, a uni-directional electron flow is observed, while a closed configuration gives rise to a bi-directional electron flow both parallel and antiparallel to the magnetic field (e.g., Gosling et al. 1987). The closed configuration can either represent a case where field lines are attached to the Sun at both ends (see Fig. 1), or a detached plasmoid. We will return to this question later in this review. The ICME on the left in Fig. 2 features bi-directional electron flow. Such counter-streaming signatures behind interplanetary shock waves were already noted by Bame et al. (1981), and shown to be present in a large sample of ICMEs by Gosling (1990). Many ICMEs, including our example events, present intermittent counter-streaming electrons, suggesting a mixture of open and closed magnetic field lines (e.g., Gosling 1990; Shodhan et al. 2000). Again, counter-streaming electrons are not a unique ICME signature. They are commonly observed also as reflections from interplanetary shocks or from the Earth’s bow shock (e.g., Steinberg et al. 2005).

**Fig. 3:**
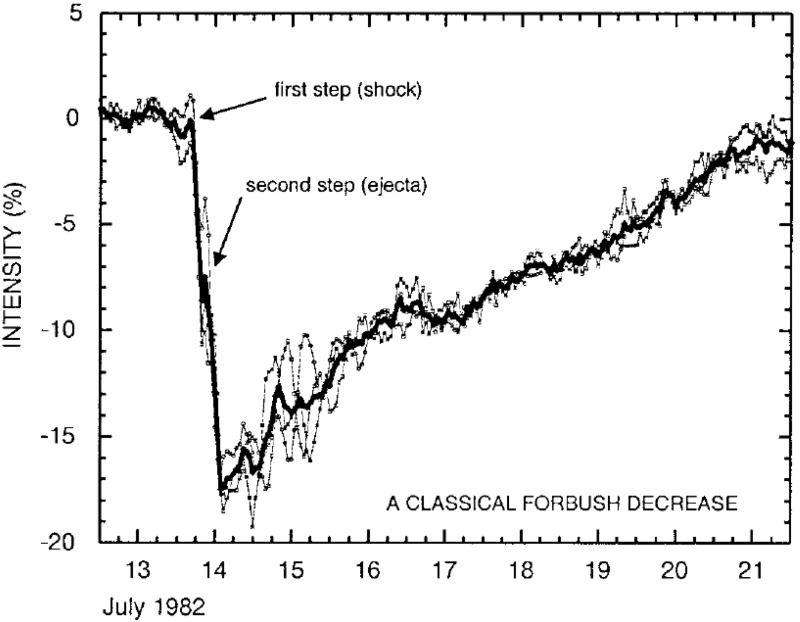
Forbush decrease associated with a shock-driving ICME. Image reproduced by permission from Cane (2000), copyright by Kluwer

Other useful indicators of ICMEs are Forbush decreases, i.e., transient reductions (up to 20–25%) in galactic cosmic ray intensities observed both on the ground by neutron monitors and by space instrumentation (for thorough reviews see e.g., Lockwood 1971; Cane 2000). For example, a statistical study by Richardson and Cane (2011) of over 300 ICMEs showed that 80% of them were associated with a Forbush decrease. Figure 3 shows a Forbush decrease where both shock/sheath and ICME participate to the decrease. Strong magnetic fields in CMEs prevent galactic cosmic rays from diffusing into them, as they propagate through interplanetary space, while in sheaths turbulence also plays a major role (e.g., Wibberenz et al. 1998; Cane 2000). As shown by Richardson and Cane (2011), minimum cosmic ray intensities occur typically within the ICME and MCs cause on average deeper Forbush decreases than non-MC ICMEs. Forbush decreases can be particularly important for identifying ICMEs when limited magnetic field and/or plasma data are available. They, however, are not unique to ICMEs and, as pointed out by Cane (2000), even at 1 AU Forbush decreases can be difficult to interpret. Jordan et al. (2011) also pointed out that small-scale magnetic structures in ICME sheaths can affect significantly the galactic cosmic ray profiles.

Despite the commonly agreed ICME signatures we have discussed above, the identification of the ICMEs is often ambiguous (e.g., Gosling 1997; Richardson and Cane 2010; Kilpua et al. 2013b). The key issue is, as also highlighted by our example events, that there is no specific signature that would always be present in an ICME and different signatures may come and go during the passages of a given ICME.

### Flux ropes in ICMEs

As we will show in Sect. 3, MCs can be described in the first approximation as cylindrically symmetric force-free flux ropes. The force-free assumption ($\mathbf{J}\times \mathbf{B}=0$, where where **B** is the magnetic field vector and **J** the electric current density) is reasonable considering that the low plasma beta is one of the key MC criteria and that the plasma pressure *p* has generally very modest variations within the MC (i.e., $\nabla p \approx 0$). Flux ropes have been in the centre of research as they often drive strong geospace storms (Sect. 6) and provide a link to erupting CME properties and source conditions at the Sun, e.g., magnetic flux and helicity (van Driel-Gesztelyi et al. 2003; Démoulin et al. 2016). The magnetic helicity describes how magnetic field lines are wound around each other and it is defined as an integral $\int dV \mathbf{A } \cdot \mathbf{B }$, where **A** is the magnetic vector potential (e.g., Berger and Field 1984).

**Fig. 4:**
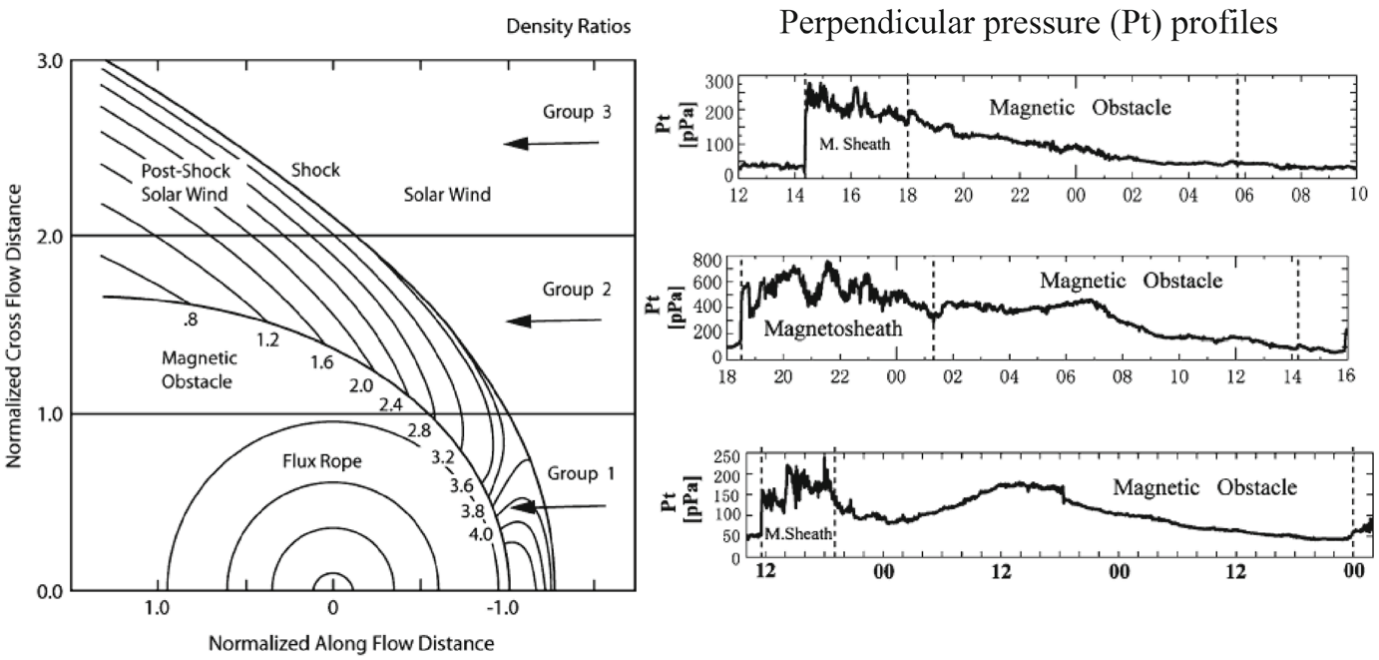
Different spacecraft paths (Group 1–Group 3) through an ICME and the corresponding perpendicular pressure ($P_t$) profiles. The different groups are described in the text. The contours in the figure show the density and the numbers give the density ratios between the solar wind and magnetopause values. In the right-hand part of the figure the first dashed line shows the shock and the following two dashed lines bound the ICME interval. Images reproduced by permission from Jian et al. (2006), copyright by Springer

However, only about one-third of ICMEs at 1 AU show clear MC signatures (e.g., Gosling 1990; Bothmer and Schwenn 1996; Cane and Richardson 2003; Huttunen et al. 2005; Wu and Lepping 2011). It is believed that a significantly larger fraction of ICMEs contains flux ropes, but they are not always detected because the spacecraft crosses the flux rope too far from the centre. This was first confirmed using multispaceraft observations by Cane et al. (1997) who analyzed ICMEs detected both by Helios 1 and 2, and later with STEREO and L1 observations (e.g., Kilpua et al. 2011). Jian et al. (2006) in turn demonstrated this by examining characteristic profiles of the total pressure perpendicular to the magnetic field ($P_t$; for further discussion see Russell et al. 2005). Different paths through the MC and corresponding $P_t$ profiles are shown in Fig. 4. Group 1 events where $P_t$ attains its maximum at the centre are cases where the flux rope is crossed centrally. In Group 2, the spacecraft crosses the ICME at an intermediate distance from the centre. The $P_t$ profile has first a plateau, followed by a slow decline. Finally, in Group 3, the $P_t$ profile shows a rapid increase (shock/sheath) followed by a slow decline. These are glancing encounters where whole or most of the ICME is missed.

An ICME can also deform and its magnetic flux can erode significantly during its interplanetary propagation. In particular near solar maximum multiple successive ICMEs may merge together. As a consequence, individual flux rope characteristics are lost (see Sect. 5.4). One possibility is also that ICMEs with and without MCs have different birth mechanisms at the Sun. However, this does not seem likely as flux ropes are invoked as integral part of the CME eruption, being present in the majority, if not in all CMEs (e.g., Vourlidas et al. 2013)

**Fig. 5:**
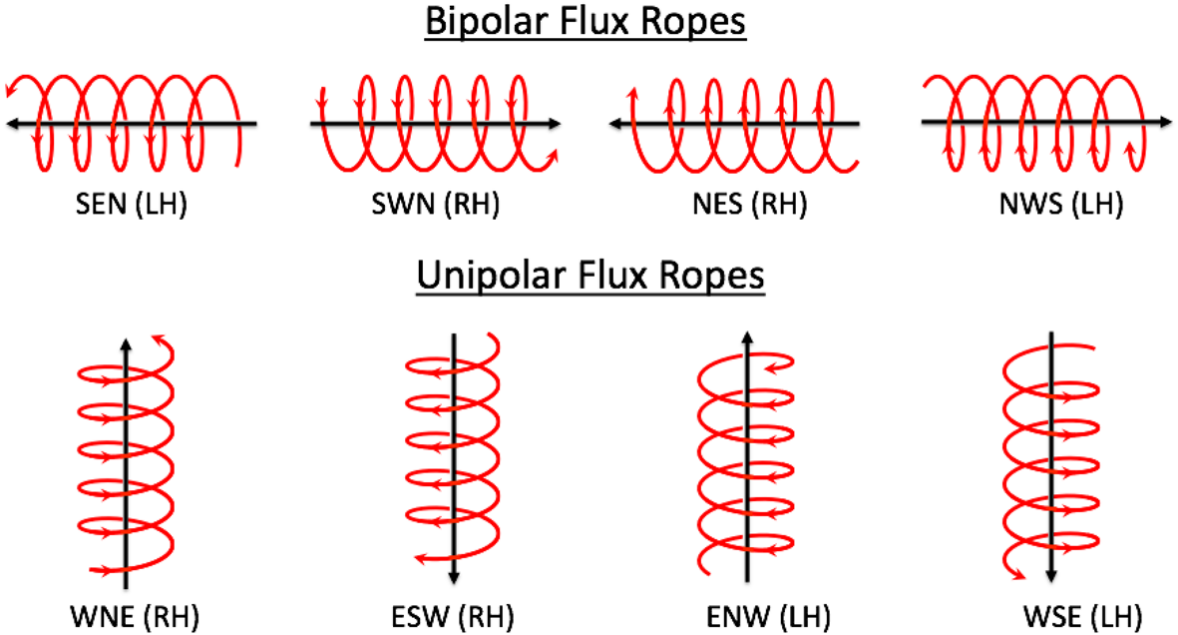
Flux rope categories for Top) bipolar (low-inclination) and Bottom) unipolar (highly-inclined) magnetic clouds. The letters above each flux rope defines the direction of the magnetic field at the leading edge of the MC, at its centre, and at its trailing edge (E $=$ East, W $=$ West, N $=$ North, S $=$ South), assuming that the spacecraft moves into the page. The sign of magnetic helicity is either left-handed/negative (LH; clockwise rotation) or right-handed/positive (RH; counter-clockwise rotation). Image courtesy of Erika Palmerio

The way the magnetic field vectors rotate within a given MC is determined by the orientation of the cloud’s axis with respect to the ecliptic plane, the direction of the axial magnetic field and the sign of magnetic helicity. The characteristic field patterns define eight flux rope types shown in Fig. 5 (Mulligan et al. 1998; Bothmer and Schwenn 1998). The upper part of the figure shows flux ropes with the axis lying close to the ecliptic plane. The interplanetary magnetic field (IMF) north-south component ($B_Z$) changes its sign from the leading to the trailing edge, and therefore, such MCs are called bipolar. In contrast, unipolar MCs, in the lower part of the figure, are highly inclined to the ecliptic plane and $B_Z$ maintains its sign. The MC in Fig. 2 is a unipolar ENW-type flux rope. The latitude angle of the magnetic field stays positive during most of the MC, i.e., $B_Z$ remains roughly northward (N), and the field rotates from the East (E; see definitions of the directions from the Fig. 2 caption) at the leading edge of the cloud to the West (W) at the trailing edge. The counter-clockwise rotation of the magnetic field direction is referred as right-handed (positive) magnetic helicity, while clockwise rotation is defined left-handed (negative) helicity.

### Identifying ICME boundaries

Identification of ICME boundaries is often an ambiguous and subjective. It is a well known issue that the boundary times in various catalogues may differ considerably (see discussions, e.g., in Richardson and Cane 2010; Kilpua et al. 2013b).

**Fig. 6:**
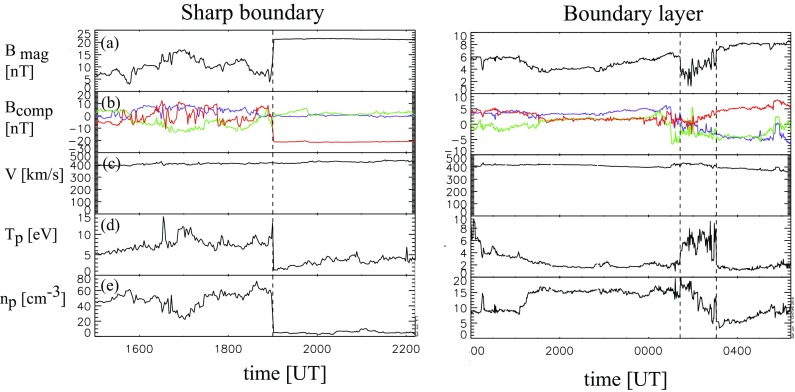
Examples of sheath–ICME boundaries: **a** magnetic field magnitude, **b** components of the magnetic field in GSE coordinates ($B_X$: purple, $B_Y$: green, $B_Z$: red), solar wind **c** speed, **d** proton temperature, and **e** density. In the left-hand panels the dashed vertical line indicates the sharp boundary and in the right-hand panels the pair of dashed lines marks a wider boundary layer. Both cases are discussed in Wei et al. (2003a)

An example of a particularly clear and sharp sheath-ICME boundary is shown in the left-hand panels of Fig. 6. The transition from the turbulent sheath to the smooth MC is abrupt and coincides with distinct drops in the plasma density and temperature. In most cases the boundary is far from that clear. A statistical study of 80 MCs by Wei et al. (2003a) showed that the majority of them were bounded by boundary layers with average passage durations of 1.7 and 3.1 h at the front and rear edges, respectively. The right-hand side of Fig. 6 shows an example of such a boundary layer at the front edge of the MC, bounded by a pair of dashed lines. The boundary layer has increased densities and temperatures, decrease in the magnetic field, and abrupt changes in the magnetic field direction. As noted by Wei et al. (2003a) these are common features of boundary layers and can be interpreted as signatures of magnetic reconnection.

Wei et al. (2003b) studied fluctuation characteristics in 23 MC boundary layers and found that the distribution functions of field fluctuations were distinctly different in the boundary layer, MC and the ambient solar wind. The authors reported Alfvénic fluctuations within the boundary layers. Based on these distinct signatures Wei et al. (2003b) concluded that boundary layers are likely distinct structures from the sheath and the MC, resulting either from the interaction between the cloud and the ambient solar wind, or being relics of the CME release process (see also Farrugia et al. 2001; Kilpua et al. 2013b).

**Fig. 7:**
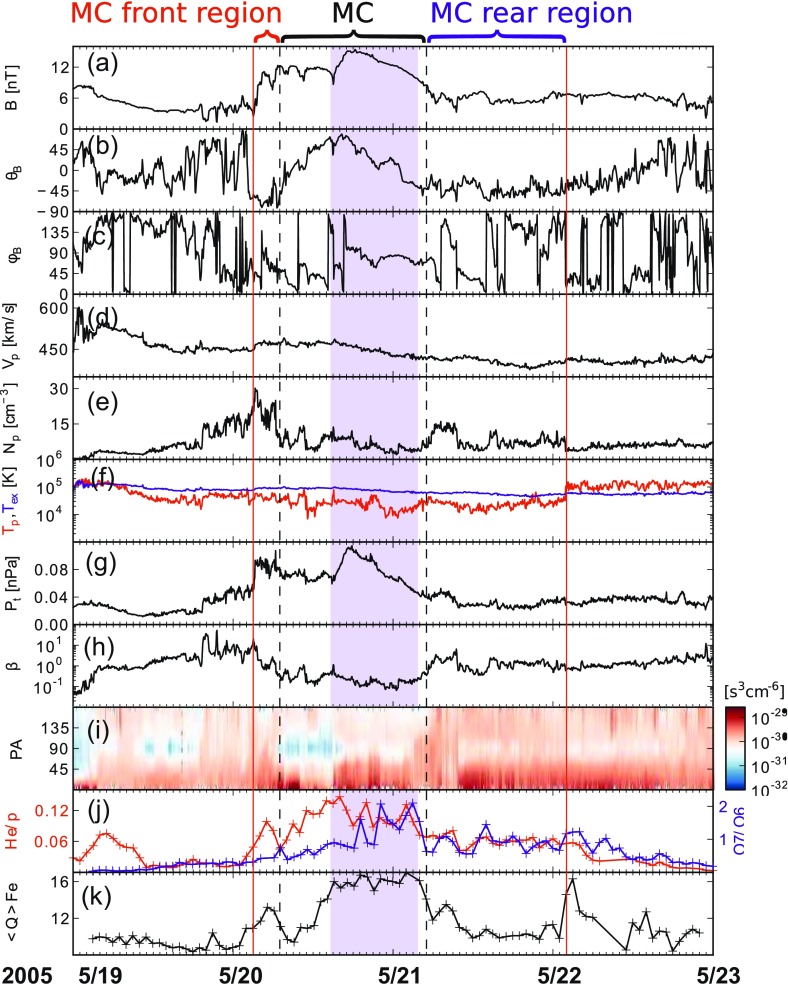
Different regions of the ICME. The panels show the magnetic field **a** magnitude, **b** latitude, and **c** longitude, **d** solar wind speed, **e** density **f** temperature (black: measured, red: expected temperature), **g** total pressure perpendicular to the magnetic field, **h** plasma beta, **i** pitch angle spectrogram of 272-eV electrons, **j** red: helium to proton ratio, black: O$^{+7}$/O$^{+6}$ ratio, and **k** average iron charge state. The data are from the ACE spacecraft. Images reproduced by permission from Kilpua et al. (2013b), copyright by the authors

Our example event in Sect. 2.1 showed that an MC can be embedded in a more extended ICME interval. According to Kilpua et al. (2013b) this is a common phenomenon. The authors analyzed the events in the Richardson and Cane list and found that only in 30% of the cases the MC and ICME boundaries coincide both at the leading and trailing edge within 2 h. Another example is presented in Fig. 7 where the MC is bounded by a pair of black dashed lines and the ICME by the red solid lines. Some ICME-related solar wind signatures continue to be observable almost one day after the encounter of the MC rear boundary, and they are also present a few hours before the start of the MC. The gray area marks the flux rope interval captured by the Grad–Shafranov reconstruction (see Sect. 3.2) and it features the most distinct charge state and compositional anomalies and is bounded by boundary layers discussed above. Richardson and Cane (2010) also noted that in a considerable fraction of cases (22%) compositional signatures continue even several hours past the ICME boundaries that were defined based on the plasma and magnetic field data.

### Average ICME/MC properties

Some key ICME properties are summarized in Table 1. We have calculated these values using the 1-h OMNI database and the ICMEs are extracted from the online catalogues compiled by Lan Jian^4^ and by Richardson and Cane.^5^ Information on the ICME identification criteria in these catalogues is given in their respective webpages (see also Jian et al. 2006; Richardson and Cane 2010). For the Lan Jian catalogue we give the results separately for the centrally crossed events (Group 1, Fig. 4) and the events that are crossed at the intermediate distance from the centre (Group 2), defined using the $P_t$ profiles. For the Richardson and Cane catalogue we give the results separately for MCs and non-MC ICMEs.

**Table 1:** Average ICME properties based on the Lan Jian (1995–2009) and Richardson and Cane (1996–2016) online catalogues. The last row shows the values for all 1-h OMNI data periods for the interval 1995–2016 (ICME intervals not removed). The columns show the number of events (N), averages of the maximum magnetic field ($\langle B_{\max } \rangle$), mean magnetic field ($\langle B_\mathrm{ave} \rangle$), maximum speed ($\langle V_{\max } \rangle$), mean speed ($\langle V_\mathrm{ave} \rangle$), and duration ($\langle dT \rangle$). The errors give the standard deviations. The last two columns give the average of the speed gradient from the trailing to the leading edge for expanding events (i.e., negative gradient), and the percentage of expanding events

Source	N	$\langle B_{\max } \rangle$ [nT]	$\langle B_\mathrm{ave} \rangle$ [nT]	$\langle V_{\max } \rangle$ [km s$^{-1}$]	$\langle V_\mathrm{ave} \rangle$ [km s$^{-1}$]	$\langle dT \rangle$ [h]	$\langle \varDelta V\rangle$ [km s$^{-1}$]	$\varDelta V< 0$ [%]
Group 1	91	$16.6\pm 8.7$	$11.4\pm 4.7$	$504\pm 144$	$448\pm 107$	$29.9\pm 13.8$	$-75.2\pm 13.2$	88
Group 2	69	$13.6\pm 5.2$	$10.1\pm 3.5$	$531\pm 152$	$478\pm 123$	$25.0\pm 11.8$	$-67.9\pm 11.3$	88
MC	170	$17.2\pm 8.2$	$12.9\pm 4.1$	$502\pm 145$	$452\pm 102$	$20.8\pm 11.2$	$-57.8\pm 11.5$	85
Non-MC	147	$11.3\pm 5.5$	$7.5\pm 3.1$	$540\pm 144$	$475\pm 109$	$28.0\pm 16.4$	$-93.5\pm 18.2$	76
All SW			$5.2\pm 3.0$		$429\pm 102$			

Table 1 shows that, as expected, MCs have on average stronger magnetic fields than non-MCs. In addition, Group 1 events have, on average, stronger magnetic fields than Group 2 events. This result is consistent with the assumption that non-MC ICMEs are generally encounters through the edge/leg of the flux rope loop. However, considering the large standard deviations, there is no significant difference either in average or maximum speeds between MCs and non-MC events and between Group 1 and Group 2 events. MCs are considerably shorter in duration than non-MC events. The results discussed above are consistent with the previous studies, e.g., with Richardson and Cane (2010) and Wu and Lepping (2011), who analyzed Solar Cycle 23 events, and Bothmer and Schwenn (1998) and Forsyth et al. (2006) who derived radial dependencies of various ICME parameters using Helios 1 and 2 data between 0.3–1 AU.

We also note that ICMEs have on average much stronger magnetic fields than the average IMF. In turn, ICMEs have their average speeds similar to the average solar wind speed, implying that near the Earth orbit ICMEs are not propagating much faster through the solar wind. This result is consistent with works, e.g., by Lindsay et al. (1999) and Gopalswamy et al. (2000) suggesting that CMEs decelerate/accelerate to the speed of the ambient solar wind. Their average maximum speeds that typically occur at the leading edge, however, are clearly above the overall solar wind average.

As we mentioned in Sect. 2.1, declining speed profile is a common ICME signature. Table 1 confirms that a clear majority (about 80%) of ICMEs expand at 1 AU, i.e., they have a negative rear-to-front speed gradient ($\varDelta V$). Group 1 and Group 2 events have exactly the same percentage of expanding events, but MCs expand more often than the non-MC ICMEs. However, the average expansion speed is larger for non-MCs. Typical speeds at which ICMEs expand are about one-third of their propagation speeds and of the order of half of the local Alfvén speed in the solar wind frame (e.g., Klein and Burlaga 1982; Owens et al. 2005; Gulisano et al. 2010). The rear-to-front speed gradient does not express how fast a fluid element expands. Gulisano et al. (2010) found that all magnetic clouds that were not significantly perturbed by the interactions with the ambient solar wind expand with almost at the same non-dimensional expansion rate, defined as

1 $$\begin{aligned} \psi = {\varDelta V_x \over \varDelta t} {D \over V_c^2} \, \end{aligned}$$

where $\varDelta t$ is the duration of the magnetic cloud, $V_x$ is the measured velocity component in the radial direction from the Sun, $V_c$ is the speed at the centre, and *D* is the radial distance from the Sun.

### Large-scale structure of ICMEs from observations

**Fig. 8:**
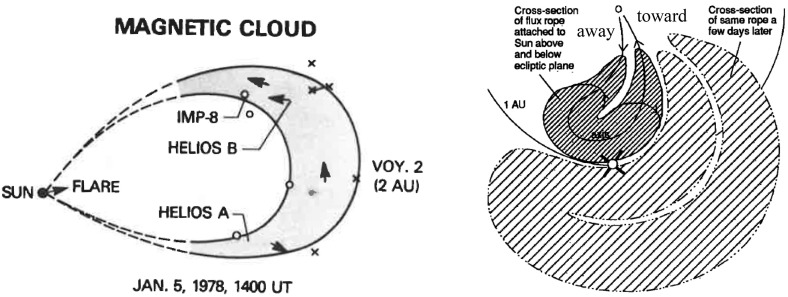
(Left) The global flux rope axis configuration as deduced from multi-spacecraft observations for a magnetic cloud observed on January 6–8, 1978. All these spacecraft were located close to the ecliptic plane at radial distances between 1–2 AU. (Right) Schematic of a flux rope loop that is distorted along the Parker spiral and carries a sector boundary crossing. Images reproduced by permission from [left] Burlaga et al. (1990); [right] from Crooker et al. (1998), copyright by AGU

The magnetic cloud found by Burlaga et al. (1981) (see Sect. 2.2) was detected by five longitudinally widely separated spacecraft (IMP-8, Helios A and B, Voyager 1 and 2; left part of Fig. 8). The later analysis of this event by Burlaga et al. (1990) showed that these observations could be interpreted in terms of a huge and curved flux rope loop extending back towards the Sun. Recently, Janvier et al. (2015) constructed the average global shape of the MC axis from a large number of single-spacecraft observations and the results were consistent with the above suggestion. The rapid access of solar energetic particles into MCs and frequent presence of bidirectional suprathermal heat fluxes in MCs then provides evidence that field lines within MCs remain attached to the Sun, often at both legs (e.g., Kahler and Reames 1991; Richardson 1997; Larson et al. 1997). It should be noted that flux ropes do not generally extend radially from the Sun, but are rather distorted along the Parker spiral bending back onto themselves (Crooker et al. 1998; Rees and Forsyth 2004) (right-hand part of Fig. 8).

Multi-spacecraft observations also constrain the extent of ICMEs. Depending on whether the observing spacecraft are separated primarily in longitude or in latitude and whether the flux rope is low- or highly-inclined, the observations allow either the transverse extent of the flux rope loop or its cross-section to be estimated. For example, observations from spacecraft separated in longitude can give an estimate for the extent of the flux rope loop for low inclination MCs and for the cross-section thickness for high-inclination MCs. The event studied by Burlaga et al. (1981, 1990) was a low-inclination MC and based on the separation between the spacecraft it is possible to conclude that the flux rope loop extended at least $30^{\circ }$ in longitude.

One of the main science objectives of the twin STEREO mission was to better constrain the global ICME morphology. Unfortunately, due to a long period of low solar activity following the launch of STEREO in October 2006 only a handful of events were observed in interplanetary space simultaneously by more than one spacecraft, including STEREO—near-Earth combinations. However, MESSENGER, Venus Express, STEREO and the near-Earth spacecraft have more recently provided several multi-spacecraft ICME encounters from widely separated vantage points (see e.g., the list in Good and Forsyth 2016). The Good and Forsyth (2016) study based on these missions show that only about one-third of the ICMEs were detected by two or more spacecraft when their separation was $30^{\circ }$ falling to 12% when the spacecraft were separated by $45-60^{\circ }$. These results are consistent with the Cane et al. (1997) analysis based on Helios 1 and 2 observations. Recently, Witasse et al. (2017) reported an ICME that extended more than $100^{\circ }$ in longitude. However, there are cases in which ICME signatures have been remarkably different even when the spacecraft were only a few degrees apart (e.g., Mulligan et al. 1999; Kilpua et al. 2011). Such observations were made for high-inclination MCs that occurred during solar minimum.

Both in-situ observations of ICMEs and simulations of CME propagation suggest that their cross-sections are highly oblate. This implies that ICMEs expand faster in the non-radial direction. By comparing typical sheath and MC thicknesses to the standoff distance of the planetary bow shocks, Russell and Mulligan (2002) concluded that near the Earth’s orbit the dimensions of the flux rope cross-sections in the direction perpendicular to the plane containing the flux rope axis are about four times their radial thicknesses. Direct observations suggest even larger aspect ratios: Liu et al. (2006b) and Mulligan et al. (1999) estimated typical aspect ratios not to be smaller than 6:1. Crooker and Intriligator (1996) reported an MC that was compressed by another ICME and for which the azimuthal width exceeded the radial width by at least a factor of 8. Some in-situ studies, however, suggest more circular cross-sections (e.g., Liu et al. 2008; Möstl et al. 2009; Kilpua et al. 2011), but these have used the Grad–Shafranov modelling (see details in Sect. 3.2) that is known to underestimate the elongation (e.g., Riley et al. 2004; Kilpua et al. 2011).

**Fig. 9:**
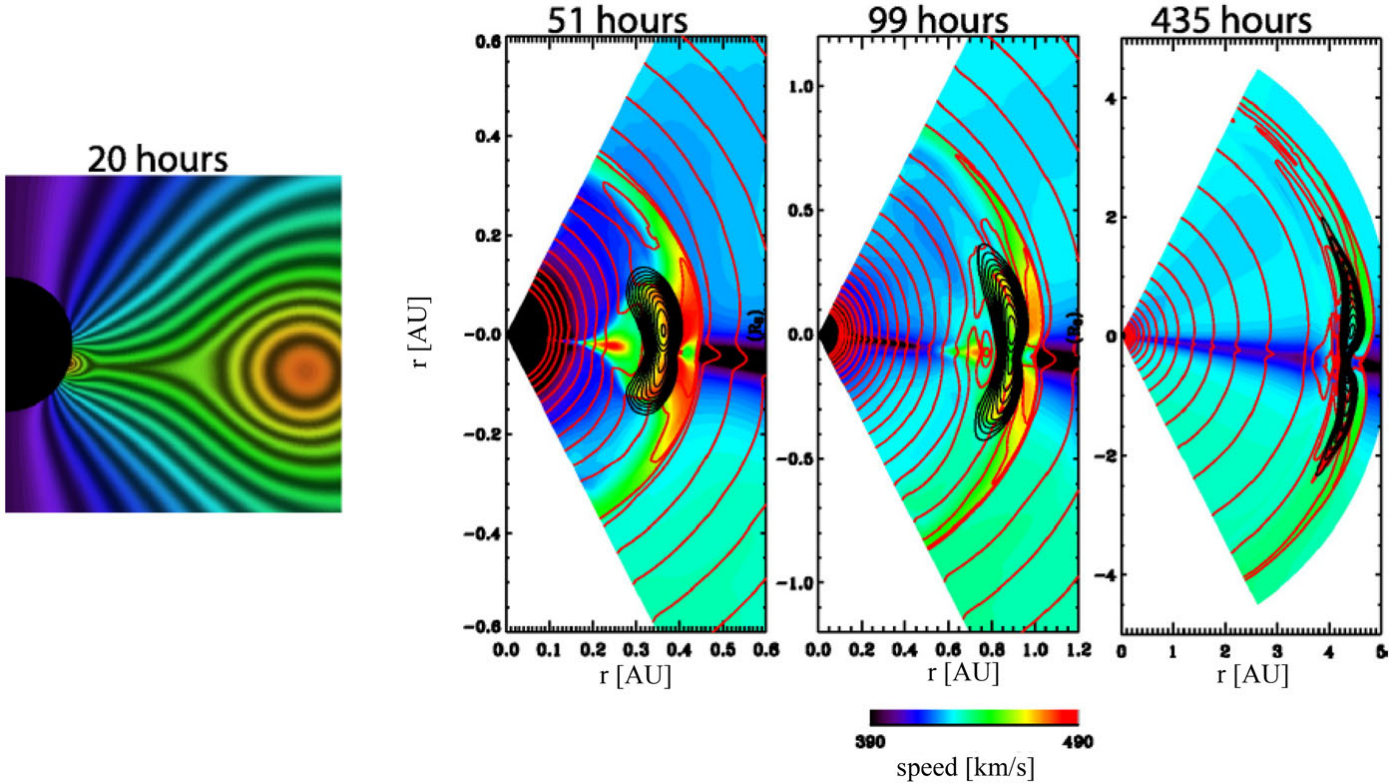
Snapshot of the CME evolution for an MHD simulation at four different times. The colour map shows the radial velocity, the black contours the magnetic flux, and the red contours the number density. Image reproduced by permission from Riley and Crooker (2004), copyright by AAS

Numerical MHD simulation results by Riley and Crooker (2004) shown in Fig. 9 illustrate how the shape of the CME elongates as it propagates from the Sun due to kinematic and dynamic effects. The cross-section at Earth orbit (1 AU) is clearly not circular, but significantly elongated as discussed above. Furthermore, the cross-section is not elliptical but convex-outward. Liu et al. (2006b) showed that due to the variations in latitudinal solar wind pattern from solar minimum to maximum ICME cross-sections tend to curve in the opposite ways: Near solar minimum ICME cross sections are bent concavely outward, while near solar maximum cross sections tend to bend convexly. This is because near solar maximum the solar wind speed is approximately the same over all latitudes whereas near minimum the speed increases significantly from the equator towards the poles (e.g., McComas et al. 2001). Simulations also emphasize that the properties of ICMEs can substantially vary at different sampling locations due to the varying solar wind density and speed structures (e.g., Odstrčil and Pizzo 1999a, b).

### Connection between ICMEs and CMEs

Several studies helped to establish the connection between CMEs observed remotely in the corona and ICMEs observed in-situ in interplanetary space. For example, Burlaga et al. (1982) showed, based on timing considerations, that an interplanetary plasma cloud detected by Helios-1 could be associated with a large CME previously detected by the coronagraph onboard the Solwind spacecraft. Early statistical connection was established, e.g., by Schwenn (1983), who connected 19 Helios ICMEs to Solwind CMEs. Wilson and Hildner (1984) also showed that six out of the nine interplanetary plasma clouds they investigated could be linked to type II radio bursts that indicate CME-driven shock waves in the corona. The striking connection between CMEs and interplanetary shocks was made by Sheeley et al. (1985). The authors demonstrated that 72% of the shocks identified during the period 1979–1982 by Helios 1 were associated with large, low-latitude CMEs detected by Solwind and for additional 26% of shocks there was a possible Solwind–CME association. Since these early studies, CME–ICME association has been detailed, in particular using observations by the LASCO coronagraph onboard the SOHO spacecraft and in-situ observations by the L1 spacecraft Wind and ACE (e.g., Webb et al. 2000; Schwenn et al. 2005, and references therein).

**Fig. 10:**
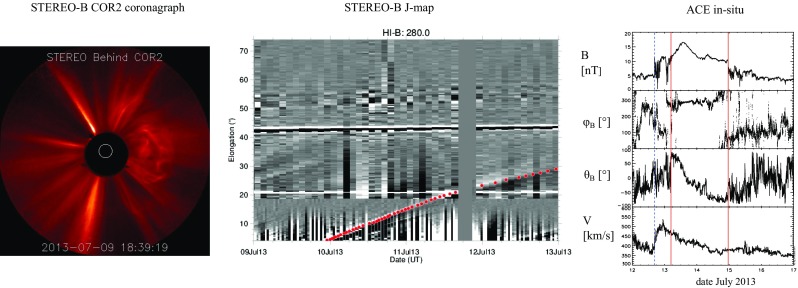
Example event from the HELCATS database. (Left) STEREO-B COR2 coronagraph image of an Earth-directed CME that erupted on July 9, 2013. (Middle) J-map that has been constructed using STEREO-B heliospheric imager observations. Red dots show the model fitted values and they indicate the path of the CME. (Right) ACE in-situ observations of the associated ICME. The panels are the same as in Fig. 1. The blue dashed line marks the shock and the ICME is bounded between the pair of red lines

Wide angle heliospheric imaging (e.g., SMEI/Coriolis and SECHHI/STEREO; Eyles et al. 2003; Harrison et al. 2005) allows some CMEs to be followed all the way from the Sun to in-situ detection. The European Union FP7-funded HELCATS project^6^ has built an extensive database of CMEs observed with STEREO heliospheric imagers (HI), including solar, coronagraph and in-situ associations. Figure 10 shows an event from the HELCATS database. On left is the CME as seen in the STEREO-B COR2 coronagraph image on July 9, 2013 18:39 UT. The middle panel shows the time-elongation plot, or J-map (for the details of the technique see, e.g., Davies et al. 2009) that has been constructed from running time difference of STEREO-B/HI observations. This CME could be tracked (the red dots) to its arrival to Earth on July 12. The right-hand panels show a shock on July 12 14:46 UT followed by a clear MC featuring enhanced magnetic field and smoothly rotating field direction starting around July 13 04:45 UT.

**Fig. 11:**
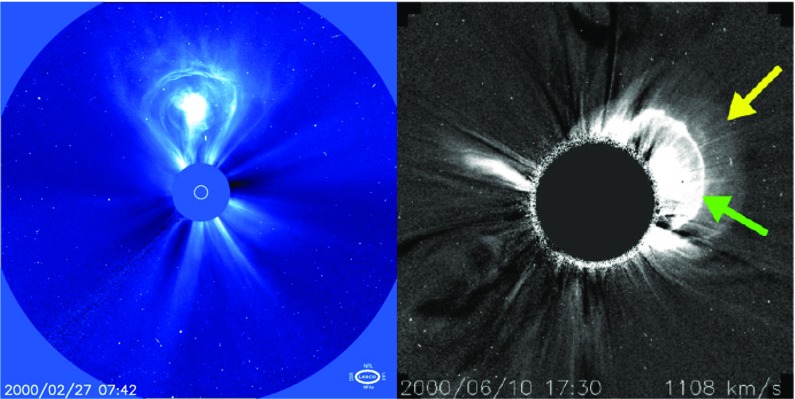
(Left) A CME with a classical three-part structure detected one February 27, 2000 in SOHO/LASCO C3 image (Image courtesy: NASA). (Right) An excess mass image illustrating two more signatures of a five-part CME on June 10, 2000 in SOHO/LASCO C2 image. The yellow arrow indicates the sharp edge (shock) and the green arrow the bright front. Image reproduced by permission from Vourlidas et al. (2013), copyright by Springer

Both white-light coronagraphs and heliospheric imagers record sunlight that has been scattered by free electrons in the corona and in heliosphere. The images based on these techniques give 2-dimensional electron density distributions that are integrated along the line-of-sight. The left-hand panel in Fig. 11 shows a classical three-part (“light-bulb”) CME morphology in a coronagraph image. The low density “dark cavity” corresponds to the flux rope, while the brightest features are a dense prominence core and a frontal rim of coronal loops that pile up at the flux rope leading edge (Illing and Hundhausen 1983; Dere et al. 1999; Gibson and Low 2000). The excess mass image in the right-hand panel of Fig. 11 shows two additional features: a faint and wide front that represents a temporary compression as the CME-driven wave/shock moves through the corona and a sharp edge that corresponds to the wave/shock itself (Vourlidas et al. 2013). We also note that in the J-map shown in Fig. 10 the bright track correspond the compressed sheath region, not the lower density flux rope (e.g., Lugaz et al. 2005; Rouillard 2011).

Although physically similar features can be distinguished from remote-sensing and in-situ observations (shock, sheath, flux rope) the detailed correspondence between CMEs and ICMEs is not at all clear yet (e.g., Crooker 2005; Crooker and Horbury 2006; Rouillard 2011; Kilpua et al. 2013b; Vourlidas et al. 2013). Much of this stems from remote-sensing and in-situ observations giving very different aspects of these huge structures (e.g., see detailed discussion from Rouillard 2011) and from transformations a CME may experience during its propagation through interplanetary space (e.g., Dasso et al. 2007; Manchester et al. 2017). As we have outlined earlier in this section, even non-interacting ICMEs can exhibit quite complex internal structure. In particular we emphasized in Sect. 2.3 that MC and ICME boundaries do not often coincide. The non-MC parts of the ICME (i.e., front and rear regions in Fig. 7) can represent the deformed parts of the intrinsic CME flux rope, or as suggested by different compositional/charge state signatures, the parts of the eruption that originate from different regions at the Sun (see discussion in Kilpua et al. 2013b). It is a significant future challenge to shed light on the origin and formation of ICME substructures.

Another interesting question related to CME–ICME connection is the paucity of cold prominence plasma in in-situ observations. We mentioned above that the bright core in coronagraph images corresponds to the prominence material, and indeed, a significant fraction of CMEs are associated with erupting prominences/disappearing filaments (e.g., Munro et al. 1979; Schwenn 1983; Bothmer and Schwenn 1994; Webb and Howard 2012). However, as reported e.g., by Lepri and Zurbuchen (2010) only a small fraction of ICMEs show traces of prominence material. In their study this was the case only for 4% of the analyzed 283 ICMEs. Remote-sensing observations suggest that in most cases the dense prominence material falls back to the Sun and is not carried away within the CME (e.g., Vourlidas et al. 2013). Alternative possibilities have also been presented: Lepri and Zurbuchen (2010) suggested that heating near the Sun may erase low charge state signatures. The authors (see also Gruesbeck et al. 2012) noted that those ICMEs that show low charge states simultaneously contain hot ions. Filament material may also be pushed from the back of the CME flux rope forward, as the CME decelerates when it propagates away from the Sun. This dynamic process was first suggested by Manchester et al. (2014) based on 3-D numerical magnetohydrodynamic (MHD) simulations. Later, Wood et al. (2016) demonstrated this observationally by presenting two case studies where prominence plasma was tracked to the Earth using heliospheric imagers. The prominence plasma showed very little deceleration as it moved towards the leading edge of the CME.

## Modelling of magnetic clouds

This section discusses the in-situ observation-based modelling of MCs that is a key method in ICME studies. The model that gives the best fitting result can shed light on the nature, kinematics and global morphology of ICMEs. In addition, modelling provides many key MC parameters, such as the sign of the magnetic helicity, orientation of the flux rope axis and the impact parameter of the spacecraft through the ICME. We will first discuss basic modeling approaches and thereafter the Grad–Shafranov reconstruction. We continue by introducing the constraints that modeling of MCs puts on the global structure of ICMEs. Finally, we will discuss the magnetic twists in MCs.

### Basic modelling approaches

There is a variety of MC modelling approaches in the literature and we already at this point emphasize that *currently none of the existing models is applicable to all cases*. When selecting the model one should carefully consider the situation in question and the aspects that are particularly desirable to capture. Among the key questions are, for example, is the MC static or expanding? Is its cross-section strongly elongated or more circular? Are plasma effects important, i.e., whether a force-free or non-force-free model would be more appropriate?

Shortly after MCs were first identified in the solar wind, Goldstein (1983) suggested that they can locally be described in the first approximation as cylindrically symmetric flux ropes with force-free magnetic fields, i.e., they fulfill $\nabla \times {\mathbf {B}} =\alpha (\mathbf{r })\,\mathbf{B }$, where ${\mathbf {B}}$ is the magnetic field vector and $\alpha$ is a function of position. One of the earliest and still widely used models assumes that the electric current density depends linearly on the magnetic field, i.e., $\alpha =$ constant everywhere (Burlaga 1988). The solution of such configuration was given by Lundquist (1950) in terms of zeroth and first order Bessel functions ($J_0$ and $J_1$):

2 $$\begin{aligned} \text{ radial } \text{ component: } B_{R}= & {} 0, \end{aligned}$$

3 $$\begin{aligned} \text{ axial } \text{ component: } B_{A}= & {} B_0J_0 \left( \frac{\alpha _0 r}{r_0} \right) {,}~\text{ and } \end{aligned}$$

4 $$\begin{aligned} \text{ tangential } \text{ component: } B_{T}= & {} H B_0J_1 \left( \frac{\alpha _0 r}{r_0} \right) {,} \end{aligned}$$

where *r* is the radial distance from the axis, $r_0$ is the radius of the MC and $B_0$ is the maximum of the magnetic field strength at the centre of the flux rope ($r=0$). $H = \pm 1$ defines the sign of magnetic helicity. This solution describes a magnetic flux rope where the pitch angle of the field lines increases from the axis towards the boundaries and where the field magnitude peaks at the centre. As discussed by Burlaga (1988), constant-$\alpha$ force-free field is a very special configuration in space plasmas. It is a state of the lowest magnetic energy in ideal MHD (e.g., Taylor 1986) where a plasma with finite resistivity bounded by perfectly conducting walls evolves (e.g., Woltjer 1958).

**Fig. 12:**
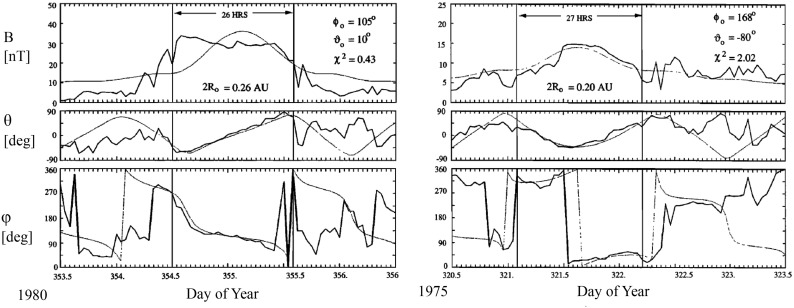
Fitting of the force-free field cylindrical symmetric constant-$\alpha$ model by Burlaga (1988) to the observed magnetic field data using the least-squares fitting method developed by Lepping et al. (1990). The panels show (top) magnetic field magnitude, and the magnetic field (middle) latitude and (bottom) longitude in solar ecliptic coordinates. $\phi _0$ and $\theta _0$ denote the flux rope axis orientation from the fitting, and $2\,R_0$ is the radial diameter of the flux rope. $\xi ^2$ is the parameter that is related to the goodness of the least-squares fit of the magnetic field data in the model (the smaller the value, the better the fit). Image reproduced by permission from Lepping et al. (1990), copyright by AGU

Lepping et al. (1990) developed a least-squares technique to fit the Lundquist solution to the magnetic field observations. Figure 12 shows the results for two MCs. While in both cases the model explains satisfactorily the directional changes of the magnetic field, the agreement with the field magnitude is clearly not so good, in particular in the left-hand part where the measured field profile is almost flat while the solution peaks near the centre of the flux rope. As discussed by Burlaga (1988) and Lepping et al. (1990), asymmetries in the observed profiles likely result from the interaction between the MC and the ambient solar wind, either with the slower solar wind ahead or compression by a faster wind behind.

**Fig. 13:**
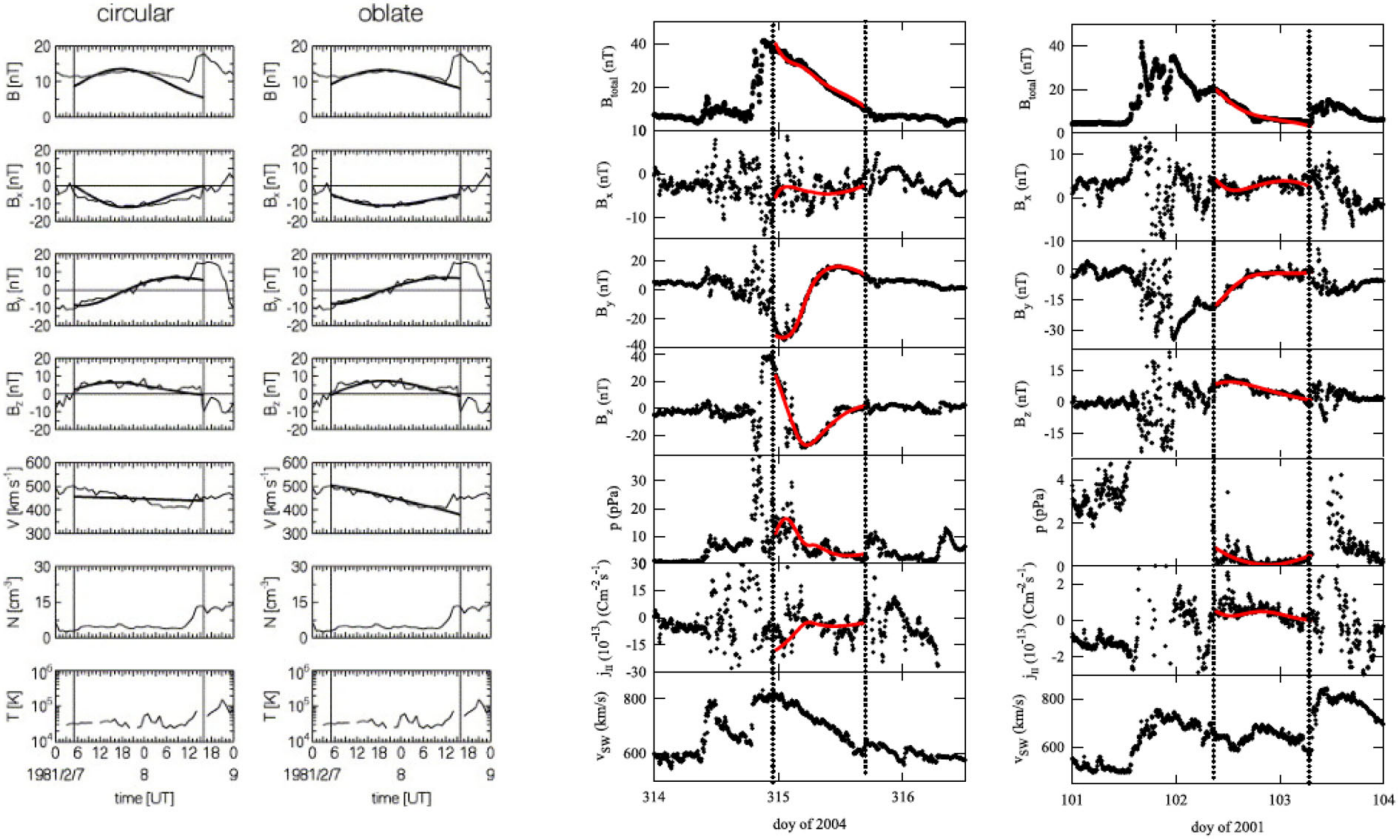
(Left) Results of the fitting of a magnetic cloud on February 7, 1981 using the Lundquist (“circular”) and elliptical (“oblate”) models (the smooth black lines). Both models include the effect of the expansion. (Right) Fitting results for two magnetic clouds from a non-force-free model that incorporates also the hydrostatic plasma pressure and the proton current density in the fitting procedure (the smooth red lines). Images reproduced by permission from [left] Vandas et al. (2006), copyright by COSPAR; [right] Hidalgo (2016), copyright by AAS

As discussed in Sect. 2, MCs generally have oblate cross-sections and they expand strongly. A generalisation of the above described Lundquist solution to elliptical cross-section is presented, for instance, by Vandas and Romashets (2003). The expansion is usually included by searching for a solution of the ideal MHD equations for self-similar expansion in the radial direction and assuming that the energy transport can be described with a polytropic relationship (e.g., Osherovich et al. 1993, 1995). Purely radial expansion of cylindrically symmetric flux ropes is, however, only possible when the electron polytropic index ($\gamma _e$ 7) is less than one. Although observations indeed show that the electron temperature and density anti-correlate within MCs, the temperature actually does not increase as the cloud expands (see discussion on this topic and references therein from Gosling 1999). Instead the negative correlation emerges from the relative enhancement of the halo component to the total density and the plasma’s tendency to achieve local pressure balance with its surroundings (e.g., Riley et al. 2001; Nieves-Chinchilla and Viñas 2008). When MCs are allowed to expand also along their axis, the expanding solutions are obtained for arbitrary values of $\gamma _e$ (e.g., Shimazu and Vandas 2002).

When plasma pressure is important for the evolution of the MC, a non-force-free model may be a more appropriate choice. In particular, the front and rear parts of MCs, which interact with the ambient solar wind, show increased perpendicular current (e.g., Hidalgo et al. 2002; Möstl et al. 2009). An example of a non-force free model that can be fitted simultaneously to the magnetic field and plasma pressure data is the Hidalgo (2016) model. This model also includes the elliptical cross-section and the MC expansion (for the earlier versions of this model, see references in Hidalgo 2016). The overall geometry is described in a toroidal coordinate system where Maxwell’s equations are analytically solved.

The left-hand panel of Fig. 13 shows the results from force-free circular and oblate elliptical models. Both models fit the directional changes in the magnetic field, while the model with the elongated cross-section captures clearly better the magnetic field and the speed profile. The right-hand panel shows how well the above described non-force-free oblate elliptical (Hidalgo 2016) model can fit even relatively complex magnetic field profiles and it captures also the overall trends in the plasma pressure and in the parallel electric current.

Interesting alternative approaches to model MCs are provided by the models that are based on defining an analytical presentation of the 3D shell of the CME/ICME and populating the shell with a magnetic field (e.g., Isavnin 2016). Such approach allows applying all major global deformations (e.g., pancaking, expansion, deflection, rotation) in a straightforward way and is applied to ICMEs identified from remote sensing or in-situ data. Fitting the model to the magnetic field data yields the set of key parameters, such as the MC orientation, ratio of the poloidal and toroidal heights of the flux rope loop, its tilt angle and half width, pancaking angle and front flattening coefficient.

### Grad–Shafranov reconstruction

One of the most widely used non-force-free methods to study MCs is the Grad–Shafranov reconstruction (GSR) (e.g., Hu and Sonnerup 2002; Möstl et al. 2009; Isavnin et al. 2011). GSR uses in-situ measurements of the plasma and magnetic field, and the flux rope is assumed to have 2.5 dimensions, i.e., it has a translational symmetry with respect to an invariant axis direction. The GSR is performed by numerically solving the Grad–Shafranov equation:

5 $$\begin{aligned} \frac{\partial A}{\partial x}+\frac{\partial A}{\partial y} =-\mu _{0} \frac{\mathrm {d}}{\mathrm {d}A} \left( p+\frac{B^{2}_{z}}{2\mu _{0}}\right) {,} \end{aligned}$$

where ${\mathbf {A}}$ is the magnetic vector potential, such that ${\mathbf {A}}=A(x,y)\hat{{\mathbf {z}}}$, and the magnetic field vector is ${\mathbf {B}}=\left[ {\partial A}/{\partial y},-{\partial A}/{\partial x},B_{z}(A)\right]$. The plasma pressure, the pressure of the axial magnetic field component and thus their sum $P_t=p+{B^{2}_{z}}/{2\mu _{0}}$ (transverse pressure) are functions of *A* alone.

**Fig. 14:**
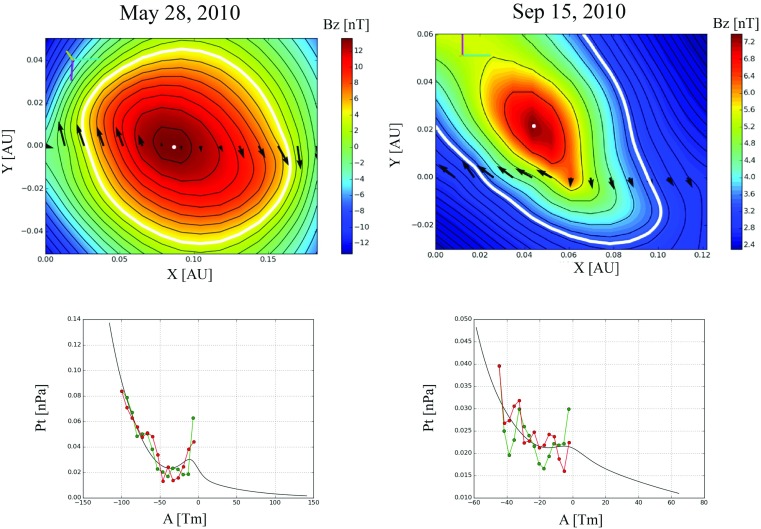
Examples of Grad–Shafranov reconstruction of two magnetic clouds observed by the Wind spacecraft. (Top) Flux rope cross-sectional shapes in the plane perpendicular to the invariant axis. The black arrows show the spacecraft magnetic field observations projected on the same place. The white thick countour corresponds to the flux rope boundary and the white dot the axis of the rope. The colour coding gives the magnitude of the magnetic field component along the invariant axis direction (*z*). (Bottom) $P_t(A)$ curves. The red (green) curves represent *A* along the inbound (outbound) parts of the spacecraft trajectory through the MC. The results are from the European Union’s FP7 project HELCATS online catalogues (http://helcats-fp7.eu)

The top part of Fig. 14 shows the cross-sections of two flux ropes in the plane perpendicular to the invariant axis. The colour coding gives the magnetic field magnitude along the direction of the invariant axis. The thick white line outlines the boundary of the flux rope, outside of which the GSR reconstruction is not reliable. The determination of the invariant axis direction is a critical point in this reconstruction technique. It is based on the assumption of constant magnetic vector potential and transverse pressure on common magnetic field lines. The $P_t(A)$ curve consists of two branches corresponding to the inbound and outbound paths of the spacecraft trajectory through the flux rope. The optimal direction of the invariant axis is found when the inward and outward branches coincide best (see the bottom part of Fig. 14).

A clear strength of the GSR method is that it reconstructs the cross-sectional shape of the flux rope and determines its boundaries. The model also gives estimates of the sign for the flux rope helicity, axis orientation and the impact parameter. However, this technique has severe limitations that must be acknowledged. Since the method assumes that the same equipotential field lines are traversed both during the inward and the outward journey, GSR cannot describe well interacting MCs that may have significantly distorted cross-sections (e.g., Isavnin et al. 2011). GSR also typically severely underestimates the elongation of the MC’s cross-section (e.g., Riley et al. 2004; Möstl et al. 2009; Kilpua et al. 2011; Isavnin et al. 2011). In addition, Al-Haddad et al. (2013) noted that GSR may reconstruct from single-spacecraft data a helical flux rope with a smoothly rotating magnetic field even if the structure is not in fact helical. Nevertheless, when these shortcomings are kept in mind, GSR can be a very powerful tool in identifying and analysing the properties of the “unperturbed” part of the flux rope (see discussion in Sect. 2.2).

### Global structure of magnetic clouds from modelling

**Fig. 15:**
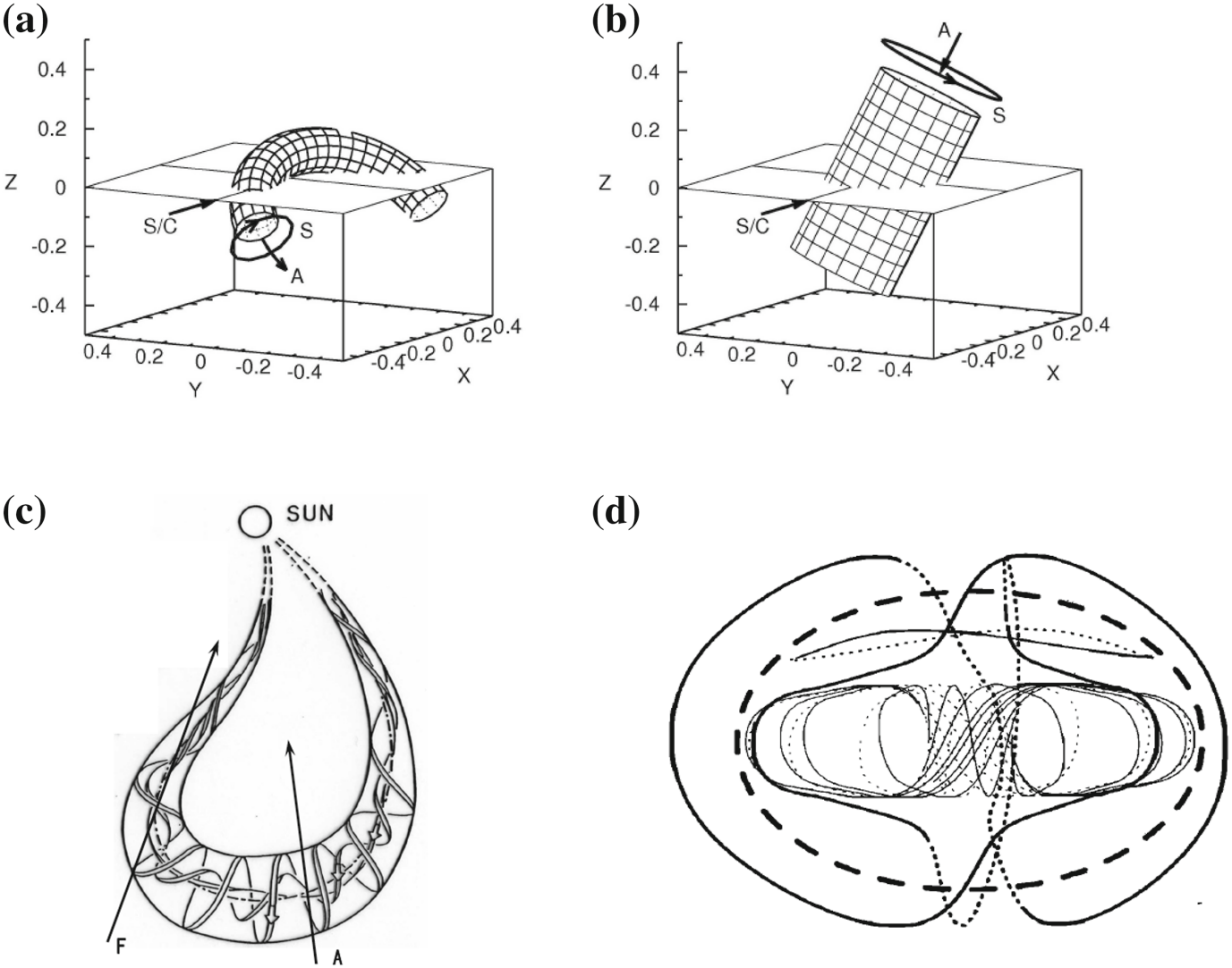
Geometries for a magnetic cloud of 19 March 2001 as obtained from **a** torus model and **b** cylindrical model. The arrows denote the direction of magnetic field on the surface of the (S), the direction of the axial field, and (A) and the spacecraft trajectory (S/C). **c** Curved flux rope loop featuring the central but (path A) and the flank encounter (path F) with a double crossing through the axis. **d** Spheroidal oblate flux rope. The solid (dashed) lines depict the magnetic field lines above (below) the plane of the figure. The thick line shows the reference ellipsoid. Panels (**a**)–(**c**) reproduced by permission from Marubashi and Lepping (2007), copyright by the authors; panel (**d**) from Vandas et al. (1993), copyright by AGU

As discussed in Sect. 2.5, there is convincing observational evidence that MCs are part of huge curved flux rope loops. Local curvature of the axis of the MC can be taken into a account by using a toroidal geometry (e.g., Ivanov et al. 1989; Romashets and Vandas 2003; Vandas et al. 2015; Hidalgo 2016). This geometry is particularly useful when the flux rope loop is traversed through the flank/leg. Path F in Fig. 15c shows the schematic of a flank encounter through the ICME flux rope. In this case the spacecraft may pass twice through the axis of the same flux rope (e.g., Rees and Forsyth 2004; Marubashi and Lepping 2007). However, as shown by Owens (2016), observations of double flux rope encounters are relatively rare. He suggests that this paucity of double flux rope encounters could be explained if the legs of the flux rope form bundles of highly stretched and curved field lines rather than helical fields. He also points out that the legs nonetheless exhibit many of the usual ICME signatures, such as elevated magnetic fields (as they should enclose the same flux as the frontal part of the flux rope loop), reduced proton temperatures and counter-streaming electrons.

The choice of global morphology can have a drastic effect on the fitting results and their interpretation. To illustrate this we show in the top panels of Fig. 15 the geometries for an MC observed on 19 March 2001 from the cylindrical and toroidal model fittings by Marubashi and Lepping (2007). The authors showed, by estimating the difference between the observed and calculated fields, that for this event the torus model described very precisely the magnetic field data within the cloud, while the cylindrical model gave unsatisfactory results. Mulligan and Russell (2001) presented a case where Pioneer Venus Orbiter (PVO) and ISEE 3 observed an MC almost simultaneously on August 27, 1978. A cylindrical model suggested that the spacecraft detected two separate MCs, while the non-cylindrical fitting yielded a stretched cross section that suggested that both spacecraft detected the same MC.

Alternative approaches to global MC configuration have also been presented. Some authors argue that the observed field rotation in MCs would not necessarily reflect helical flux rope structure at all. Instead, the rotation could be explained due to significant writhe in the field resulting from the reconnection low in the corona between the erupting CME fields and the surrounding fields (e.g., Jacobs et al. 2009; Al-Haddad et al. 2011). Another possibility is a closed spheroid shown in Fig. 15d. Spheroidal models can fit the magnetic field data as well as the flux rope models and capture many complexities of the magnetic field profiles, such as sinusoidal, double peaked and plateau type profiles (e.g., Vandas et al. 1993). However, as mentioned above, observations give strong support for flux ropes curving back and being attached to the Sun, and as shown before, when the expansion and elliptical cross-sections are allowed, flux rope models can also explain many of the asymmetries. Multi-spacecraft based modelling has also given convincing evidence for the flux rope structure. For instance, Möstl et al. (2009) and Liu et al. (2008) demonstrated that the Grad–Shafranov reconstruction at one spacecraft could predict successfully the observations at another spacecraft.

Multi-spacecraft modelling studies have also questioned whether the orientation of the flux rope axis is a global property and whether it is maintained during the flux rope evolution. For instance, Möstl et al. (2012) showed that the MC of August 2010 had clearly different axial inclinations at Venus Express and STEREO-B, which were separated by about $20^{\circ }$ in longitude at that time. Another particularly interesting case was presented by Good and Forsyth (2016) who showed that the ICME on November 2011, first detected by MESSENGER at 0.3 AU, had rotated several tens of degrees both in longitude and latitude when it reached the radially-aligned STEREO-B around 1 AU. These observations point to the change from the classical picture of ICMEs as coherent huge flux ropes to more “warped” structures. It is also possible that ICMEs have evolved in time between the observation points, but the most dramatic rotation should on average occur within the first few tens of solar radii of the CME’s journey from the Sun (e.g., Isavnin et al. 2014).

### Twist in the magnetic clouds

Recent statistical studies (e.g., Hu et al. 2015; Wang et al. 2016) suggest that the distribution of the magnetic field line twist is clearly in contradiction with the Lundquist model discussed in Sect. 3.1. Instead of increasing monotonically from the axis, the twist appears to be more or less uniform throughout the cloud. Möstl et al. (2009) analyzed an MC observed on May 20–21, 2007 using the Grad–Shafranov reconstruction and found that the twist increased outwards only very close to the axis of the cloud. At larger distances from the centre the twist declined, i.e., the behaviour was opposite to that of the Lundquist model.

**Fig. 16:**
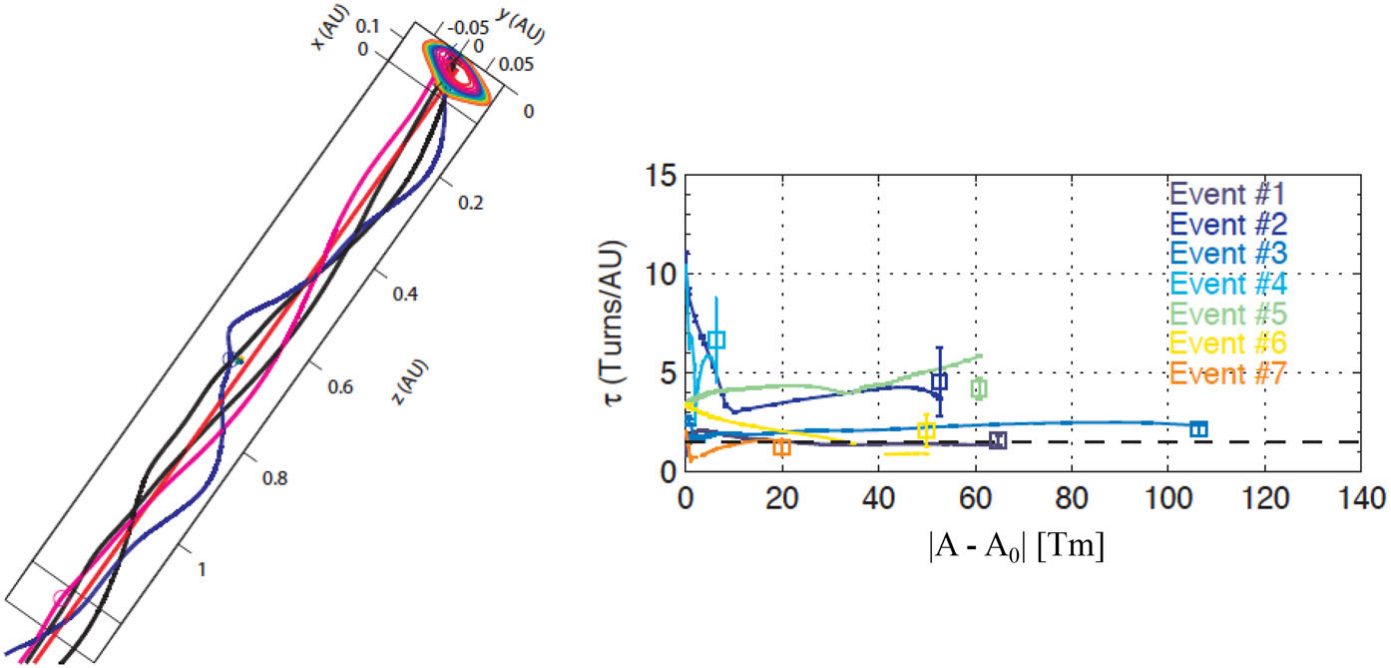
(Left) Examples of field line turns in a magnetic cloud of August 30, 2004 (Event 7 in the right-hand plot). (Right) The average field line twist versus the shifted flux function $|A - A_0|$, where $A_0$ is the value at the flux rope centre and *A* is the GSR result. The square indicates the mean for each of the curves, and the error bars give the standard deviations. Image reproduced by permission from Hu et al. (2015), copyright by AGU

Hu et al. (2015) arrived at similar conclusions by utilising the field-line path lengths estimated from velocity dispersion in the arrival of solar energetic particles as well as from Grad–Shafranov reconstruction together with a constant-twist nonlinear force-free Gold–Hoyle flux rope model (Gold and Hoyle 1960). The left-hand part of Fig. 16 shows an example of the twisting field lines for one of the analysed events and the right-hand part gives the average twist as a function of the magnetic flux $|A - A_0|$ (see definition of *A* from Sect. 3.2) for seven MCs. *A* increases towards the axis of the flux rope and $A_0$ denotes its value at the axis (i.e., at the centre of the flux rope $|A - A_0|= 0$). The twist is given in the units of turns per AU. The typical amount of twist found near the Earth orbit corresponds to the field lines making few turns around the axis of the MC over the distance of one AU. The right-hand part of Fig. 16 shows that for all analysed events the twist remains nearly constant throughout the cloud. Larger twists are observed for some events in the core of the clouds, similar to what was found by Möstl et al. (2009).

As discussed by Wang et al. (2016), the magnetic field twist is an intrinsic property of flux ropes that is strongly connected to their stableness, and hence to the formation of CMEs. When the total twist of flux ropes at the Sun increases sufficiently high (1.25 turns about the axis for a line-tied force-free flux rope having a constant twist), the flux rope becomes kink unstable and can erupt as a CME (e.g., Hood and Priest 1981; Török and Kliem 2005). Hence, twists in interplanetary MCs exceed the above-stated critical value. Direct comparison of critical twist values between the Sun and in interplanetary space is, however, not straightforward due to highly different conditions in the corona and in interplanetary space, and evolution of flux ropes from Sun to Earth (see discussion, e.g., in Burlaga 1988 and Wang et al. 2016). Wang et al. (2016) also remark that the twist can also vary within the ICME flux rope if it is composed of parts that have different origin at the Sun. Part of the flux rope may be present already before the CME eruption and a significant amount of magnetic flux and twist can be added to it during the eruption process (e.g., Qiu et al. 2007; Temmer 2017).

## ICME shocks and sheaths

This section focuses on the sheaths and shocks associated with ICMEs. We begin with discussing how ICME shocks and sheaths form in interplanetary space. Next, we will present solar wind properties in ICME sheaths and compare them to ICME properties, followed by a discussion on main structures that can be found in the sheaths. We conclude this section with a brief review of acceleration of solar energetic particles by interplanetary shocks.

### Formation of ICME shocks and sheaths

**Fig. 17:**
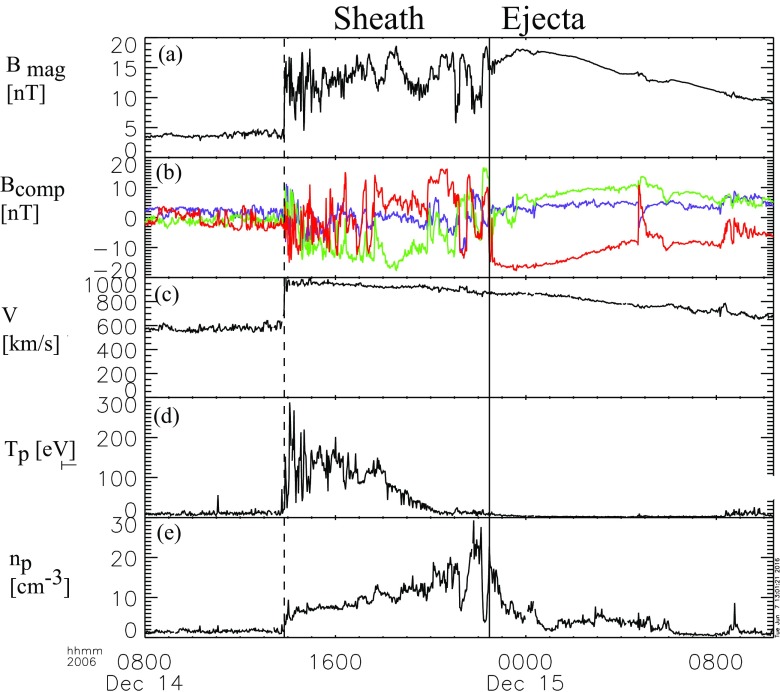
An example of a sheath region between a forward shock and an MC. The panels show from top to bottom: *a* the magnetic field magnitude, *b* components of the magnetic field in GSE coordinates ($B_X$: purple, $B_Y$: green, $B_Z$: red), solar wind *c* speed, *d* proton temperature, and *e* proton density. The dashed line shows the shock and the solid line the leading edge of the ICME. The measurements are from the ACE spacecraft. Data have been obtained from the ACE Data Center (http://www.srl.caltech.edu/ACE/ASC/)

Figure 17 shows a clear fast forward shock (dashed line) driven by an MC observed on 14–15 December, 2006 in the near Earth solar wind. The magnetic field magnitude, as well as solar wind speed, density and temperature all increase abruptly at the shock (dashed line). The sheath region, as defined in the Introduction, extends from the shock to the ICME leading edge (solid line). For an ICME to drive a fast forward shock its speed in the solar wind frame must exceed the speed of the fast MHD wave, which in the direction of the ambient magnetic field is the Alfvén speed ($v_A$) and perpendicular to the magnetic field the magnetosonic speed ($v_{ms}$). Thus the respective Mach numbers (i.e., here the ratio of the speed of the ICME to the local “information speed” in the solar wind) $M_A$ or $M_{ms}$ calculated in the solar wind frame are important dimensionless parameters.

Also ICMEs that propagate faster than the preceding solar wind, but not fast enough to drive a shock, deflect and compress the plasma flow ahead and have disturbed sheath-like regions. They are often preceded by leading waves that have not steepened to fully-developed shocks.

As the speed of the ambient solar wind and the average ICME speeds (see Sect. 2.3) are roughly in the similar range 300–800 km s$^{-1}$, most interplanetary shocks are not particularly strong. For example, Kilpua et al. (2015b) found that the annual medians of $M_{ms}$ during the period 1995–2013 did not exceed 4. This study was based on the open-access interplanetary shock database^8^ that has recently been released at the University of Helsinki. For example, the only $M_{ms}$ exceeding 10 in the data base (as of October 2016) are April 28, 2001 (12.6 measured by Wind) and March 27, 1979 (11.7 measured by Helios-B at the distance 0.75 AU from the Sun).

We note that shock databases are often incomplete in the sense that events have to be left out for various reasons. For example, extreme events may be physically complex and the instruments may work close to or beyond saturation, while strong energetic particle radiation may contaminate plasma detectors, leading to difficulties in calculating critical parameters, such as speed, temperature and density. For example, the shock associated with the extremely fast and strong ICME detected at STEREO-A on 23 July 2012 (e.g., Liu et al. 2014) requires a more detailed analysis. Using reasonable assumptions and interpretation of observed data, Riley et al. (2016) found Mach numbers $M_A=21$ and $M_{ms}=17$ for this shock. Russell et al. (2013) noted that for this event the pressure from the energetic particle component exceeded both the thermal and magnetic field pressures. The shocks where energetic particles dominate the pressure are rare, but occasionally observed near 1 AU. Lario et al. (2015) found additionally five such shocks from STEREO-A data over 6 years of observations (2009–2014). As suggested by Russell et al. (2013) and Lario et al. (2015) energetic particles may modify shock properties when compared to cases without significant energetic particle effects.

ICME sheaths form due to both CME propagation relative to solar wind and expansion of the CME (e.g., Kaymaz and Siscoe 2006; Siscoe and Odstrčil 2008). We return to discuss the characteristics of propagation and expansion sheaths in Sect. 4.3. It is also worth noting that ICME sheaths accumulate gradually over long time periods and carry history of the interaction with the solar wind over a range of heliocentric distances (Siscoe and Odstrčil 2008).

### Sheath signatures and properties

ICME sheaths are distinctly different from the ICME proper (sometimes referred to as ejecta). Different solar wind properties in these structures are highlighted in Fig. 17. The figure illustrates that the magnetic field variations are considerably larger, and the temperature and density much higher in the sheath than in the MC.

**Fig. 18:**
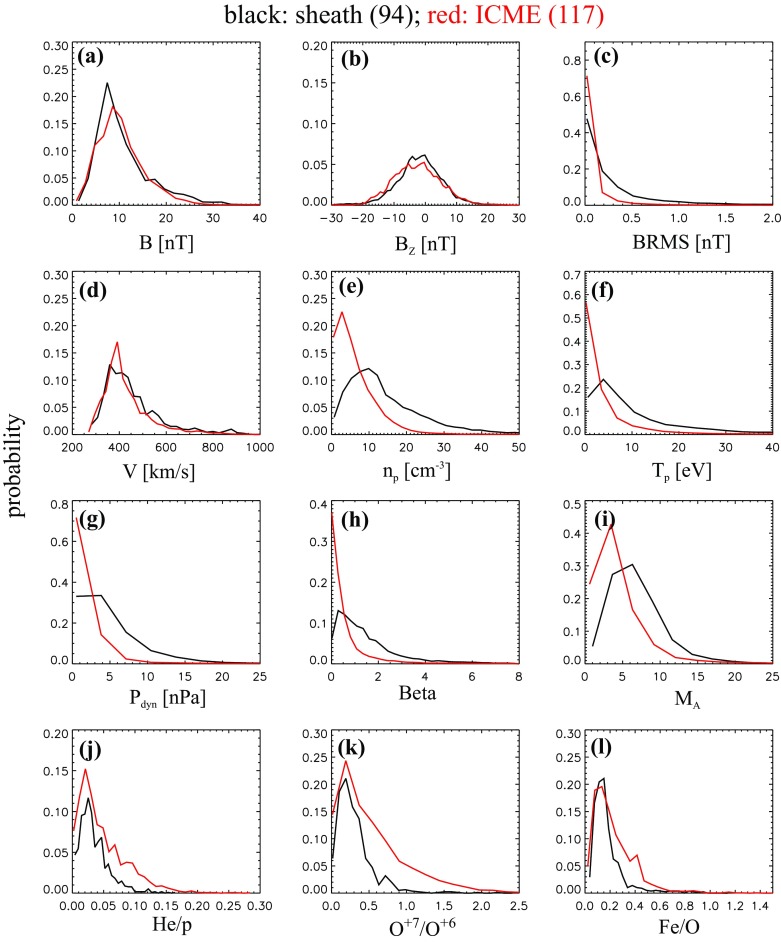
Probability distributions of various solar wind parameters in ICME sheaths (black) and ICME (red). The panels give: **a** magnetic field magnitude, **b** IMF north-south component, **c** root-mean-square of the magnetic field, solar wind **d** speed, **e** density, **f** temperature, **g** dynamic pressure, **h** plasma beta, **i** Alfvén Mach number, and **j** alpha to proton (He$^{++}$/p), **k** O$^{+7}$/$^{+6}$, and **l** Fe/O ratios. Panels **a**–**i** The data sets are 5-min OMNI data, panel **j** 1-h OMNI data and panels **k**–**l** are 1-h (2-h after August 2011) ACE/SWICS data. The numbers in parenthesis show the number of sheaths and ICMEs

**Fig. 19:**
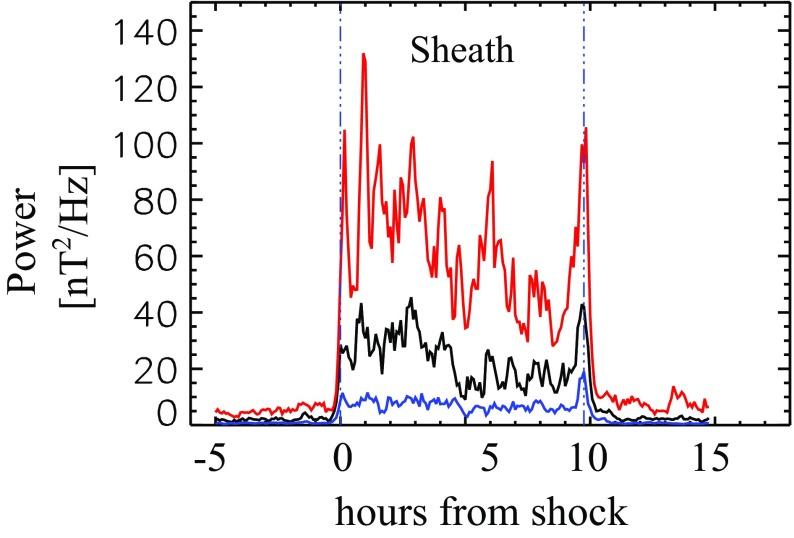
ULF power of the north-south component of IMF in ICME sheaths. The black line is the median. The red and blue lines are upper and lower quartiles, respectively. The length of all sheaths is scaled to 10 h (see Kilpua et al. 2013a)

Figure 18 gives probability distributions for IMF intensity, its southward component, and several solar wind parameters calculated using the event list of Kilpua et al. (2015a). Only the magnetic field and velocity distributions (panels 18a, b, d) are rather similar for sheaths and ICMEs, but all other parameters show significant differences. First, sheaths are clearly more turbulent than the ICMEs. Panel 18c shows that the distribution of the root-mean-square of the magnetic field magnitude has a pronounced tail in sheath regions. Different fluctuation levels are also visible in Fig. 19 that shows superposed epoch analysis of Ultra Low Frequency (ULF) fluctuations in the IMF north-south component over the 3–10-min range for 41 sheath $+$ ICME-events during Solar Cycle 23 (Kilpua et al. 2013a). The transition from the sheath to ICME is featured by a clear and abrupt drop in the fluctuation power.

Other key differences are related to sheaths being compressed and often shocked structures, while the ICMEs typically expand strongly. This explains considerably higher temperatures and densities (and consequently higher dynamic and plasma pressure) in the sheaths (panels d and e in Fig. 17, and panels e and f in Fig. 18). In addition, although magnetic field distributions are relatively similar, due to much higher plasma pressure the sheaths have typically much larger plasma beta than ICMEs (panel 18h). Larger densities also lead to larger dynamic pressure and Alfvén Mach number in sheaths (panels 18g, i). These parameters are of paramount importance for solar wind–magnetosphere coupling and we return to these in Sect. 6.

The last three panels of Fig. 18 give the alpha to proton ratio, the O$^{+7}$/O$^{+6}$ ratio and the iron to oxygen ratio. As discussed in the previous section, solar wind charge and compositional characteristics are particularly useful to distinguish plasma of different origins. All these parameters peak approximately at similar values in the sheaths and ICMEs, but ICMEs feature clearly more distinct tails. This is expected as sheaths largely form from the “nominal” solar wind plasma that is expected to have shorter confinement times in the corona and/or lower source temperatures than plasma that constitutes CME flux ropes (excluding the possible filament material).

The radial extensions of the sheaths are typically smaller than those of the ICMEs. The mean duration of the ICMEs that were used to compile Fig. 18 was 26.5 h and their mean width 0.29 AU, while corresponding values for the sheaths are 11.1 h and 0.13 AU. Nevertheless, sheaths are also macro-scale interplanetary structures with their dimensions being a significant fraction of the astronomical unit. The thickness of the sheath depends on the speed and physical properties of the driving ICME (e.g., its shape and the radius of curvature), and the shock compression ratio (Russell and Mulligan 2002). In addition, the thickness of the sheath increases from the nose of the ICME towards its flanks. Note that as ICME-driven shocks are generally more extensive than the driving ICMEs (see Fig. 1), in some cases the spacecraft may encounter only the shock and the turbulent sheath behind. In fact, the majority, if not all, “driverless shocks”, i.e., shocks that do not have a clearly identifiable solar wind driver, are associated with ICMEs (e.g., Borrini et al. 1982; Cane 1988; Gopalswamy et al. 2010; Janvier et al. 2014).

The plasma waves and fine-structures in ICME sheaths have not yet been studied extensively. It is, however, expected that both kinetic-scale and fluid processes at the ICME-driven shock, within the sheath itself and at the ICME boundary can provide free energy for waves. Liu et al. (2006a) performed a superposed epoch analysis to show that the plasma in particular in the sheaths of shock driving MCs is generally mirror unstable and there hence should be mirror mode waves. The superposed epoch analysis by Kilpua et al. (2013a) showed that the power of the magnetic field and dynamic pressure ULF waves (see also Fig. 19 for the $B_Z$ ULF power) in ICME sheaths peaks close to the shock and close to the sheath–ICME boundary. Intense wave activity and large amplitude magnetic field fluctuations have been reported in particular downstream of ICME shocks (Kataoka et al. 2005; Kajdič et al. 2012). The neighbourhood of the sheath–ICME boundary is a much less studied region in terms of plasma wave activity. The accumulation of plasma and magnetic field from different sources during the interplanetary formation of the sheath introduces interfaces that are suitable places to find plasma discontinuities and reconnection exhausts (e.g., Kataoka et al. 2005; Feng and Wang 2013). Magnetic reconnection favours low plasma beta conditions ($\beta < 2$) (e.g., Scurry et al. 1994). Although the sheaths are higher beta structures than the ICMEs, according to Fig. 18h their beta distribution is biased to $\beta < 2$ and magnetic reconnection may occur during higher plasma beta conditions if the shear angle of the magnetic field across the current sheet is sufficiently large (Phan et al. 2013). Indeed, Feng and Wang (2013) found five reconnection exhausts within a high-beta sheath of an MC observed on 18–20 October, 1995.

### Large-scale sheath structures

An ICME sheath region is a combination of a “propagation sheath” and an “expansion sheath”. The former refers to a sheath that forms around an object propagating relative to the solar wind and the latter is due to an expanding object. Several of the global properties of the ICME sheaths are similar to the much more closely studied terrestrial magnetosheath. These similarities are characteristic of propagation sheaths, including the diversion of the solar wind flow and draping of the IMF around the ICME as well as the alignment of the magnetic field discontinuities parallel to the leading edge of the ICME (e.g., Siscoe and Odstrčil 2008). Significant differences, however, arise because ICMEs also expand, whereas the planetary sheaths are almost pure propagation sheaths (e.g., Kaymaz and Siscoe 2006; Siscoe and Odstrčil 2008), disregarding the relatively small expansions and contractions in response to variations in the solar wind pressure. The expansion sheath characteristics listed by Siscoe and Odstrčil (2008) include lower deflection speeds relative to the incident flow and smaller sheath thickness (about 50% less) relative to the radius of curvature of the object/driver. In addition, the solar wind plasma accumulates at the ICME leading edge because the deflection is not strong enough to overcome the expansion to allow all plasma to flow around the ICME. This means that ICME sheaths are composed of layers of inhomogeneous IMF and plasma that accumulates as the CME propagates through the solar wind.

**Fig. 20:**
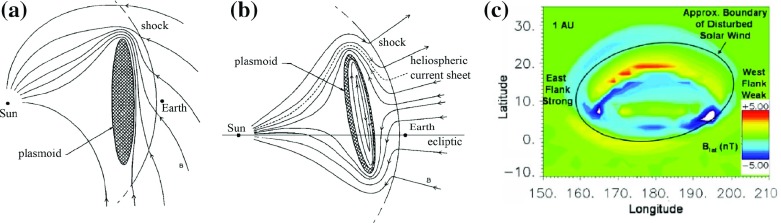
Draping of the IMF around the ICME. Sketch of the draping of the IMF around and ICME in **a** the ecliptic plane and **b** the meridional plane. **c** Global MHD simulation results of a Parker spiral type IMF draping. The strength of the out-of-ecliptic IMF component is shown for a view towards the Sun (ignore the part within the oval that belongs to ICME). Panels (**a**) and (**b**) reproduced by permission from Gosling and McComas (1987); Panel (**c**) from Siscoe et al. (2007), copyright by AGU

Figure 20 illustrates the above-mentioned IMF draping around the ICME in more detail. First, Fig. 20a illustrates that, in the ecliptic plane, draping and bunching of spiral interplanetary magnetic field lines leads to stronger field at the western flank of the ICME. Figure 20b) in turn shows the draping process in the meridional plane for the purely radial IMF. The direction of the draping here is determined by whether the preceding IMF points towards or away from the Sun and the launch direction of the CME with respect to the observer (the so-called Gosling–McComas-rule, see Gosling and McComas 1987). The MHD simulation results in Fig. 20c) by Siscoe et al. (2007) assume the Parker spiral magnetic field (the spiral angle being on the average $45^{\circ }$ at 1 AU). The plot indicates that the draping creates enhanced out-of-ecliptic field at the eastern flank of the ICME. The results described above demonstrate that the draping can generate out-of-ecliptic magnetic field components from the initially ecliptic fields. Out-of-ecliptic fields, when directed southward, are of great interest for space weather as we will discuss in Sect. 6. The observed signatures and properties of the ICME sheath hence depend strongly on the ambient IMF properties, CME launch direction and how far from the apex the spacecraft encounters the ICME. In addition, both global and small-scale sheath properties are expected to depend on whether the observations are made behind the quasi-parallel or quasi-perpendicular part of the shock, i.e., whether the angle between the shock normal and the upstream magnetic field is smaller or larger than $45^{\circ }$ (e.g., Burgess et al. 2005; Bale et al. 2005).

The draping of the IMF is also one of the primary causes creating “planar magnetic structures” (PMSs) (e.g., Nakagawa et al. 1989) within ICME sheath regions (e.g., Neugebauer et al. 1993). These are periods when the variations of the magnetic field occur parallel to a single plane. PMSs are identified in most sheath regions and they typically cover a considerable part of the sheath (e.g., Jones and Balogh 2000; Palmerio et al. 2016). Another key mechanism that gives rise to PMSs in ICME sheaths is the amplification and alignment of pre-existing solar wind discontinuities at the ICME-driven shock. The formation of PMSs depends on the local plasma properties as well as on the characteristics of the ICME and its leading shock. For instance, PMSs are most frequent behind quasi-perpendicular strong and high-beta shocks and when the ICME expands strongly (e.g., Kataoka et al. 2005; Palmerio et al. 2016). The orientation of PMS planes can also yield information on the large-scale structure of the ICME flux rope and planar parts of the sheath are associated with the strongest out-of-ecliptic magnetic fields (e.g., Palmerio et al. 2016).

Additional large-scale structures worth mentioning in ICME sheath regions are low and high plasma density regions sometimes found just adjacent to the ICME leading edge. Such features can be distinguished from Fig. 17 as a clear dip in density just adjacent to the ICME leading edge (solid line) that is preceded by a density peak up to about 30 cm$^{-3}$. The high density plasma region is called the “Pile-Up Compression” (PUC) region (e.g., Das et al. 2011) and it accumulates already near the Sun, as overlying coronal arcades are dragged by the rising ICME flux rope (see the bright rim in the coronagraph image in Fig. 11). PUCs further develop as the ICME expands during its interplanetary propagation. The regions of low plasma density are also sometimes found in sheaths close to the leading edge of the ICME (e.g., Dasso et al. 2007; Farrugia et al. 2008). They likely reflect different density profiles and shock compressional properties encountered during the propagation of an ICME rather than being formed by plasma squeezing as is the case for plasma depletion layers in planetary magnetosheaths (e.g., Zwan and Wolf 1976).

### Particle acceleration at ICME shocks

**Fig. 21:**
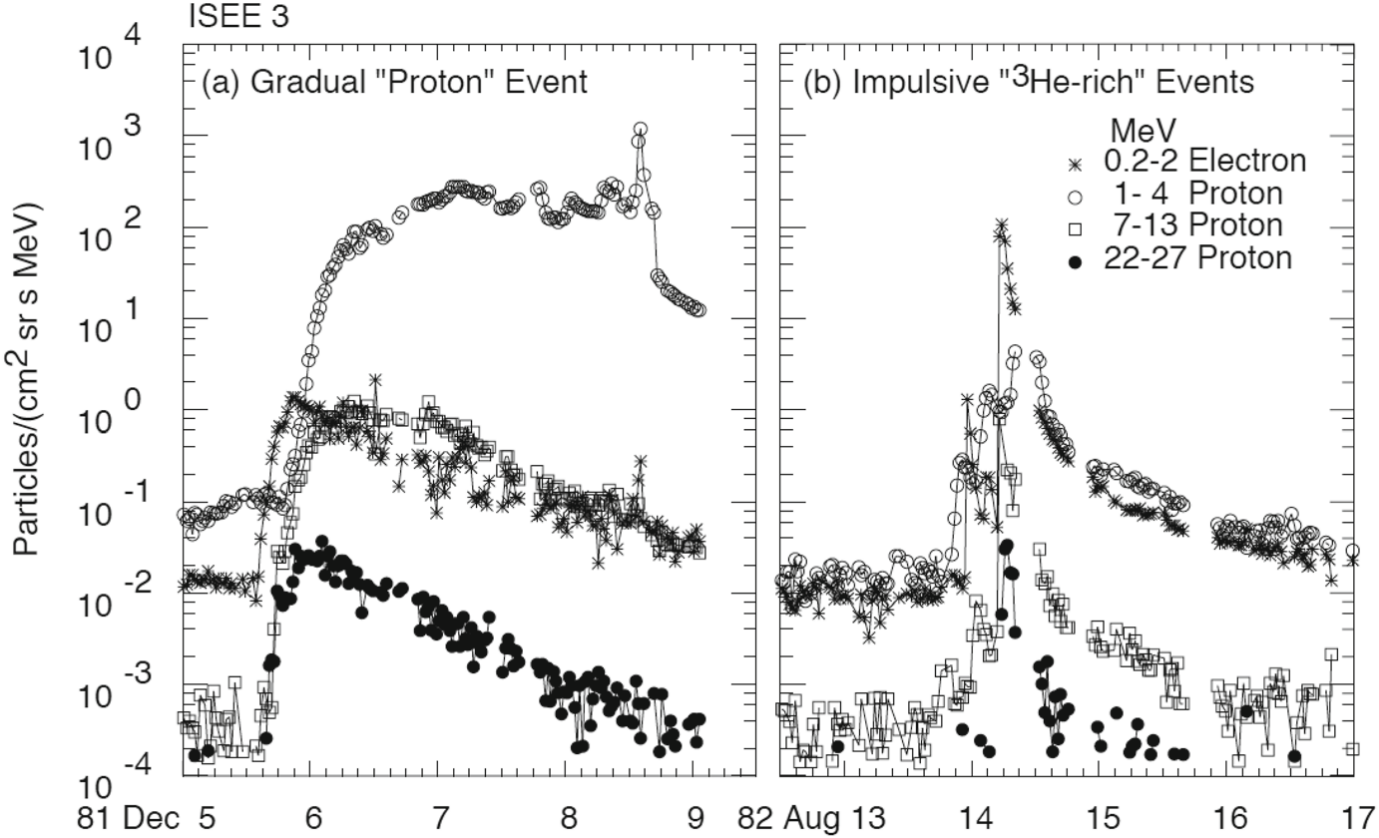
Particle spectra of a gradual SEP event (left) and two consecutive impulsive events (right) as observed by the ISEE 3 spacecraft. Image reproduced by permission from Reames (1999), copyright by Springer

The ICME shocks have an important role in the acceleration of solar energetic particles (SEP). The SEP events are often divided to impulsive and gradual events (e.g., Cane et al. 1986; Reames 1999) as illustrated in Fig. 21. The impulsive events are relatively short-lived, the energetic particles being observable for a few hours. They are electron-rich and the $^3$He/$^4$He ratio is of the order of one, sometimes even more than 10. They are typically associated with impulsive hard and soft X-ray flares but not necessarily with CMEs. The gradual events are proton-rich, they are observable for days and the $^3$He/$^4$He ratio is much smaller than in the impulsive events. The associated X-ray flares are typically soft. Kahler et al. (1984) noted that almost all gradual SEP events are associated with CMEs. This has later been confirmed, e.g., by SOHO and STEREO observations (e.g., Reames 2015, and references therein).

Energetic particles in impulsive events are thought to be accelerated close to the Sun by the rapid energy release in the impulsive phase of a solar flare and by the consequent strong wave activity. High $^3$He content is a curious fact because only some 0.05% of all helium in the solar atmosphere is $^3$He (e.g., Mason 2007). This indicates that the acceleration process must be highly favourable to enrichment of the $^3$He population, more precisely $^3$He$^{++}$, whose gyro frequency is 2/3 of the proton gyro frequency. While the final particle spectra most likely are due to a complicated chain of processes, the role of wave–particle interactions, in particular with Alfvén ion cyclotron waves, is important (e.g., Petrosian et al. (2009), and references therein). Furthermore, Mason et al. (2016) suggest that the same process lies behind the acceleration of heavy elements in impulsive SEP events.

Before CMEs were identified in coronagraph images, both impulsive and gradual SEP events were related to solar flares and shock waves driven by them. Here the solar radio bursts played an important role. Wild et al. (1963) were the first to suggest that protons were accelerated by shock waves associated with Type II radio bursts. Also the original bimodal impulsive–gradual picture, proposed by Cane et al. (1986), was based on flare and radio burst observations, although the authors noted the relation of CMEs and gradual events.

The main ICME-related shock acceleration mechanism is presently considered to be the diffusive shock acceleration (DSA), the theory of which was introduced by Axford et al. (1977) Krymskii (1977), Bell (1978) and Drury (1983). During the acceleration process the accelerated particles must cross the shock several times, gradually gaining energy until they escape from the shock region. Consequently, an observer, say, at 1 AU sees energetic particles emitted from the shock typically all the way until the shock itself arrives. For example, in the left-hand panel of Fig. 21 the energetic particle spectra rise soon after the CME release on the 5th December until the shock arrival on the 8th, indicated by the “shock peak” in the electron and lowest-energy proton fluxes. For a thorough review of the recent developments in shock acceleration theory, see Verkhoglyadova et al. (2015) and references therein.

To facilitate the multiple shock crossings there must be fluctuations both upstream and downstream of the shock that scatter the accelerated particles back to the shock. As the magnetic field close to the Sun is radial, the apex of the shock is almost parallel. This creates a foreshock where the upstreaming particles drive turbulence, which provides the scattering centres. While moving further away from the Sun the shock normal angle tends to become more quasi-perpendicular due to varying direction of the upstream magnetic field, and the shock normal angle is different in different sectors of the expanding shock front (Manchester et al. 2005). The sheath region is turbulent behind both quasi-parallel and quasi-perpendicular shocks (e.g., Bale et al. 2005; Burgess et al. 2005). In particular, sheaths behind quasi-parallel shocks often exhibit strong turbulence, which can lead to rapid acceleration at interplanetary shocks (Zank et al. 2015).

The DSA scenario may look simple but practical self-consistent calculations are far from straightforward (e.g., Verkhoglyadova et al. 2015, and references therein). The ambient plasma parameters and the formation of the suprathermal seed population to be accelerated can be very different from case to case. Furthermore, the spatial and temporal scales in the acceleration region extend from the large-scale MHD shocks to ion and electron kinetic-scale turbulence and individual accelerated particles. Consequently, the applied numerical acceleration models contain several parameters, some of which can only be determined in an ad-hoc manner. As an example of such an approach, Vainio et al. (2014) performed Monte Carlo simulations in their state-of-the-art semi-analytical foreshock model from about 6 to 60 solar radii. More similar studies together with data comparison are called for to gain better understanding of the ICME shock acceleration.

While the details of the acceleration processes still are under intensive research, it is clear that protons and other ions in gradual events can be accelerated to high energies at the shocks driven by fast ICMEs. However, large flares, shocks, CMEs and strong SEP events tend to occur together, although there does not need to be any causal relationship between CMEs and flares. The consequences of this were called the “big flare syndrome” by Kahler (1982). The apparent correlations can lead to a bias in statistical studies because the gradual events are orders of magnitude more intense than impulsive events, whereas the impulsive events are much more numerous. In fact, some studies have suggested that both flare ICME shock acceleration contribute to large SEP events but the relative abundances of different ion populations vary from case to case (e.g., Cane et al. 2003, 2010). A thorough statistical study by Trottet et al. (2015) showed that acceleration by both flares and ICME-driven shocks contributes to the production of tens of MeV-proton and nearly-relativistic electron populations in large SEP events. This conclusion was further supported by Dierckxsens et al. (2015) and Grechnev et al. (2015) who pointed out that the fluxes of highest-energy solar protons ($>60$ MeV) have a stronger correlation with the flare intensity than the CME speed, whereas for lower energies the correlation is the opposite. Consequently, there is no unique division between impulsive and gradual events as the impulsive component can be hidden under the much more intense shock accelerated component during gradual events. For the viewpoint of the present review we do not need to involve ourselves more in this long-standing debate.

A further challenge with SEP events is their east-west variability. Most events have been measured at one or, at most, at a few single points in space and the global picture has been formed by compiling observations of different events with different characteristics together (e.g., Cane et al. 1988). That gradual events can have a wide longitudinal extent is understandable, as ICME-driven shocks are longitudinally wide and expand during the propagation. de Lucas et al. (2011) analyzed all ICME-driven shocks during 1974–1986 that had been observed by multiple spacecraft, including observation by Helios. They found that there was a 50% chance to observe a shock at two locations separated by $90^\circ$ after which the probability quickly dropped, and only in four cases the extent was in the range $120^\circ$–$160^\circ$. However, the energetic particle enhancements can be longitudinally even wider than the shock. This may at least partially be attributed to cross-field diffusion in the interplanetary space but also the shock may have been longitudinally wide near the Sun than in the interplanetary space (e.g., Cliver et al. 1995; Rouillard et al. 2012; Lario et al. 2017, and references therein).

Combining data from STEREO-A and -B and near-Earth spacecraft, a significant number of individual SEP events have been observed at multiple locations (e.g., Richardson et al. 2014; Ebert et al. 2016). A particularly interesting SEP event took place on November 11, 2011, and was observed by near-Earth spacecraft, both STEREO spacecraft and MESSENGER. In this case the energetic particles spread very rapidly from an event behind the east limb to both STEREO spacecraft and to the Earth. According to Gómez-Herrero et al. (2015) the particle enhancement was almost circumsolar although the data indicated that all particles originated from a single active region on the Sun. Their conclusion was that the data were not consistent with propagation by diffusive transport alone, as seems to have been the case in an earlier event on January 17, 2010 (Dresing et al. 2012).

## Variation of ICME and sheath properties during the solar cycle and with location in the heliosphere

In this section we first introduce how the properties and frequency of ICMEs and their sheaths vary with solar cycle and discuss briefly the origin of these variations. We continue by discussing the identification of ICMEs at large heliospheric distances and explaining how ICMEs vary with the radial distance from the Sun and with the heliospheric latitude. We conclude this section by reviewing ICME interactions with other large-scale solar wind structures, such as stream interaction regions, fast streams, heliospheric plasma sheet and other ICMEs as well as with the “quiet” background solar wind.

### Solar cycle variations

**Fig. 22:**
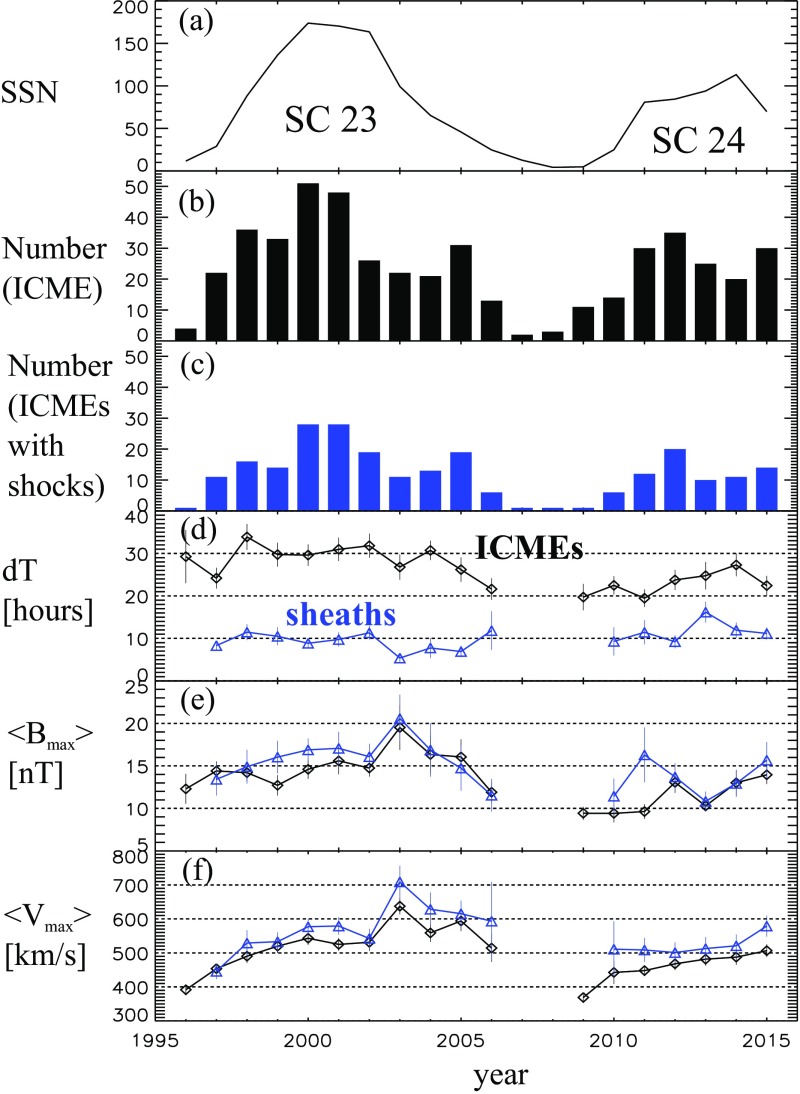
Annual variations in the ICME (black) and sheath (blue) occurrence rate and properties. The panels show *a* the yearly mean sunspot number from Solar Influences Data Center (http://sidc.oma.be), and the annual number of *b* ICMEs, and *c* the ICMEs that drove shocks, the annual means of *d* the duration, and the peak *e* magnetic field, and *f* speed in ICMEs (black) and sheaths (blue). The error bars give the standard deviations. The years when three or less events occurred are excluded from panels (*d*–*f*). The ICME intervals are from the Richardson and Cane ICME list. The sheath intervals are determined using the ICME leading edge times given in Richardson and Cane list and the shocks time from the Interplanetary Shock Database of the University of Helsinki (www.ipshocks.fi/). Note that here we consider as “sheaths” only the cases where an ICME was preceded by a fully developed shock

**Fig. 23:**
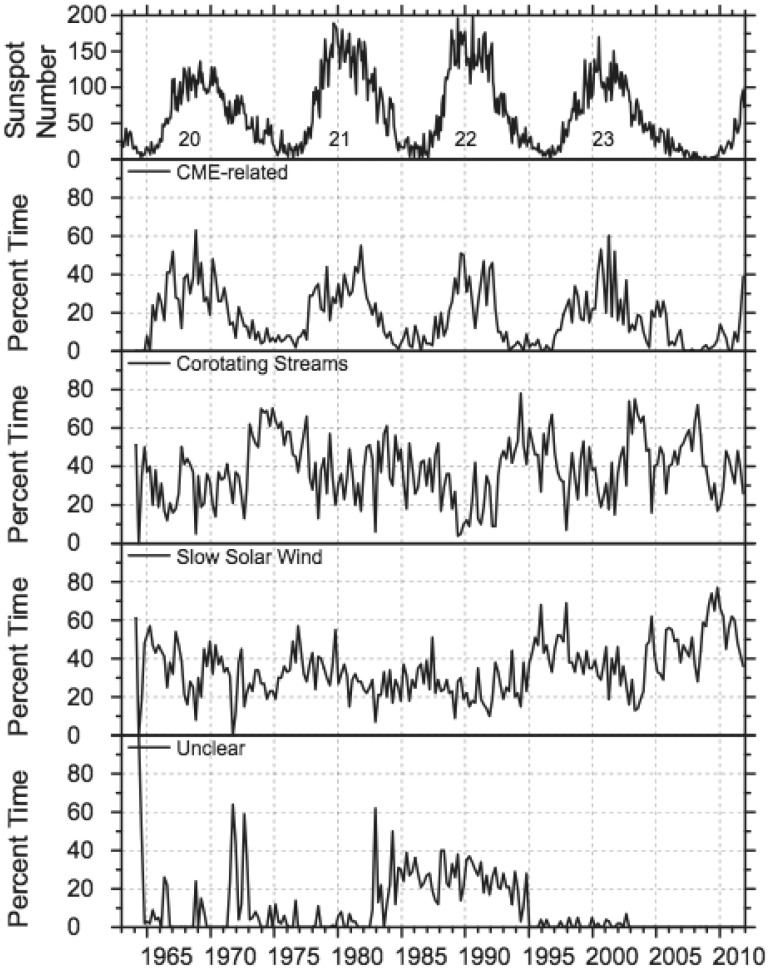
The top panel shows the monthly sunspot number. The next three panels show the percentage (three solar rotation averages) of the solar wind flows associated with ICMEs, high-speed streams and solar solar wind for over more than four solar cycles (1963–2011). The figure is from Richardson and Cane (2012a)

Solar cycle variations in ICME rates and properties have been studied by various authors (e.g., Jian et al. 2006; Richardson and Cane 2012a; Wu and Lepping 2016). The variations in sheath properties are, however, much less investigated. In Fig. 22 we show the number of ICMEs and shock-driving ICMEs, and the average durations, and peak magnetic fields and speeds for ICMEs and sheaths in the near-Earth solar wind over almost two solar cycles (1996–2015).

The three top panels of Fig. 22 show that near solar minimum only a few ICMEs were reported, compared to over 50 events per year at Solar Cycle 23 maximum. The number of shock-driving ICMEs (third panel) also varies roughly in phase with the solar activity cycle. Richardson and Cane (2012a) studied the near-Earth solar wind flows spanning over four solar cycles. Figure 23 from their paper shows clearly the tendency of ICME-related flows (including here contribution from sheaths) to vary in concert with the solar activity cycle. The figure also reveals that near solar maximum ICME-related flows were present up to 40–60% of the time, while decreasing to only 5% near solar minimum.

The correlation between the ICME and CME rates and the sunspot number is, however, known to be non-perfect (e.g., Riley et al. 2006; Richardson and Cane 2012a). For instance, high latitude CMEs frequently deflect towards lower latitudes, in particular near solar minimum (e.g., Cremades et al. 2006; Kilpua et al. 2009), and the variations in the preferred latitudes of the source regions of CMEs, namely streamer belt, active regions and polar crown filaments, also affect the fraction of CMEs that intercept the ecliptic (e.g., Hundhausen et al. 1984; Kilpua et al. 2011).

The bottom three panels of Fig. 22 show that ICMEs (black curves) and sheaths (blue curves) tend to have, on average, longer durations and stronger peak magnetic fields and to be faster near solar maximum than during quiet times. The strongest average peak fields and the fastest speeds are, however, found in the declining phase. These solar cycle trends reflect the solar cycle variations in CMEs (e.g., Jian et al. 2006; Webb and Howard 2012), but the ambient solar wind structure to which CMEs are launched is also important. For example, the occurrence of the highest average ICME speeds in the declining activity phase may be related to the prevalence of fast solar wind from the polar coronal holes (e.g., Mursula et al. 2015) increasing the background speed, and consequently the average ICME speeds (e.g., Gopalswamy et al. 2000; Kilpua et al. 2012; Richardson and Cane 2012b; Richardson 2013). Figure 22 also shows that ICMEs and sheaths had, on average, weaker magnetic fields and they were less frequent during Solar Cycle 24 than during the previous, clearly stronger, cycle.

A particularly important solar cycle trend is the variation in the magnetic complexity of ICMEs. Near solar minimum almost all ICMEs detected near the ecliptic at 1 AU are well-defined MCs, while towards solar maximum the fraction of MCs decreases dramatically to only about 10–20% (e.g., Richardson and Cane 2004; Huttunen et al. 2005; Jian et al. 2006; Riley et al. 2006; Riley and Richardson 2013). This trend is consistent with a considerably higher fraction of ICMEs being crossed near the apex at solar minimum than at solar maximum (Jian et al. 2006). At solar minimum CMEs originate predominantly from low latitude sources (e.g., Hundhausen et al. 1984) and the high-latitude eruptions deflect towards the ecliptic as discussed above. In addition, when the Sun is active, the likelihood of interactions between CMEs increases and CMEs may merge so that individual flux rope characteristics cannot be discerned anymore (see the following sections). It is also possible that near solar minimum CMEs have intrinsically simpler structure than near solar maximum (e.g., see Sheeley et al. 1999, and discussion in Kilpua et al. 2013b.

**Fig. 24:**
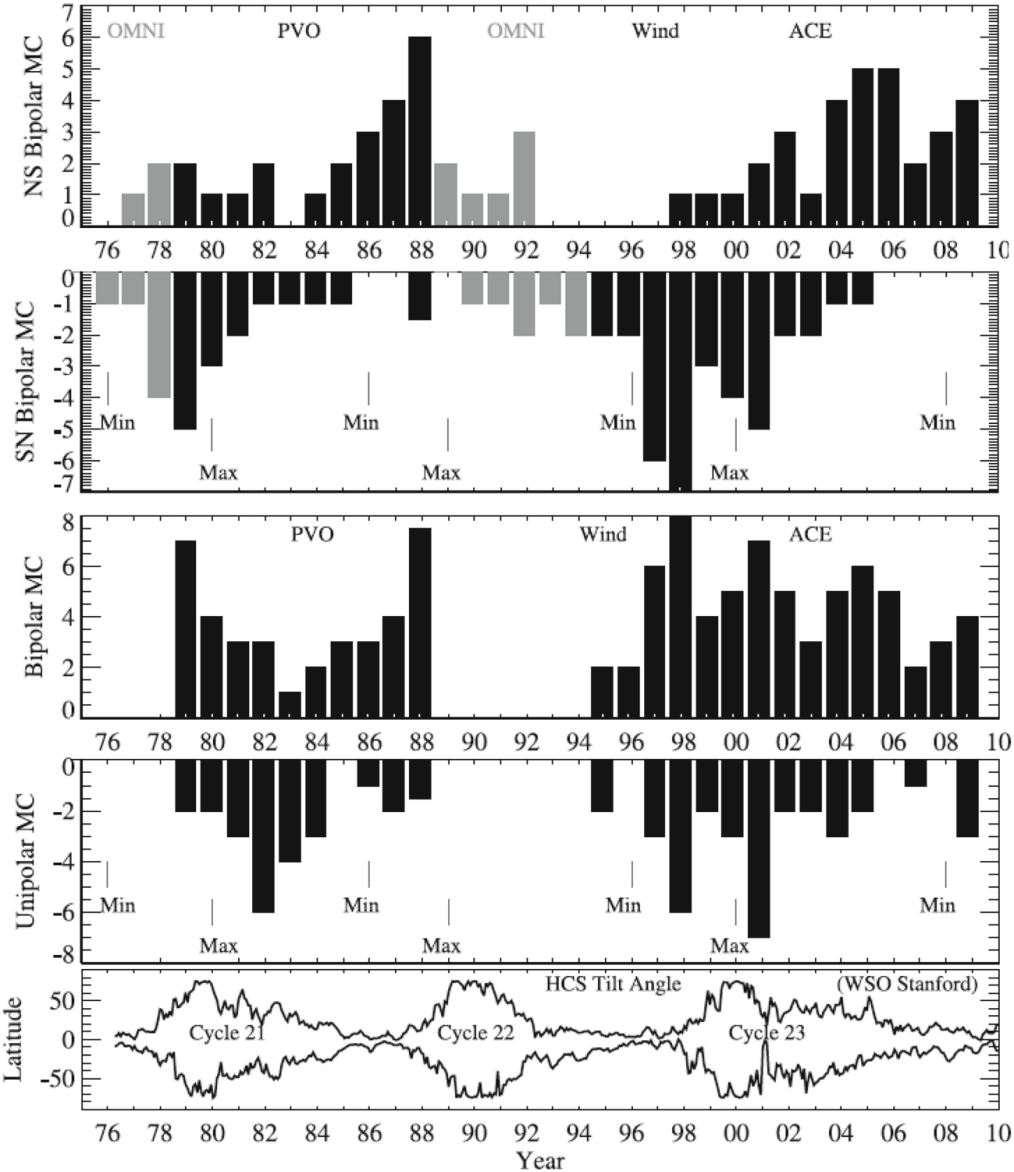
Solar cycle variations of NS- and SN-type bipolar magnetic clouds, and bipolar (S and N types combined) and unipolar (SN and NS types combined) magnetic clouds in the near-Earth solar wind. The four first panels show the annual counts and the fifth panel shows the N and S tilt angle of the heliospheric current sheet. The flux rope types are illustrated in Fig. 5. In this figure the letters refer only to the north-south magnetic field component (e.g., N can present both ENW and WNE type flux ropes). Image reproduced by permission from Li et al. (2011), copyright by Springer, where the third panel was erroneously labeled as bipolar, while it clearly shows the northward unipolar clouds

Figure 24 from Li et al. (2011) shows how the annual counts of bipolar and unipolar MCs vary over three solar cycles. The last panel in the figure shows the northern and southern tilt angle of the heliospheric current sheet, which follows the solar activity cycle (tilt is small near solar minimum and increases towards the maximum). For unipolar clouds the variations are mostly random, but bipolar clouds have a clear solar cycle trend. From the late declining phase of *odd* numbered solar cycles to the next rising phase, MCs with north-to-south (NS) field rotation are clearly more abundant, while during the corresponding phases of *even* numbered cycles south-to-north (SN) rotation dominates. At solar maximum when the global solar field changes sign, both SN and NS polarities are observed approximately at the same frequency. Hence, one can conclude that the Sun’s 22-year magnetic (Hale) cycle controls the type of flux ropes that are launched from the Sun (see also e.g., Mulligan et al. 1998; Bothmer and Schwenn 1998; Huttunen et al. 2005). The relative number of right- and left-handed clouds (not shown in Fig. 24 remains roughly the same at all times, in agreement with the general “hemispheric helicity rule” (e.g., Bothmer and Schwenn 1998; Li et al. 2011).

As is clear from Fig. 24 observations from a single observation point provide rather limited statistics. Li et al. (2014) showed that the annual number and polarity of MCs varied significantly between L1 and at two STEREO spacecraft, emphasizing the importance of having wider heliospheric coverage. They also found excursions from the otherwise clear solar cycle trend for the polarity of bipolar MCs for Solar Cycle 24.

### ICMEs at various heliospheric distances

So far in this review we have covered almost solely observations made at 1 AU where continuous solar wind observations have been available for about two solar cycles. A number of spacecraft have also probed the solar wind at different heliospheric distances, including Helios 1 and 2 (0.3–1 AU), Ulysses (1–5.4 AU), and the Voyagers (up to the heliopause). In addition, planetary missions, such as Pioneer Venus Orbiter, Cassini, MESSENGER, MAVEN and Venus Express, have provided occasional ICME observations, but typically with limited plasma and compositional/charge state information. Solar Orbiter (expected launch in 2019) and Parker Solar Probe (expected launch in 2018) will provide future observations at much closer distances from the Sun. Solar Orbiter’s perihelion will be at 0.28 AU, i.e., well inside Mercury’s orbit, while Parker Solar Probe will plunge as close as 10 solar radii from the Sun.

The identification of ICMEs becomes more problematic with increasing heliospheric distance. As CMEs propagate away from the Sun their magnetic field becomes weaker and they attain the pressure balance with the surrounding solar wind. For example, according to ideal MHD model for an expanding MC developed by Osherovich et al. (1993) the field enhancements with respect to the ambient solar wind can be seen up to 5–10 AU. As a consequence, at large heliospheric distances ICMEs are identified primarily from their plasma (mainly low temperature), and compositional/charge state signatures. Ulysses observations also show that counter-streaming electrons are found in ICMEs near 5 AU with approximately at the same frequency as at 1 AU. This suggests that the rate at which magnetic field lines open slows considerably as the ICME propagates further out into the heliosphere and that ICMEs remain connected to the Sun at such distances (Crooker et al. 2004).

**Fig. 25:**
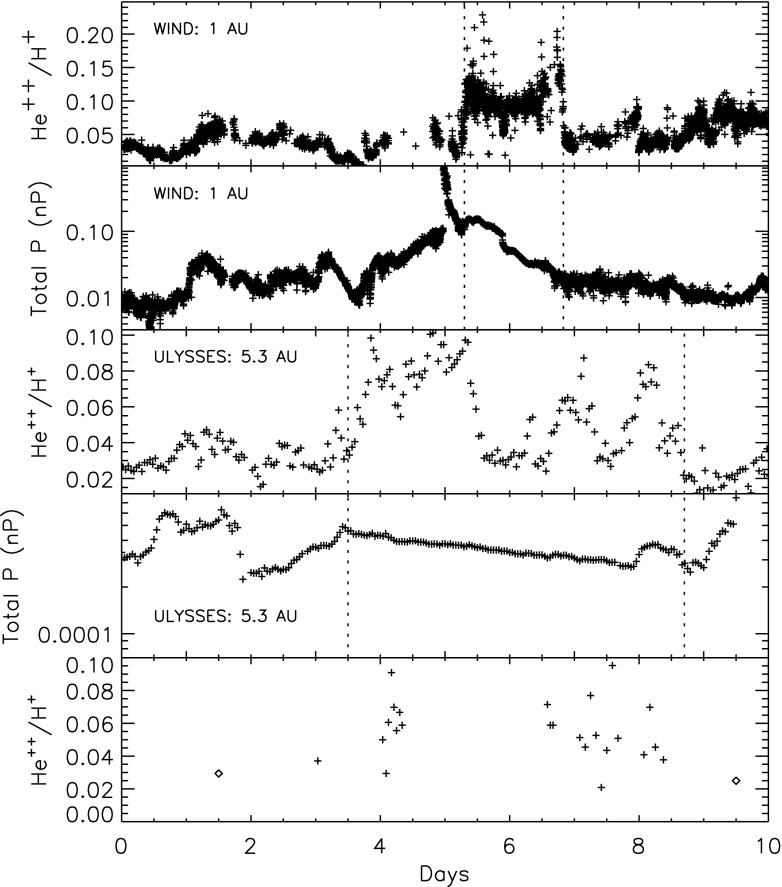
An ICME detected by Wind at 1 AU, Ulysses at 5.3 AU and Voyager 2 at 58 AU (bottom panel) using the total pressure (magnetic + plasma) perpendicular to the magnetic field (not available from Voyager 2) and alpha to proton ratio (He$^{++}/$H$^+$). The data from different spacecraft are time-shifted to align the ICMEs. Image reproduced by permission from Richardson et al. (2006); copyright by COSPAR (see also references therein)

A few ICMEs have even been identified at very large heliospheric distances ($> 50$ AU) from the Voyager data (e.g., Richardson et al. 2006, and references therein). Figure 25 shows an ICME that erupted from the Sun on 23 September 1998 and that was detected first near the Earth by Wind, then by Ulysses at the distance of 5.3 AU and finally by Voyager 2 as far as 58 AU from the Sun. At Voyager, the ICME was identified based on the enhanced helium to proton ratio. In a recent study, Witasse et al. (2017) tracked a CME that erupted from the Sun on October 2014 through the heliosphere using observations by several spacecraft. The CME was first detected by STEREO-A near 1 AU, then at Mars at 1.4 AU by Mars Express, Mars Odyssey and MAVEN, followed by Rosetta at 3.1 AU and Cassini at 9.9 AU, and finally by New Horizons at 32 AU when it was heading to Pluto. The authors also found indications of this ICME after over 1 year from its launch from the Voyager 2 data when the spacecraft was passing through the heliosheath at the distance of 111 AU from the Sun. A decrease in galactic cosmic rays was a key identifying ICME signature at Voyager 2. Forbush decreases were detected by Mars Odyssey and Mars Science Laboratory, and by Rosetta and Cassini.

**Fig. 26:**
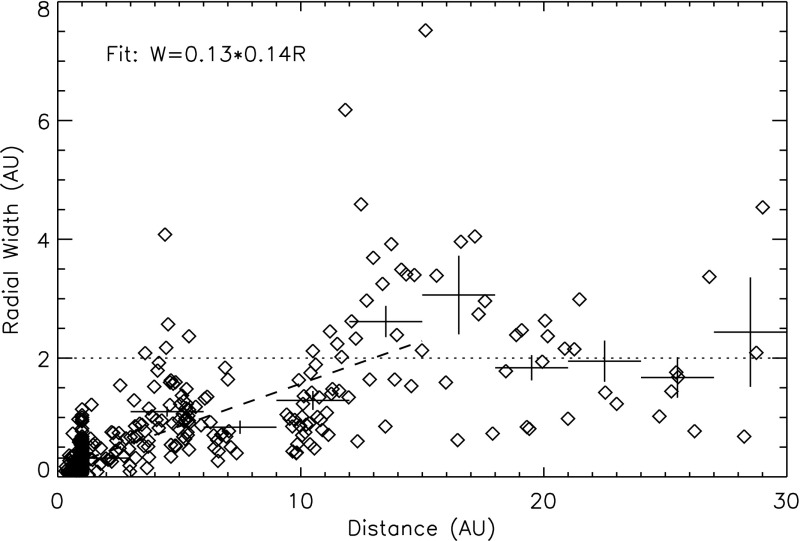
The width of ICMEs as a function of the heliospheric distance *R* from the Sun. Diamonds show the radial widths of ICMEs that were combined by Richardson et al. (2006) using previously published ICME lists. The widths averaged over 3 AU are shown by horizontal bars and the vertical bars give the error of the mean. The dashed line gives the best linear fit to observations inside 15 AU where ICMEs generally still expand. Image reproduced by permission from Richardson et al. (2006), copyright by COSPAR

In their review paper, Richardson et al. (2006) combined results from several previous studies to investigate how ICME radial widths and properties change in the inner and outer heliosphere (see also Table 1 from Forsyth et al. 2006). The studies were based on observations from Helios 1 and 2, Wind, ACE, Ulysses, Pioneer 10 and Voyager covering distances between 0.3 and 30 AU. As we discussed in Sect. 2, ICMEs expand after they are launched from the Sun, and as consequence their radial widths increase significantly. The expansion in interplanetary space is primarily driven by decreasing total solar wind pressure with increasing distance from the Sun, although closer to the Sun the internal over-pressure within the CME can also be an important contributor (e.g., von Steiger and Richardson 2006; Démoulin and Dasso 2009). Figure 26 shows that the average radial width (calculated from the best fit line) increases from 0.17 AU at 0.3 AU to 2.2 AU at 15 AU. After this, the radial widths stay more or less constant reflecting that expansion ceases and ICMEs reach the pressure balance with the ambient solar wind.

**Fig. 27:**
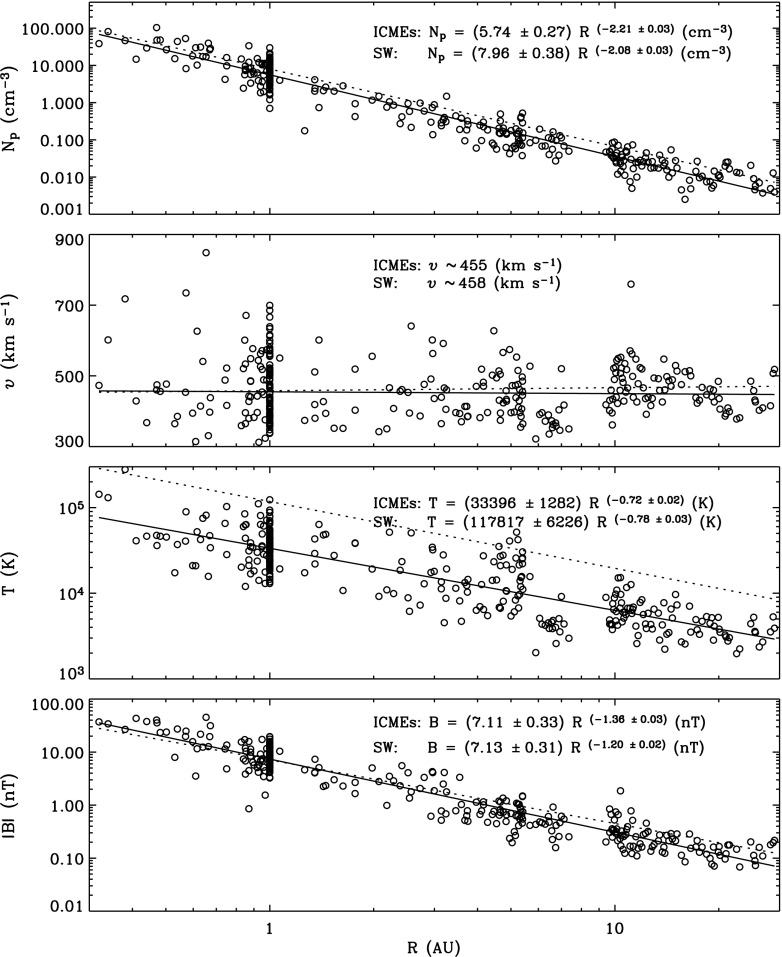
The panels show from top to bottom the average solar wind density, speed, temperature and magnetic field magnitude as a function of heliospheric distance (*R*) from the Sun. Also shown are fits to the (solid) ICME data and fits to the (dashed) ambient, i.e., non-ICME, solar wind data. The data points combine measurements from Helios 1 and 2, Wind, ACE, Ulysses and Voyager spacecraft. Image reproduced by permission from Richardson et al. (2006), copyright by COSPAR; see also the references therein

The changes in the average temperature, density and magnetic field in ICMEs as well as in the quiet solar wind are shown in Fig. 27. When ICMEs propagate away from the Sun and expand, their magnetic field, temperature and density decrease. While magnetic field and density in ICMEs decrease faster than in the ambient solar wind, the temperature decreases more slowly (see the *R* dependencies from Fig. 27). Similar to the ambient solar wind speed, the average ICME speeds (data not shown) remain roughly constant throughout the heliosphere. These trends have also been confirmed by radially aligned spacecraft that have encountered the same ICME (e.g., see Forsyth et al. 2006; Good and Forsyth 2016, and references therein).

**Fig. 28:**
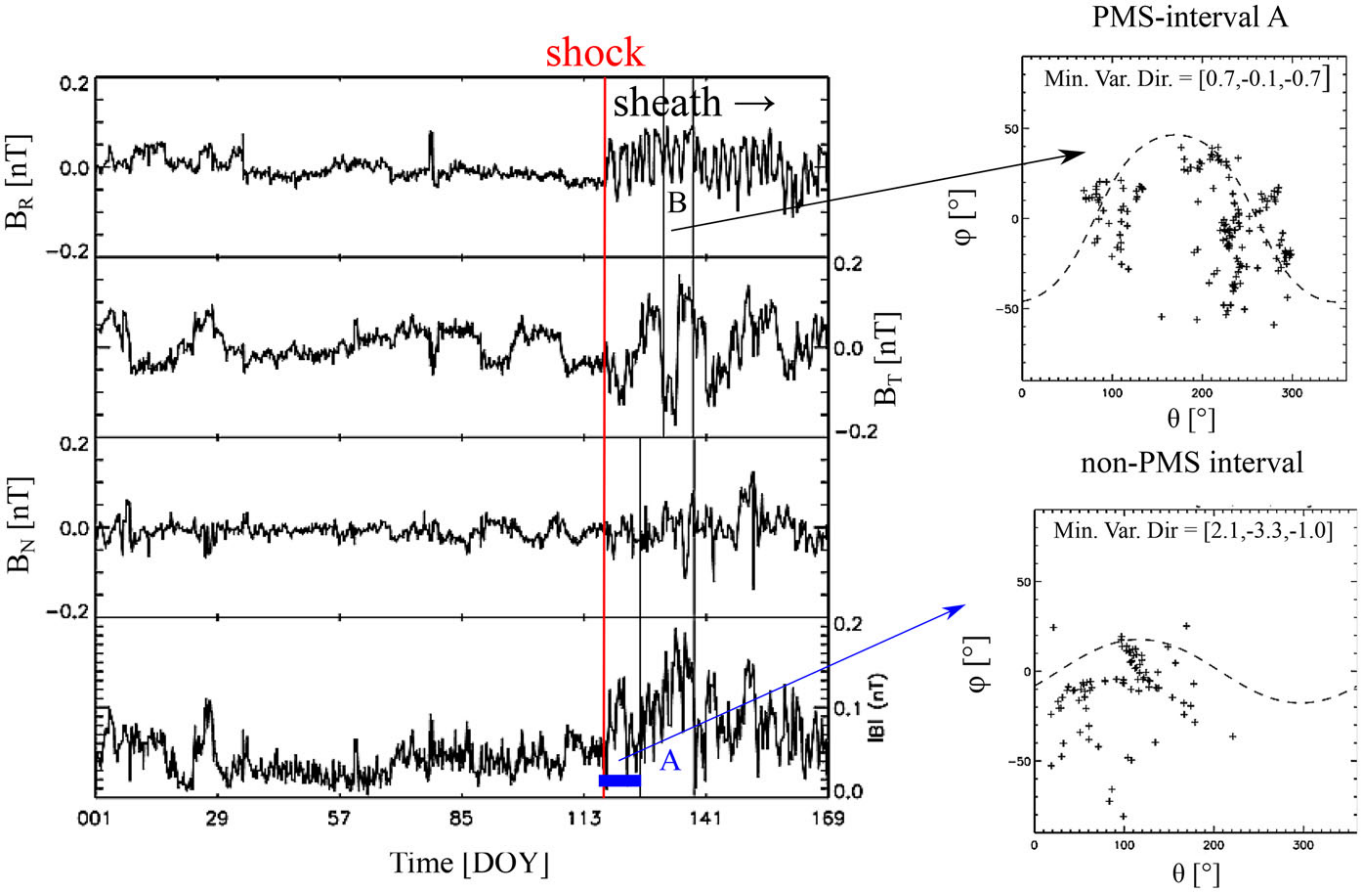
(Left) Voyager 2 magnetic field data measured within the ICME sheath region at the heliospheric distance of 73.2 AU from the Sun. The panels give from top to bottom the magnetic field components in the RTN coordinates and the magnetic field magnitude. Intervals A and B are planar structures, while the horizontal blue bar indicates the interval where PMSs were not found. (Right) The $\phi$–$\theta$ diagram (where $\phi$ and $\theta$ are the IMF longitude and latitude). The plane perpendicular to the PMS normal is represented by the dashed curve. PMSs are identified as the periods when magnetic field vectors are distributed in the close proximity of the curve presented. The top panel shows the interval A when the PMSs were found, and the bottom features the non-PMS interval. Images adapted from Intriligator et al. (2008), copyright by AGU

The ICME sheaths also evolve with increasing heliospheric distance. When ICMEs expand, their sheaths are relatively thick but when the radial expansions ceases, the relative sheath thickness decreases (e.g., Richardson 2011). The lateral expansion, however, has not been quantified at large heliospheric distances due to the lack of consistent measurements at high latitudes. The draping of the magnetic field increases the further an ICME propagates and expands through more slowly moving solar wind (e.g., McComas et al. 1988). Intriligator et al. (2008) analysed planar magnetic structures (PMS) throughout the heliosphere in a sheath of the Halloween 2003 CME. As discussed in Sect. 4.3, PMSs are intervals where the magnetic field vectors remain parallel to a fixed plane over an extended time period. The authors combined measurements from ACE (1 AU), Ulysses (5.2 AU), Cassini (8.7 AU), Voyager 1 (73.2 AU) and Voyager 2 (93 AU). The analysis showed that PMSs became more clearly defined as the ICME propagated outward in the heliosphere and their planes became more normal to the radial direction. Figure 28 shows the magnetic field data from Voyager 2 and examples of the identified planar and non-planar intervals.

### ICMEs observed off-the ecliptic

The majority of observations of heliospheric plasma and IMF come from the near-ecliptic spacecraft. Ulysses is so far the only spacecraft that has consistently probed the solar wind at large heliographic latitudes. Between 1992 and 2009 Ulysses made three high-latitude scans reaching the maximum latitude of about $80^{\circ }$ both south and north of the ecliptic. The first and third polar scans in 1992–1997 and 2004–2009 covered declining and minimum solar activity phases, while the second polar pass in 1998–2003 coincided with increasing and maximum solar activity phases. The future higher-latitude observations will be provided by Solar Orbiter that will reach latitudes up to $34^{\circ }$.

During the Ulysses first and third polar scans ICMEs were confined to lower latitudes, whereas during the second scan that coincided with high solar activity ICMEs were identified up to the maximum latitude reached by Ulysses (e.g., Gazis et al. 2006; von Steiger and Richardson 2006; Richardson 2014). As expected, at lower heliographic latitudes ($< 40^{\circ }$) the ICME rate from Ulysses matches approximately with the ICME rate at 1 AU for all investigated solar cycle phases (Richardson 2014, and the references therein). The high-latitude ($> 40^{\circ }$) ICME rate increased from practically zero close to solar minimum to 0.8 ICMEs per Carrington rotation at solar maximum (Richardson 2014). Even though the high-latitude ICME rate clearly increased with the increasing solar activity, it was still below the low latitude ICME rate that was reported to be 0.9–1.5 ICMEs/Carrington rotation by Richardson (2014) at solar maximum.

As discussed, e.g., by Gazis et al. (2006) and Richardson (2014) the above results can be explained by most CMEs originating from mid-latitude active regions during high solar activity, while during solar minimum the main CME source is the streamer belt that is confined at low latitudes. In addition, as discussed in Sect. 5.1, at solar minimum high-latitude CMEs from polar crown filaments tend to deflect towards lower latitudes. The compositional and charge state signatures of ICMEs also reflect the variations in dominant source regions at different latitudes. Richardson (2014) reported that elevated ion charge states are most common below $40^{\circ }$, but the probability decreases again for ecliptic ICMEs. The authors suggest that ICMEs with higher charge states are those whose latitudes match best with the mid-latitude active regions. Plasma in active region CMEs is expected to have been heated more strongly in the eruption process (often including flares) than in CMEs that originate from the streamer belt or from polar crown filaments. The ICMEs seen at the highest latitudes can be part of ICMEs that originate from mid-latitude active regions and then expand towards higher latitudes. For example, Reisenfeld et al. (2003) studied two ICMEs observed by Ulysses both in the ecliptic and at latitudes exceeding $70^{\circ }$.

The ICME speed also depends on the heliospheric latitude. While during the first Ulysses fast latitude scan high-latitude ICMEs were absent, the lower latitude ICMEs (up to about $40^{\circ }$) showed increasing minimum speed with the heliospheric latitude (Richardson 2014). During the next scan that coincided with high solar activity this trend was not observed. This observed increase in the minimum ICME speed with latitude during solar minimum is presumably due to the increasing ambient solar wind speed due to fast streams from polar coronal holes. At solar maximum there is clearly less latitudinal variation in the solar wind speed (e.g., McComas et al. 2003). Gosling et al. (1995) also noted that nine ICMEs they studied during the Ulysses first fast latitude scan at latitudes between $31^{\circ }$ and $61^{\circ }$ had the average speed of 740 km s$^{-1}$, clearly higher than the average speed $\sim 400$ km s$^{-1}$ near the ecliptic (see e.g., Fig. 22).

Richardson (2014) also reported that the fraction of MCs from all ICMEs increased somewhat with the increasing heliospheric latitude from the ecliptic. This is consistent with the assumption that non-MC ICMEs are cases where the core of the flux rope is not intercepted. As discussed above, near solar maximum when the ICME rate is highest, the active regions are clustered at mid-latitudes.

Gosling et al. (1994) studied a forward–reverse shock pair associated with a Ulysses ICME at the latitude of $32.5^{\circ }$. The authors concluded that the CME expansion due to high initial internal plasma and magnetic field pressures was the main driver of the shock pair and they coined the term “over-expanding ICME”. A later study by Gosling et al. (1995) analyzed a case where the same CME was observed by IMP-8 near the ecliptic and by Ulysses at a high heliographic latitude at 3.5 AU. The near-ecliptic observations showed only a fast forward shock leading the ICME, while Ulysses observed a reverse–forward shock pair. The observations during the second fast latitude scan by Ulysses confirmed that over-expanding ICMEs are observed also during solar maximum, but are relatively less frequent than near solar minimum (e.g., Reisenfeld et al. 2003). The over-expanding ICMEs tend to have large radial widths. For example, the ICME event studied by Richardson et al. (2002) (see also von Steiger and Richardson 2006) had a diameter of 0.6 AU at the location of Wind, much larger than the average ICME width at these distances (see Table 1).

### Observations of ICME interactions in the heliosphere

Interactions of ICMEs with each other or with the ambient solar wind can considerably change their internal structure and affect their kinematics. In addition, the interfaces where the interaction takes place are particularly interesting as they provide direct observations of fundamental plasma physical processes, such as turbulence and magnetic reconnection. For a comprehensive review on CME interactions during their propagation see, e.g., Manchester et al. (2017).

As CMEs arise from closed field line regions, the majority of ICMEs in the ecliptic are found either in the slow solar wind or at the leading edges of fast solar wind streams (e.g., Kilpua et al. 2015b; Rodriguez et al. 2016; Benacquista et al. 2017). A trailing fast stream can significantly compress plasma and the magnetic field in the rear of the ICME (e.g., Fenrich and Luhmann 1998; Kilpua et al. 2012), enhancing also its ability to drive space weather disturbances. Interacting ICMEs are generally more difficult to model due to non-force-free effects and, if merged significantly, their lack of a clear field rotation. In particular at increasing heliospheric distances ICMEs get more and more engulfed into fast streams and they become part of larger-scale heliospheric structures called compound streams (e.g., Burlaga et al. 1987). On the other hand, an ICME may also collide with a slower and higher density heliospheric plasma sheet ahead, which may compress its front part (see e.g., Kataoka et al. 2015).

**Fig. 29:**
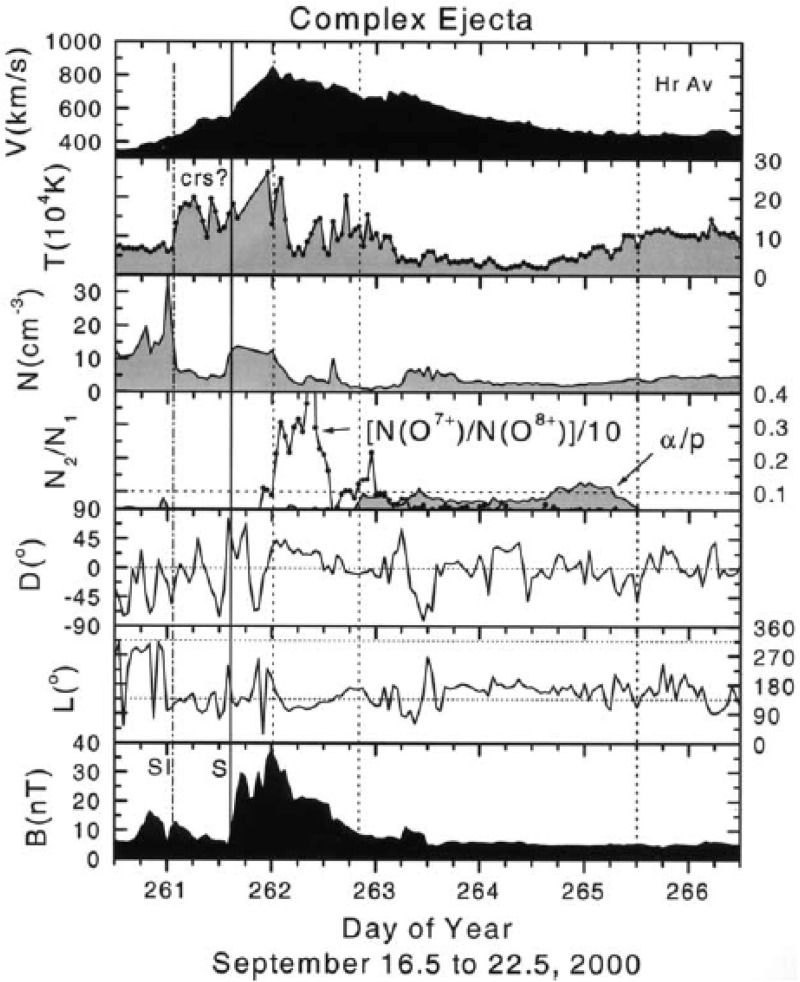
A complex ejecta. The panels show from top to bottom: solar wind speed, temperature, density, alpha to proton and O$^{+8}/$O$^{+7}$ ratio, elevation and azimuth angle of the magnetic field, and magnetic field magnitude. Image reproduced by permission from Burlaga et al. (2002), copyright by AGU

Interactions between multiple ICMEs can lead to particularly complex interplanetary structures. Sometimes successive ICMEs follow each other without significant interference, but in some cases they merge into “complex ejecta” where characteristics of individual ICMEs are lost (e.g., Burlaga et al. 2002; Lugaz et al. 2005). As stated by Burlaga et al. (2002) merging is a nonlinear and irreversible process, where memory of the source conditions is lost. The merging takes place due to magnetic reconnection between the consecutive CMEs (see Lugaz et al. 2017, and references therein). Figure 29 shows an example of a complex ejecta observed during September 16–22, 2000. It was related to four CMEs launched from the same active region within less than a day. A characteristic property of complex ejecta is that their speed profile shows first a rapid rise to a maximum followed by a much slower decline. As noted by Burlaga et al. (2002) such a speed profile resembles a fast solar wind stream.

Particularly strong magnetic fields in interplanetary space can result when interacting ICMEs do not merge significantly. An example is a complex ICME on 23 July 2012 observed in-situ at STEREO-A. It resulted from two powerful CMEs that interacted very close to the Sun and had one of the highest magnetic fields (109 nT) ever measured near the Earth orbit (e.g., Liu et al. 2014). The factors that control the CME–CME interactions are not yet well understood but the details should depend, e.g., on their relative launch direction, timing, speed and the magnetic field orientation (e.g., Lugaz et al. 2013). An ICME shock that is overtaking the preceding ICME can additionally compress the magnetic field within the ICME (Burlaga 1991; Lepping et al. 1997).

**Fig. 30:**
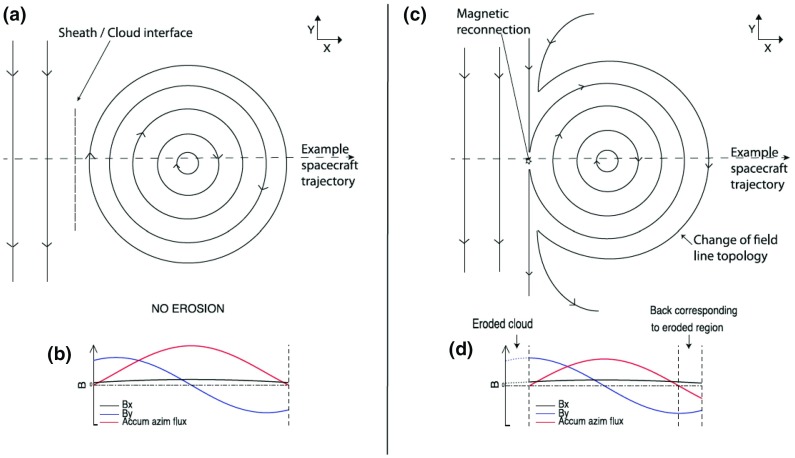
Illustration of the magnetic configuration of (left) a non-eroded magnetic cloud and (right) an eroded magnetic cloud. The red curve shows the variations of the accumulated azimuthal flux and the blue and black lines the variations in the field components. The coordinate system is the MC frame (see definition, e.g., in Dasso et al. 2005). Image reproduced by permission from Ruffenach et al. (2012), copyright by AGU

Even interactions with the quiet solar wind may have a significant impact on ICME properties. In particular, magnetic reconnection is a common phenomenon at the flux rope boundaries and can erode a significant fraction of the cloud’s magnetic flux and affect its geoeffectivity (Lavraud et al. 2014). Studies of azimuthal flux imbalance (see the method in Dasso et al. 2007) suggest that near the Earth’s orbit the average flux erosion could be as much as 40% of the original flux (Ruffenach et al. 2015). The erosion may increasingly peel away the magnetic flux as the ICME propagates further out in the heliosphere (e.g., McComas et al. 1988). Figure 30 shows how the erosion leads to the asymmetric profile of the azimuthal flux (red curve) and in the rotation profile of the magnetic field direction. Whether or not, and how fast, reconnection takes place, depends largely on the relative orientation of the magnetic field and speed at the boundaries and the thickness of the resulting current sheet (e.g., Schmidt and Cargill 2003). The gradual peeling of the magnetic flux will decrease the radial width of the MC and it has also been proposed as a possible explanation why MCs often occupy only a portion of the more extended ICME structure (see Sect. 2.2).

## Space weather driven by ICMEs and sheaths

Space weather refers to the changing conditions on the Sun, in the solar wind and in planetary space environments, including the ionosphere and the upper atmosphere, that can affect the performance and reliability of man-made systems on ground or in space, and endanger human life or health. Space weather has become increasingly important due to the growing dependence of the society on space infrastructure and systems sensitive to electromagnetic disturbances and energetic particle radiation (e.g., Baker and Lanzerotti 2016). ICMEs and their shocks and sheaths are the most important drivers of hazardous space weather, as they both interact directly with the planetary space environments and accelerate particles to high energies. This section starts with discussing ICMEs and sheaths as drivers of geomagnetic activity. We cover separately the storms in the magnetosphere and auroral zone activity. Thereafter we briefly review the effects of ICMEs and sheaths on radiation belts and the atmosphere. We conclude with a short discussion on ICMEs and exoplanets. A particular aspect on which we are focusing in this part of the review is how different properties of ICMEs and sheaths (see Sect. 4.2) affect the resulting space weather disturbances.

### ICMEs/sheaths as drivers of storms in the magnetosphere

Magnetic storms are defined as long-lasting (hours to days and even weeks) disturbances in the horizontal component of the geomagnetic field (e.g., Gonzalez et al. 1994; Pulkkinen 2007). The primary driver of such disturbances is the large-scale advection, often called in this context convection, in the magnetosphere, first suggested by Dungey (1961), that energizes and transports particles into the near-Earth magnetosphere. In the Dungey cycle magnetic reconnection at the subsolar magnetopause opens the closed terrestrial field. The newly-opened field lines are carried antisunward with the solar wind and the magnetic flux accumulates in the tail lobes. At some point this leads to reconnection in the distant magnetotail, closing the flux again. The newly-closed flux is advected back to the dayside magnetosphere. This leads to buildup of a strong ring current created primarily by the drift of electrons and ions in the energy range of few tens of keVs to several hundreds of keVs. The enhancement of the ring current electron flux in the inner magnetosphere is a hazard to the high-altitude satellites, as these electrons can cause spacecraft surface charging and associated anomalies (e.g., Garrett 1981; Lanzerotti et al. 1998; Ganushkina et al. 2015). The 1-h Dst index, which is constructed from low-latitude magnetometer observations, gives a rough estimate of the equatorial ring current intensity. Another widely used storm-time index is the 3-h Kp-index, based on mid-latitude magnetograms (for a description of geomagnetic indices see, e.g., Mayaud 1980).

The reconnection at the dayside magnetosphere occurs when the solar wind and terrestrial magnetic fields are anti-parallel, or at least have anti-parallel components. To drive a storm the IMF has to have a sufficiently large and long-lasting southward magnetic field component (e.g., Dungey 1961; Gonzalez et al. 1994). For example, according to Gonzalez et al. (1994), for a moderate Dst storm (Dst $< - 50$ nT) $B_Z$ in the solar wind has to be below $-5$ nT for at least 2 h, and for an intense storm (Dst $< - 100$ nT) the requirement is $B_Z < -10$ nT for at least 3 h. When present, high solar wind speed further intensifies the driving by increasing the dawn-to-dusk solar wind electric field (approximated usually with $E_Y = V B_S$, where *V* is the solar wind speed, and $B_S$ the magnitude of the southward IMF component) imposed on the magnetosphere.

The role of ICMEs in driving magnetic storms has been a long-debated topic. By the 1850s, it was evident that some solar flares were connected with geomagnetic activity (e.g., Carrington 1859) around a day or more later, though the link between the two phenomena was unclear. As we discussed in the introduction, the plasma clouds originating from the Sun were suggested as the causes of geomagnetic disturbances even before space era by various authors. Even after the CMEs were finally found, the dispute whether geomagnetic storms were driven by flares or by CMEs lasted until conclusive evidence in favour of CMEs was gathered, as summarized in the landmark paper “The Solar Flare Myth” by Gosling (1993). The International Solar Terrestrial Physics (ISTP) Program was created in the early 1990s to study the Sun–Earth connection with multiple spacecraft focusing on the Sun, the solar wind, and different parts of the magnetosphere (Acuña et al. 1995). This program was successful indeed in generating an extensive set of case studies demonstrating the sequence of events from the solar surface to the magnetosphere and ionosphere (e.g., Fox et al. 1998, and the other papers in the same topical issue of *Geophys. Res. Lett.*).

**Fig. 31:**
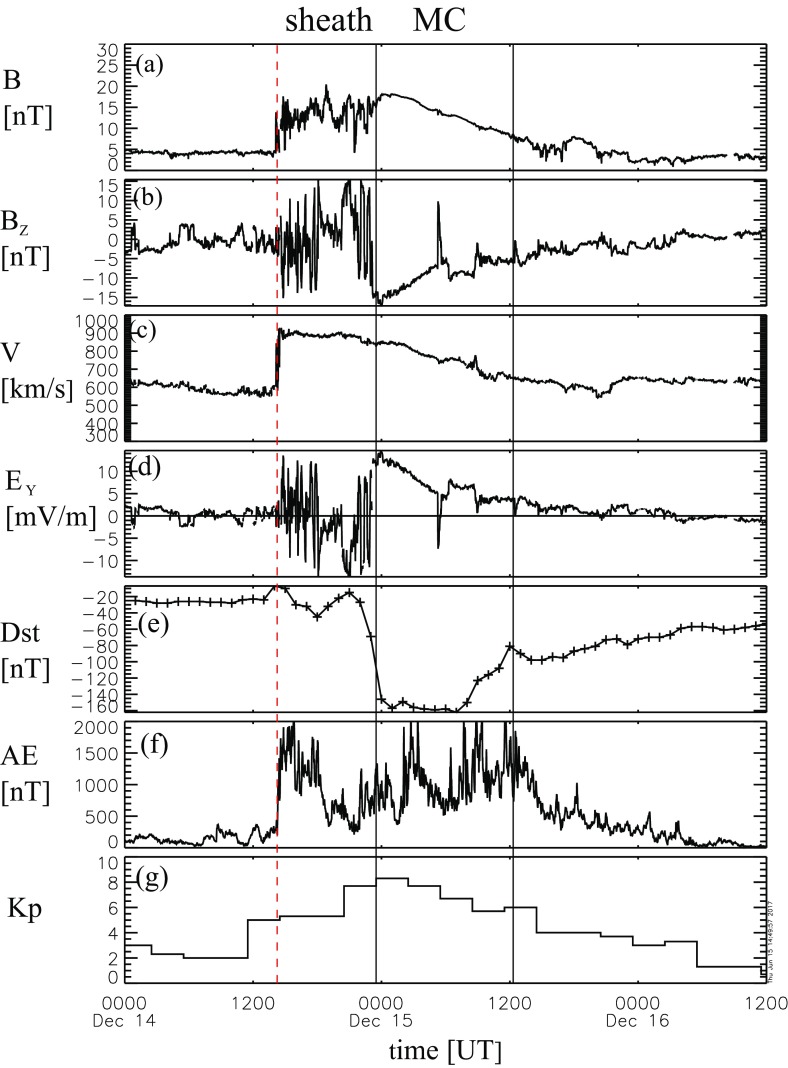
Example of a sheath and a magnetic cloud driving geomagnetic activity on December 14–16, 2006. The panels show from top to bottom: *a* magnetic field magnitude, *b* magnetic field north-south component (in GSM coordinates), *c* solar wind speed, *d* solar wind dawn-dusk electric field, *e* Dst, *f* AE (see Sect. 6.2) and *g* Kp. The data are 1-min OMNI data, Dst is 1-h average and Kp 3-h average. The dashed red line marks the shock and the pair of solid lines marks the MC interval

The strong and slowly rotating magnetic field within an ICME (particularly in the flux rope part), when oriented anti-parallel to the geomagnetic field on the dayside magnetopause, intensifies the large-scale magnetospheric convection and, consequently, the ring current. Figure 31 shows an example of a magnetic storm driven by a sheath and a MC. The sheath and MC are clearly distinguished by the level of fluctuations in the magnetic field. The sheath has large-amplitude variations in the $B_Z$ component while the field in the MC is distinctly smoother and $B_Z$ stays southward during the whole passage of the MC. The Dst index starts to decrease during the passage of the sheath region, but the main decrease in this event occurs during the leading half of the MC, where the strongest and persistent southward field is present. The storm then begins to recover when the southward field declines further into the MC.

**Fig. 32:**
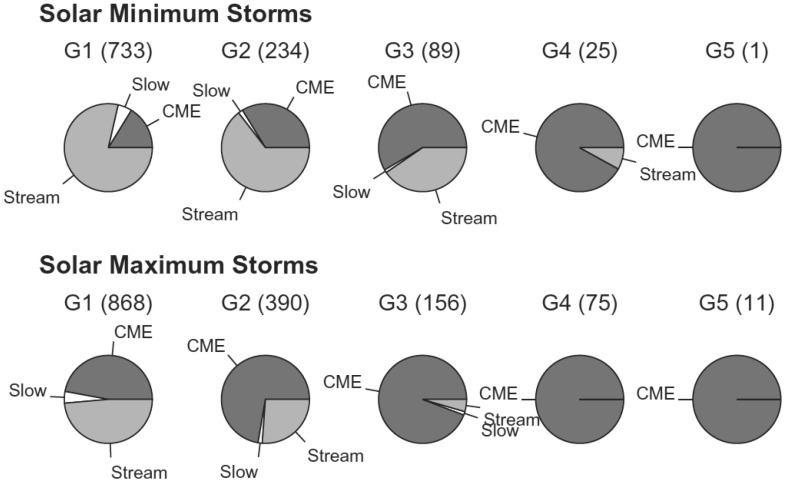
Drivers of geomagnetic storms around solar minimum and solar maximum accumulated over four solar cycles (1964–2011). The geomagnetic storm definition follows NOAA storm scale (http://www.swpc.noaa.gov/noaa-scales-explanation). Image reproduced by permission from Richardson and Cane (2012b), copyright by the authors

We now know that ICMEs are the prime source of intense magnetic storms (e.g., Gosling et al. 1991; Webb et al. 2000; Huttunen and Koskinen 2004; Zhang et al. 2007; Richardson and Cane 2012b) and hence the occurrence rates of both track the solar activity cycle (e.g., Richardson and Cane 2012b). Figure 32 shows the drivers of geomagnetic storms according to the NOAA storm scale classification from minor (the NOAA storm scale class G1, corresponding to Kp $=$ 5) to extreme (G5, corresponding to Kp $=$ 9) storms over four solar cycles for solar minimum and maximum periods. ICMEs drive nearly all storms in the three strongest storm categories, but are also important drivers of moderate and weak storms particularly near solar maximum. Note that this analysis assigns the drivers to three categories of slow and fast solar wind, and “CME-associated” structures which include the sheath and ICME/magnetic cloud.

According to Figs. 18 and 19, sheaths have many properties that enhance solar wind–magnetosphere coupling efficiency (see the detailed discussion of this topic in Kilpua et al. 2017); high dynamic pressure (e.g., Palmroth et al. 2007; Myllys et al. 2016), enhanced ULF wave power causing enhanced viscous interactions at the magnetopause (e.g., Borovsky and Funsten 2003) and high Alfvén Mach numbers causing stronger compression of the plasma and field at the bow shock and the cross polar cap potential saturating at larger driving electric fields than during low Alfvén Mach number conditions (e.g., Borovsky and Birn 2014; Myllys et al. 2016). Large-scale fluctuations in the IMF north-south component could also build the ring current up more efficiently than a smoother driver of similar magnitude due to periodic trapping of the particles in the ring current region (e.g., Kamide et al. 1997). However, as the ICME encounters last, on average, longer than the sheath encounters, the total energy input into the magnetosphere is typically larger during the ICME than during the sheath region. In addition, the importance of ICME versus sheath or their combination as storm drivers varies with solar cycle (e.g., Zhang et al. 2004; Echer et al. 2008). Sheaths are the most geoeffective structures in the ascending phase of the solar cycle and near solar maximum, while the relative geoeffectivity of the ICMEs increases in the declining phase of the solar cycle. This could be partly related to the fraction of MCs from all ICMEs being considerably larger near weaker solar activity times than at solar maximum, see Sect. 2.2.

**Fig. 33:**
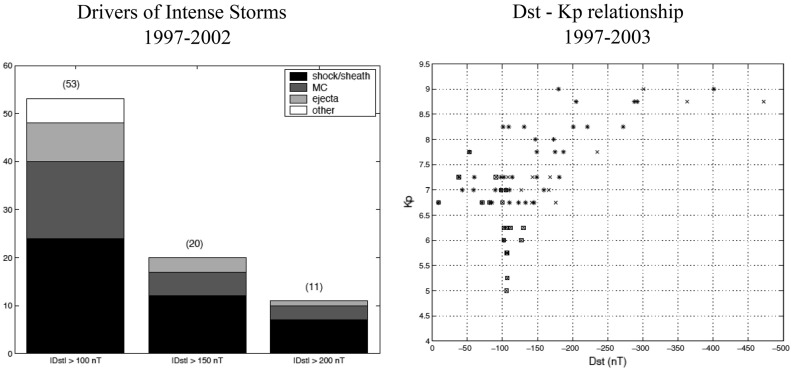
(Left) Drivers of magnetic storms for three different Dst limits. (Right) The minimum Dst versus the maximum Kp for intense storms (Kp $\ge$ 7 and/or Dst $< -100$ nT) for 1997–2003. Note that in several cases the storm is defined as “intense” only by one of the used indices, while the other indicates more moderate activity (see discussion by Huttunen et al. 2002 and Huttunen and Koskinen 2004). Sheath storms are shown by asterisks and magnetic cloud storms by crosses. The rectangles show the cases that did not fulfill the Gonzalez et al. (1998) intense Kp criterion for an intense storm, i.e., the requirement that Kp has to be $\ge 6$ at least for three 3-h periods. Images reproduced by permission from [left] Huttunen and Koskinen (2004), copyright by the authors; [right] Koskinen and Huttunen (2006), copyright by Springer

When the storm driver is defined as the structure during which the peak of the geomagnetic index is reached, the sheath regions are equally, or even more, effective in driving the storms than the ICMEs. In particular, a significant number of ICME-related storms are pure sheath-induced storms (e.g., Tsurutani et al. 1988; Huttunen et al. 2002; Huttunen and Koskinen 2004). The left-hand part of Fig. 33 compares the efficiency of different drivers, shocks/sheaths, non-MC ICMEs (ejecta), and magnetic clouds, in driving the peak of the major magnetic storms as determined by the Dst index. Taken together, non-MC ICMEs and MCs account for about half of the intense storms (Dst $< -100$ nT), but for the strongest storms, the proportion of shock/sheath drivers increases.

In reality, the solar wind structures driving the storms are often complex and consist of multiple components. Most typical are storms where both the sheath and the ICME have a significant contribution, and storms that are driven by multiple interacting ICMEs. A significant fraction of ICMEs are also followed by a fast solar wind stream, which in particular can prolong the storm recovery phase. As we discussed in Sect. 5.4, geoeffectivity of an MC with north to south rotating field can be greatly enhanced if it is followed by a fast stream that compresses the southward field in in the ICME’s rear part (Fenrich and Luhmann 1998; Kilpua et al. 2012; Benacquista et al. 2017). We discussed in Sect. 5.4 that in some cases a shock of an ICME may propagate into the preceding ICME. A statistical analysis by Lugaz et al. (2015) indicates that 39% (19 out of 49) of shocks propagating inside ICMEs are associated with an intense geomagnetic storm. A general conclusion is that southward interplanetary field is required before storm activity commences, whether as a part of a smooth rotation or as a part of a strongly fluctuating sheath region. As sheaths and MCs are macro-scale structures, the timing of a storm can differ over one day depending on in which part of the structure(s) the southward fields are embedded.

### ICMEs/sheaths as drivers of auroral zone activity

**Fig. 34:**
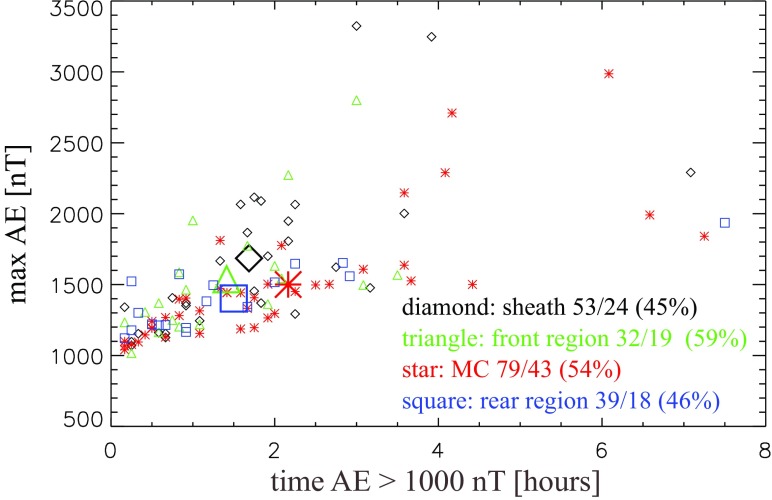
High-latitude response to sheaths (black diamonds), MCs (red stars), and the MC front (green triangles) and rear regions (blue squares). The data set consist of the same 79 events from 1996 to 2009 as analyzed in Kilpua et al. (2013b). The plot shows the maximum AE as a function of total time when AE $> 1000$ nT within each region. The large symbols show the averages for each region. The first number gives the total number of each region (note that not all MCs have sheaths, front or rear regions) and the second number the cases that had significant high-latitude response (AE $> 1000$ nT) (percentage also shown in parenthesis)

The auroral zone activity can be indexed using the auroral electrojet indices AL, AU, and AE determined from ground-based magnetometers in the auroral latitudes (e.g., Mayaud 1980). AL characterizes the westward current in the auroral zone ionosphere, AU the eastward current. AE is the difference AU–AL (note that AU is positive and AL negative). Figure 34 shows the AE response to the sheath, the MC, and the front and rear region of the MC (i.e., the regions that show ICME signatures, but that are not MCs; recall these definitions from Sect. 2.3). The horizontal axis shows the time in hours when AE index exceeds 1000 nT (indicating strong activity in the auroral region) and the vertical axis the peak AE value (when $> 1000$ nT) during the given structure. The figure shows that while all structures may lead to a significant high-latitude activity, MCs are the most typical drivers of the longest periods of intense AE activity. This is expected as their passages past the Earth last on average clearly longer than the passage of the sheaths, front or rear regions. In turn, sheaths, and also front regions, cause together with MCs the largest peak AE values. The rear regions are the least geoeffective structures as they have on average weaker magnetic field magnitudes than the other investigated structures (Kilpua et al. 2013b).

The strong response of auroral region current systems to some sheath passages is also shown in the left panel Fig. 33, i.e., the majority of storms with the largest Kp values are related to sheaths (auroral currents have a major contribution to the Kp index). Figure 31 also shows that during our example storm event the AE index had remarkably high values during the turbulent sheath region although Dst was not significantly depressed. Note that auroral activity continues at the similar level throughout the MC when the intense Dst storm occurs. The relatively strong response of auroral currents to sheaths is also evident from the right-hand part of Fig. 33 where it is shown that sheath-driven storms (asterisks) were generally associated with larger Kp values than ICME-driven storms (crosses).

The large-amplitude magnetic field fluctuations and the overall turbulent nature of the sheath regions can effectively enhance the Region 1 field-aligned currents coupling the magnetosphere and its outer boundary layers to the ionosphere, and thereby drive strong activity at auroral latitudes. Such fluctuations are also effective in triggering magnetospheric substorms, which drive auroral activity through the substorm current wedge associated with the magnetospheric reconfiguration process (e.g., Baker et al. 1996, and references therein). At times, the sheath encounters can cause “high-latitude storms” that are associated with strong auroral latitude activity even if the ring current or radiation belt signatures of a storm would be missing (Huttunen and Koskinen 2004).

Strong ionospheric currents associated with high AE cause space weather effects on ground. The space and time varying electric currents produce a varying magnetic field at the Earth’s surface, which in turn induces an electric field in the crust and upper mantle of the Earth. This electric field drives geomagnetically induced currents (GIC), which seek the highest conductivity paths, and thereby penetrate to long man-made conductors such as power transmission systems, communication cables, or natural gas pipelines in the high-latitude regions (e.g., Pulkkinen 2015). The voltage instabilities created by these currents can lead to damage to transformers connected to power grids, cause failures of connections in telecommunication cables, and result in corrosion in long pipelines if the currents flow between the pipeline and the insulating ground. Furthermore, the increased electron density and highly structured currents in the ionosphere can cause disturbance or loss of radio signals, and modify the signal from global navigation satellite systems such as the GPS and GALILEO fleets, thereby causing inaccuracies in positioning applications (e.g., Prikryl et al. 2014; Schrijver et al. 2015).

### ICMEs/sheaths as drivers of radiation belt electron flux variations

**Fig. 35:**
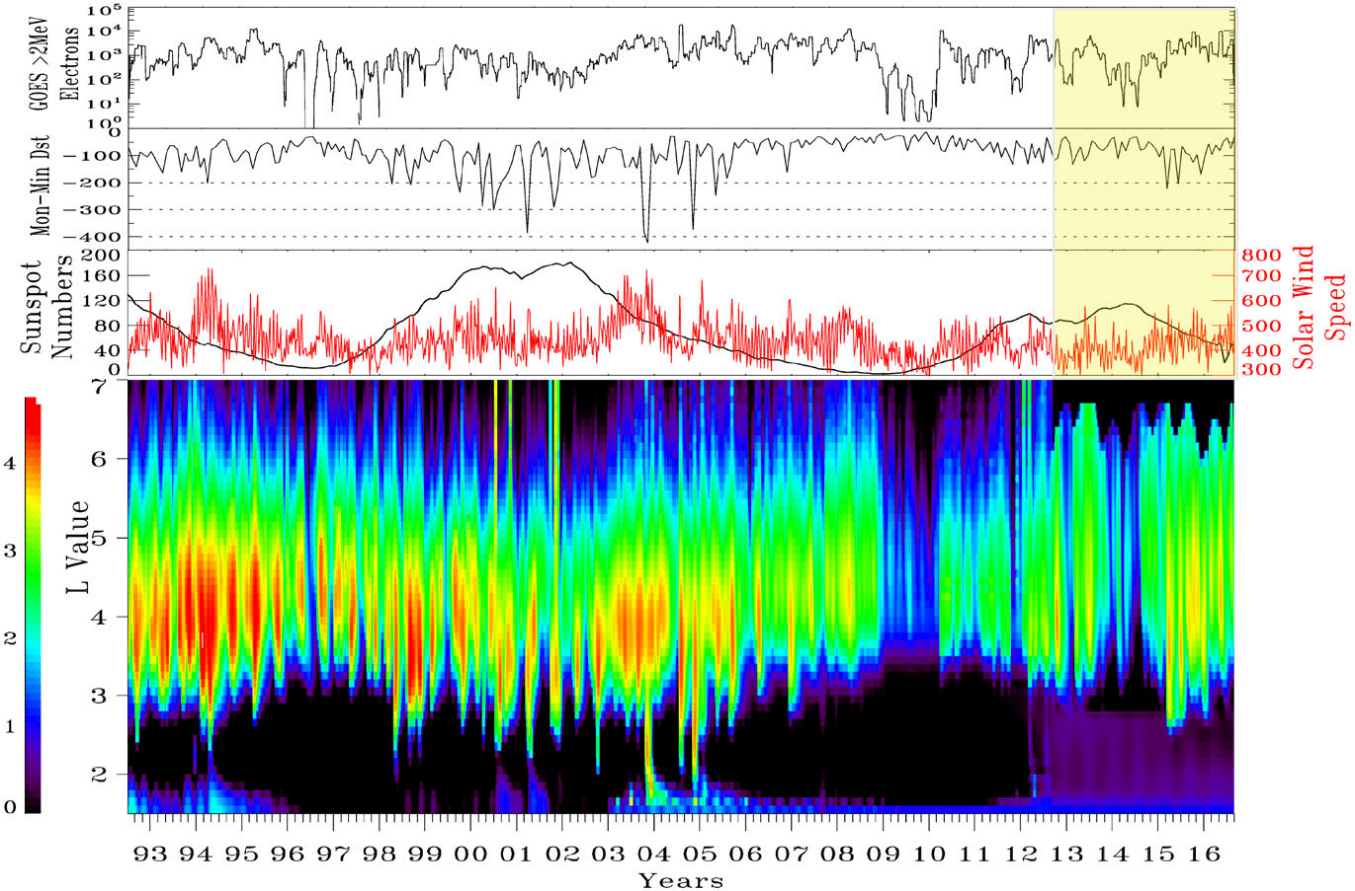
Combined long-term observations as measured by SAMPEX (in low Earth polar orbit) and Van Allen Probes (in the equatorial plane; the shaded period from 2012 on). Two top panels show the 27-day window-averaged $> 2$-MeV electron fluxes from geostationary spacecraft GOES, and the minimum value of the 1-h Dst index each month. The third panel shows the yearly window-averaged sunspot numbers (black curve) and the weekly window-averaged solar wind speed (km s$^{-1}$), red curve). The bottom panels show 27-day averaged $\sim 2$-MeV electron flux (units cm$^{-2}$ s$^{-1}$ sr$^{-1}$ MeV$^{-1}$) combined from SAMPEX and the REPT instrument on the Van Allen Probes. Image reproduced by permission from Li et al. (2017), copyright by the authors

Van Allen radiation belts are composed of energetic charged particles encircling the Earth extending from a distance of a few hundred kilometres above the Earth’s surface to beyond the geostationary orbit. The bottom panel of Fig. 35 from Li et al. (2017) shows the 27-day averaged $\sim 2$-MeV electron flux combined from the SAMPEX and Van Allen Probe data sorted according to the *L* parameter (*L* indicates the set of geomagnetic field lines, which cross the Earth’s magnetic equatorial plane at the Earth-radii equal to *L*). The figure shows that the belts are highly variable in particular during magnetospheric storms (see the second panel that shows the Dst index). The recent Van Allen Probes mission of NASA is revolutionizing our understanding of the belts; for instance, these twin spacecraft have highlighted the energy and distance dependence of the response of electron fluxes and shed new light on the role of various electron acceleration, transport and loss mechanisms (e.g., Reeves et al. 2013; Thorne et al. 2013). Some of the most dramatic features in Fig. 35, namely the penetration of high-energy electrons deep into the slot region and the inner belt ($L< 2.5$) occurred as the response of fast ICMEs with strong magnetic fields. Examples of this are the Halloween storms in late October 2003, the strong storm in November 2004, and the relatively deep penetration of electrons during the St. Patrick’s day storm in March 2015 (Li et al. 2017, and references therein).

Despite the recent progress many outstanding questions in the field remain and even predicting the overall response of the belts is still challenging. Figure 35 shows that while there is a good general agreement between the occurrence of geomagnetic storms (as measured by the Dst index) and relativistic electron flux enhancements, the relation is highly complex. A given geomagnetic storm can lead to enhancement of the electron fluxes, depletion of the electron population, or have no effect on the flux levels (Reeves et al. 2003). Any response during a storm is a complex combination of depletion associated with rapid reconfiguration and changes in the electron drift paths, acceleration processes associated with magnetotail reconnection events and with induced electric fields during substorms, and acceleration and loss processes associated with wave–particle interactions, including ULF, electromagnetic ion cyclotron (EMIC), VLF chorus and plasmaspheric hiss waves (e.g., Summers et al. 2014). Early works by Paulikas and Blake (1979) recognized the role of high-speed solar wind in creating electron flux enhancements of the outer Van Allen electron belt, but the response is much more subtle than being a simple function of speed (e.g., Reeves et al. 2011).

Shocks, sheaths and ICMEs/MCs have all distinct effects on radiation belts. First, the impact of the shock to the magnetosphere will launch a magnetosonic wave that propagates in the magnetosphere and the associated induced electric field can rapidly accelerate electrons to MeV energies (e.g., Foster et al. 2015; Kanekal et al. 2016). In particular, if the drift velocity of an electron matches the speed of the magnetosonic wave, the electron can “surf” on the wave and attain very high energies. The sheath regions in turn cause typically significant depletions of electron fluxes (e.g., Hietala et al. 2014; Kilpua et al. 2015a). This strong depleting tendency stems from the enhanced ULF wave power and dynamic pressure causing pitch angle scattering and radial diffusion, which combined with the highly compressed dayside magnetopause efficiently removes electrons from the belts. Although depleting processes clearly dominate during sheath passages, the sheaths are typically associated with strong substorm activity that can lead to effective injection of seed electrons into the radiation belt region (e.g., Li et al. 1998), as discussed in Kilpua et al. (2015a). These seed electrons may be energized by the later processes.

Similar to the overall geomagnetic storm characteristics the detailed response of the radiation belt electron fluxes to the MC depends strongly on its flux rope type, if present. If the flux rope has northward field, it can add to the depleting effect of the sheath, causing particularly long-lasting drop-outs of the high-energy electrons and magnetospheric quiescence (e.g., Alves et al. 2016). Sustained southward field in the MC will in turn erode the magnetosphere, enhancing the electron losses at the magnetopause, but as the MCs have generally lower dynamic pressure and lower ULF wave power, the losses are not as pronounced as during the sheaths (Kilpua et al. 2015a). On the other hand, the large-scale convection generates a seed particle population in the inner parts of the magnetotail, which can later be accelerated by a multitude of processes to relativistic energies. As shown by Kilpua et al. (2015a) the electron fluxes start typically rise during the end part of the fast ICMEs that have strongly southward magnetic fields. If a fast solar wind stream follows the ICME, the main enhancements, however, occur typically during this fast stream. All these results are consistent with studies emphasizing that the overall electron flux enhancements require sustained (up to several days) acceleration (e.g., Mann et al. 2016). The energetic electrons can stay at and inside the geostationary orbit for several weeks.

The shocks, sheaths and ICMEs have thus a major role both in removing relativistic electrons from the Van Allen belts (also including scattering to the atmosphere, see the next section) and in contributing to their generation, in particular by providing the seed population for further acceleration. As discussed earlier in this section fast and strong ICMEs (including also sheaths and shocks) were present in those cases where high-energy electrons penetrated deep into the belts.

In particular, the relativistic “killer” electrons within the Earth’s outer radiation belt are highly effective in producing a variety of space weather hazards to satellites and their systems in high-altitude space, as electronic components and many other materials degrade due to persistent irradiation. High-energy electrons can penetrate through the spacecraft shielding and get access to dielectric materials, which can over time lead to discharges that can be hazardous to the components or even entire subsystems (e.g., Horne et al. 2013; Baker and Lanzerotti 2016).

Since, as highlighted above, radiation belt electron flux dynamics differ for shocks, sheaths and MCs (including their flux rope types) it is important to understand better both their short and long-term effects, and the typical sequences at which they arrive. Other highly important aspects that also need to be studied more thoroughly include the short- and long-term electron flux responses from interacting ICMEs, where multiple (often merged) shocks, sheaths and ICMEs drive variations in the geomagnetic field.

### ICMEs/sheaths as drivers of atmospheric effects

The magnetospheric magnetic field is eroded and compressed during the ICME impact, but it still always protects the neutral atmosphere and ionosphere from the direct interaction with the solar wind. On the other hand, very high-energy particles originating from solar eruptions or ICME shocks easily cross the magnetic shield and can reach lower layers of the atmosphere, the mesosphere, at about 50–90 km altitude and down to the stratosphere at about 20–50 km (e.g., Seppälä et al. 2014). Most of these particles are SEPs accelerated at CME-driven shock waves either near the solar surface or further out in interplanetary space. Another indirect linkage is through the precipitation of particles that have been accelerated in the magnetosphere as response to ICMEs, as described in Sects. 6.1 and 6.3 (radiation belt electrons and ring current ions). In particular, we pointed out in Sect. 6.3 that ICME-driven sheath regions cause strong and prolonged depletion of radiation belt electron fluxes and a significant population may be lost to the atmosphere, e.g., by pitch-angle scattering due to interactions with the ULF waves.

The inclusion of the precipitating radiation belt high-energy electron population is one of the key current efforts in climate models (e.g., Andersson et al. 2014; Seppälä et al. 2014). This emphasizes the importance of ICMEs for understanding atmospheric chemistry and resulting climatological effects. Increasing electron precipitation and SEPs increases the production of HO$_\mathrm{x}$ and NO$_\mathrm{x}$ in the atmosphere (e.g., Verronen et al. 2011; Mironova et al. 2015). These are important reactive gases for the ozone balance in the middle atmosphere. Strong precipitation from the radiation belt and SEPs can hence lead to significant ozone loss and contribute also to the cooling of the stratosphere. There is also a strong potential for improved understanding of such local atmospheric processes when the roles of the shock, sheath and the flux rope are distinguished as different parts of the external driver.

Yet another significant, though little explored, space weather contribution from ICMEs is related to their potential to affect the overall conditions (density, composition, temperature, winds) of the Earth’s atmosphere above a few hundred km under different solar and geomagnetic conditions (e.g., Guo et al. 2016). Close study of the orbits of satellites in low Earth orbits and of satellites that have perigees at the few hundred km altitude range yield data that provide this atmospheric information. For example, CHAMP observations have shown significant changes in response of atmospheric densities to ICMEs (Forbes et al. 2005). Recently, Krauss et al. (2015) made a statistical analysis of the thermospheric neutral density content during ICME impacts. The authors found significant density enhancements, up to a factor of eight, as a response to ICMEs and a strong correlation with the magnitude of the enhancement and the magnitude of the southward magnetic field in the ICME. These data are needed and are used in models for the prediction of the obits of satellites and space debris around the Earth and any planetary body with an atmosphere. Statistical analysis by Chen et al. (2012) based on the CHAMP data showed that satellite orbit decay rates are typically larger during ICME-associated geomagnetic storms than those driven by SIRs, but longer duration of SIRs lead to overall greater decays.

### ICMEs and other planetary atmospheres

Planetary space weather is becoming increasingly important (e.g., Plainaki et al. 2016; Lilensten et al. 2014). Growing numbers of spacecraft explore our Solar System planets and concrete plans for future manned deep space missions are emerging. In addition, studies of planetary space weather can shed new light on many fundamental plasma physical processes and help to understand how astrophysical objects interact with their plasma surroundings. ICMEs have a key role in these studies. The nature of the interaction between ICMEs and the planets varies greatly with the evolution of the ICME properties with the distance from the Sun (see Sect. 5) and with the very different characteristics of magnetic fields and atmospheres of different planets.

Maybe one of the most important ICME-induced consequences concerning planetary space weather is their potential to enhance the ionospheric and atmospheric loss to space. Some of the planets do not have protecting global magnetic fields. For example, Venus and Mars have induced magnetospheres that form when the solar wind interacts directly with the planet’s ionosphere leading to significant losses from their ionosphere and atmosphere by various mechanisms (e.g., Dubinin et al. 2011). The process that is particularly important during the ICME encounter is the pick-up of ionospheric ions (mostly hydrogen and oxygen) by the solar wind motional electric field. As discussed earlier in this review, ICMEs can induce strong $-\mathbf{V}\times \mathbf{B}$ electric fields due their high speeds and enhanced magnetic fields. To maintain the pressure balance, the lost ions in the ionosphere are replaced from the neutral atmosphere, and the effects can be linked down to the surface of the planet. Another ICME-related mechanism is the atmospheric sputtering where a neutral atom obtains enough energy from a high-energy charged SEP to escape the gravitational binding. Both these processes can be enhanced when solar EUV and X-ray radiations are strong, as they heat the atmosphere. Some of the picked-up ions can also precipitate back to the atmosphere and enhance the sputtering.

**Fig. 36:**
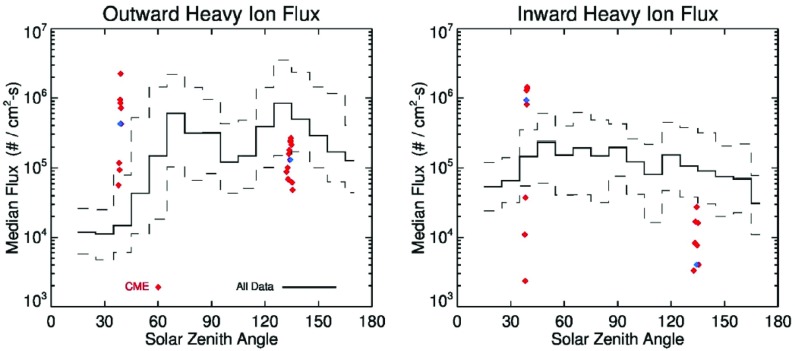
Atmospheric loss at Mars during encounters with ICMEs (red diamonds, blue show the median) compared to integrated loss through the mission duration (solid: median, dashed: first/fourth quartiles) as a function of solar zenith angle. Image reproduced by permission from Jakosky et al. (2015), copyright by AAAS

Space probes like Pioneer Venus Orbiter, Mars Express, Venus Express and the recent Mars Atmosphere and Volatile Evolution (MAVEN) spacecraft have revealed that ion losses from the ionosphere to space can be enhanced by an order of magnitude due to the ICME interaction (e.g., Luhmann et al. 2007; Futaana et al. 2008; Jakosky et al. 2015). Figure 36, from Jakosky et al. (2015) compares the outward and inward heavy ($> 9$ amu) ion fluxes from the Martian atmosphere as a function of solar zenith angle measured by MAVEN during an event on March 8–9, 2015 caused by two interacting ICMEs (red diamonds, blue diamonds give the median) to four-month median fluxes (solid curve, the dashed curves show the first and fourth quartiles). We note that based on the solar wind data shown Jakosky et al. (2015) these ICMEs were not merged into a complex ejecta, but their individual rotations can be distinguished. At the dayside (small zenith angles) outward fluxes are strongly enhanced during the ICMEs. As stated in Jakosky et al. (2015) this region is usually dominated by inward flows and the fluxes during this ICME were the strongest identified during the MAVEN lifespan so far. The results presented in Jakosky et al. (2015) imply that ICMEs and the associated SEPs can dramatically alter the overall morphology and dynamics of the magnetosphere, ionosphere and upper atmosphere of Mars.

We emphasize that separation of different ICME structures may also be of key importance for understanding the atmospheric effects. While the ICME flux rope proper can induce a strong and sustained motional electric field leading to significant pick-up losses, sheaths have high dynamic pressure that allows direct interactions to occur much closer to the surface of the planet. Solar wind pressure pulses have indeed shown to enhance the atmospheric loss both from Mars (e.g., Edberg et al. 2010) and from Venus (e.g., Edberg et al. 2011). However, the effects from fluctuating (sheath) versus smooth (flux rope) electric fields for the atmospheric escape have not been studied yet.

During its early days the Sun was considerably more active than today (e.g., Wood et al. 2005). Flares and CMEs of several orders of magnitudes larger than those released from the present Sun swept past the planets frequently. The fluxes of X-ray and UV emission, and SEPs were also much higher. These strong eruptions from the young Sun likely had a crucial role in the evolution of the atmospheres of the weakly magnetized planets, and could have even stripped away most of the atmosphere of Mars (e.g., Luhmann et al. 2007; Jakosky et al. 2015). The successive super-eruptions may have also affected significantly the atmosphere of the Earth. Simulations constrained by the Kepler Space Telescope observations suggest that the magnetospheric compression by ICMEs allowed energetic particles to penetrate deep into the atmosphere, affecting pre-biotic chemistry and atmospheric warming of the early Earth (Airapetian et al. 2016) .

### ICMEs beyond the Solar System

Stellar eruptions are by no means restricted to our Sun. The interest in effects of stellar CMEs and flares is quickly emerging as increasing numbers of Earth-like exoplanets are found in the habitable zones. These include a planet around the M-type dwarf star Gliese 876 (Rivera et al. 2005), Proxima b, circling around the Sun’s closest stellar neighbour Proxima Centauri (Anglada-Escudé et al. 2016), and the recent finding of seven terrestrial planets around TRAPPIST-1 (Gillon et al. 2017). The upcoming James Webb telescope can yield important information of their atmospheric constituents, and hence, find proxies for possible life. However, the atmospheres of these planets may be significantly influenced due to erosion as response to stellar eruptions (e.g., Airapetian et al. 2017).

CMEs and flares at other stars can be considerably more frequent, and several orders of magnitude more powerful, than at the Sun. Such super-CMEs and superflares can affect significantly the exoplanets, even their possibility to harbour life. Several Earth-like exoplanets are found circling around low-mass and cool M-type stars that are fully convective. They have remarkable magnetic activity indicated by large sunspots and strong optical flares as well as strong accompanying X-ray, ultraviolet, and radio emission. The habitable zones of such stars are typically at relatively close orbital distances. The planets in this region become tidally locked, and therefore, they rotate slowly and have relatively weak magnetic moments. As a consequence, they are particularly susceptible to effects of stellar eruptions, and even their whole atmosphere may be lost due to ICME interactions (e.g., Khodachenko et al. 2007; Lammer et al. 2007).

**Fig. 37:**
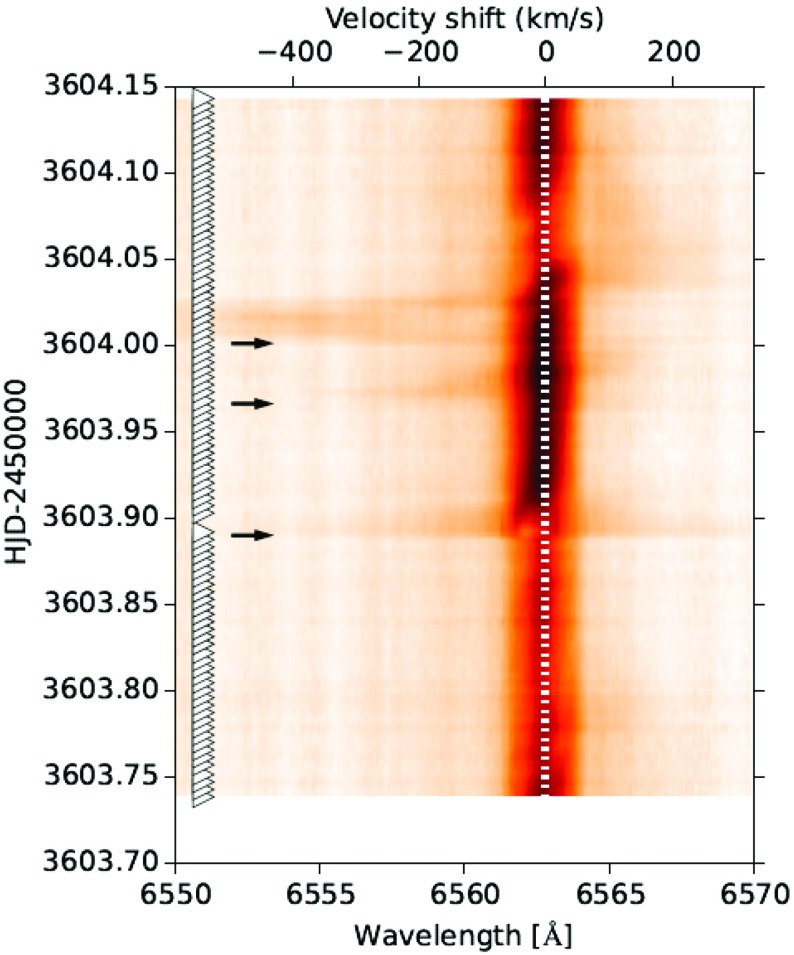
Blue-wing enhancements (shown by arrows) in dynamic $\hbox {H}_{\alpha }$ Balmer line spectra of a fully-convective M4 dwarf star V374 Pegasus occurred. The darkening colours indicate the increasing intensity of the $\hbox {H}_{\alpha }$ region. The vertical axis shows the time in Heliocentric Julian Days (HJD), starting from 2453603.70. Image reproduced by permission from Vida et al. (2016), copyright by ESO

While stellar flares can be detected, e.g., from optical light curves and X-ray observations there is no practical way yet to observe stellar CMEs. In addition, in-situ observations of stellar ICMEs will remain impossible until unforeseeable future. Some possible detections of stellar CMEs have been made through long-term spectrophotometric observations of Doppler shifted emission in $\hbox {H}_{\alpha }$ (e.g., Vida et al. 2016, and references therein). The dynamic $\hbox {H}_{\alpha }$ spectra in Fig. 37 from Vida et al. (2016) shows three distinct blue-wing enhancements that occurred in association with flares on a M4 dwarf V374 Pegasus. These blue-wings could hence signal the mass carried away from the star by a CME associated with a flare.

Using the $\hbox {H}_{\alpha }$ blue-wing flux enhancement the authors estimated that the lower limit for a CME mass was of the order of $10^{13}$ kg, and the estimated speed of the eruption was 675 km s$^{-1}$. These are comparable numbers to the typical masses and speeds of solar CMEs (see e.g., the LASCO CME catalogue^9^). However, much more massive and faster stellar CMEs have been speculated based on the solar flare/CME scaling laws (e.g., Aarnio et al. 2012; Drake et al. 2013), e.g., masses reaching up $10^{19}$ kg. The stellar CME observed by Houdebine et al. (1990), also using the optical spectra, had speed of about 5800 km s$^{-1}$. The estimated CME rate by Vida et al. (2016) using the V374 Pegasus X-ray luminosity was 15–60 eruptions per day, considerably higher than for the Sun (0.5–10 CMEs per day depending on the solar cycle phase). The recent more extensive surveys have, however, reported much less stellar CMEs than expected (Korhonen 2016). Some fraction of CMEs might skip the detection due to projection effects and/or too low densities, but it could also be possible that strong magnetic fields of active stars suppress the CMEs and prevent them from erupting (Drake et al. 2016).

Another possibility to search for stellar CMEs, and estimating their speeds, is through type II radio bursts generated at their shock waves. Such signals could be detected, e.g., with the Low Frequency Array (LOFAR) (e.g., Crosley et al. 2016). However, the Sun will remain for a long time our only source of direct information on CMEs and the studies on CME-induced effects on exoplanets have to rely largely on scaling laws derived using flare–CME and CME–ICME relationships from the Sun and our Solar System. It is also an interesting open question how strong sheath regions stellar ICMEs may have. In magnetically active stars, the likelihood of CME–CME interaction may also increase dramatically. It is well-documented in our Solar System (Liu et al. 2014) that the strongest magnetic fields and speeds in ICMEs are typically associated with interacting CMEs. Frequent interactions between super-CMEs may put exoplanets under very fierce forcing.

## Concluding remarks

Interplanetary coronal mass ejections are the most prominent transient structures in the heliosphere. Their paramount importance for space weather and coronal magnetic fields, as well as fundamental plasma physical interest keeps them at the a major focus of heliospheric research. We have here discussed their properties and signatures, a wide range of modelling efforts, evolution in the heliosphere and with solar cycle, and their consequences at Earth and other planetary space environments.

ICMEs have now been studied over several decades using a variety of spacecraft and instrumentation. While our knowledge of these gigantic structures has gradually grown, a number of major open questions remain. Even the identification of ICMEs in solar wind observations is often ambiguous, and their global configuration is not yet fully understood. Strong evidence suggests that the ICMEs can be described as flux rope loops, but alternative morphologies like a spheromak or writhed fields cannot be totally excluded. Moreover, the integrity of the ICME flux rope structure has also been questioned in recent studies. Multi-spacecraft observations with a range of separations are crucial for solving these issues. As ICMEs are vast structures (diameters being a significant fraction of the astronomical unit), single spacecraft observations give only very limited information on their properties. Unfortunately, only a few cases of multi-spacecraft encounters with comprehensive data are available today.

A major point in this review has been to highlight the sheath and ICME as separate structures. As we have discussed in this review these structures have distinctly different properties and geospace responses. Both better understanding of the physics and improved forecasting of space weather benefit from distinguishing the effects from these structures.

We also emphasised that the flux rope is sometimes embedded within a more complex ICME. A key question in the field is how these different structures (including layered sheath region) form and how remote-sensing and in-situ CME observations are connected. It is also clear that the diversity of ICMEs is a significant challenge to their comprehensive understanding and modelling.

The next decade looks vibrant for the ICME research. Two key missions, ESA’s Solar Orbiter and NASA’s Solar Probe Plus, will record ICMEs at varying distances in the heliosphere. Solar Probe Plus will plunge as close as ten solar radii from the Sun. An opportunity to witness ICMEs this close to the Sun will likely shed new light on many important questions, in particular related to their evolution and intrinsic morphology. Planetary missions like BepiColombo to Mercury will also provide chances for multi-spacecraft ICME observations and new information how ICMEs affect different planetary space environments.

## Footnotes

1 In this review the term CME refers to observations in the corona and to the phenomenon as a whole, whereas ICME is reserved to the interplanetary observations and properties of the propagating ejecta.

2
http://www.srl.caltech.edu/ACE/ASC/DATA/level3/icmetable2.htm.

3 We note that the term “MC” is often used in a more relaxed manner, and the events with significantly shorter durations than one day and weaker peak magnetic fields than 10 nT are often counted as MCs see e.g., Lepping et al. (2006) and the Richardson and Cane ICME list.

4
http://www-ssc.igpp.ucla.edu/~jlan/ACE/Level3/ICME_List_from_Lan_Jian.pdf.

5
http://www.srl.caltech.edu/ACE/ASC/DATA/level3/icmetable2.htm.

6
https://www.helcats-fp7.eu.

7 In the adiabatic process the temperature relates to density as $T \propto n^{\gamma _e-1}$.

8
ipshocks.fi.

9
https://cdaw.gsfc.nasa.gov/CME_list/.

## References

[CR1] Aarnio AN, Matt SP, Stassun KG (2012). Mass loss in pre-main-sequence stars via coronal mass ejections and implications for angular momentum loss. Astrophys J.

[CR2] Acuña MH, Ogilvie KW, Baker DN, Curtis SA, Fairfield DH, Mish WH (1995). The global geospace science program and its investigations. Space Sci Rev.

[CR3] Aguilar-Rodriguez E, Blanco-Cano X, Gopalswamy N (2006). Composition and magnetic structure of interplanetary coronal mass ejections at 1 AU. Adv Space Res.

[CR4] Airapetian VS, Glocer A, Gronoff G, Hébrard E, Danchi W (2016). Prebiotic chemistry and atmospheric warming of early Earth by an active young Sun. Nat Geosci.

[CR5] Airapetian VS, Glocer A, Khazanov GV, Loyd ROP, France K, Sojka J, Danchi WC, Liemohn MW (2017). How hospitable are space weather affected habitable zones? The role of ion escape. Astrophys J Lett.

[CR6] Al-Haddad N, Roussev II, Möstl C, Jacobs C, Lugaz N, Poedts S, Farrugia CJ (2011). On the internal structure of the magnetic field in magnetic clouds and interplanetary coronal mass ejections: writhe versus twist. Astrophys J Lett.

[CR7] Al-Haddad N, Nieves-Chinchilla T, Savani NP, Möstl C, Marubashi K, Hidalgo MA, Roussev II, Poedts S, Farrugia CJ (2013). Magnetic field configuration models and reconstruction methods for interplanetary coronal mass ejections. Sol Phys.

[CR8] Alves LR, Da Silva LA, Souza VM, Sibeck DG, Jauer PR, Vieira LEA, Walsh BM, Silveira MVD, Marchezi JP, Rockenbach M, Lago AD, Mendes O, Tsurutani BT, Koga D, Kanekal SG, Baker DN, Wygant JR, Kletzing CA (2016). Outer radiation belt dropout dynamics following the arrival of two interplanetary coronal mass ejections. Geophys Res Lett.

[CR9] Andersson ME, Verronen PT, Rodger CJ, Clilverd MA, Seppälä A (2014). Missing driver in the Sun–Earth connection from energetic electron precipitation impacts mesospheric ozone. Nature Commun.

[CR10] Anglada-Escudé G, Amado PJ, Barnes J, Berdiñas ZM, Butler RP, Coleman GAL, de La Cueva I, Dreizler S, Endl M, Giesers B, Jeffers SV, Jenkins JS, Jones HRA, Kiraga M, Kürster M, López-González MJ, Marvin CJ, Morales N, Morin J, Nelson RP, Ortiz JL, Ofir A, Paardekooper SJ, Reiners A, Rodríguez E, Rodríguez-López C, Sarmiento LF, Strachan JP, Tsapras Y, Tuomi M, Zechmeister M (2016). A terrestrial planet candidate in a temperate orbit around Proxima Centauri. Nature.

[CR11] Axford WI, Leer E, Skadron G (1977) The acceleration of cosmic rays by shock waves. In: 15th International Cosmic Ray Conference, vol 11, Bulgarian Academy of Sciences, pp 132–137

[CR12] Baker DN, Lanzerotti LJ (2016). Resource letter SW1: space weather. Am J Phys.

[CR13] Baker DN, Pulkkinen TI, Angelopoulos V, Baumjohann W, McPherron RL (1996). Neutral line model of substorms: past results and present view. J Geophys Res.

[CR14] Bale SD, Balikhin MA, Horbury TS, Krasnoselskikh VV, Kucharek H, Möbius E, Walker SN, Balogh A, Burgess D, Lembège B, Lucek EA, Scholer M, Schwartz SJ, Thomsen MF (2005). Quasi-perpendicular shock structure and processes. Space Sci Rev.

[CR15] Bame SJ, Asbridge JR, Feldman WC, Gosling JT, Zwickl RD (1981). Bi-directional streaming of solar wind electrons greater than 80 eV: ISEE evidence for a closed-field structure within the driver gas of an interplanetary shock. Geophys Res Lett.

[CR16] Bartels J (1932). Terrestrial-magnetic activity and its relations to solar phenomena. Terr Magn Atmos Electr.

[CR17] Bell AR (1978). The acceleration of cosmic rays in shock fronts—I. Mon Not R Astron Soc.

[CR18] Benacquista R, Rochel S, Rolland G (2017). Understanding the variability of magnetic storms caused by ICMEs. Ann Geophys.

[CR19] Berger MA, Field GB (1984). The topological properties of magnetic helicity. J Fluid Mech.

[CR20] Borovsky JE, Birn J (2014). The solar wind electric field does not control the dayside reconnection rate. J Geophys Res.

[CR21] Borovsky JE, Funsten HO (2003). Role of solar wind turbulence in the coupling of the solar wind to the Earth’s magnetosphere. J Geophys Res.

[CR22] Borrini G, Gosling JT, Bame SJ, Feldman WC (1982). An analysis of shock wave disturbances observed at 1 AU from 1971 through 1978. J Geophys Res.

[CR23] Bothmer V, Schwenn R (1994). Eruptive prominences as sources of magnetic clouds in the solar wind. Space Sci Rev.

[CR24] Bothmer V, Schwenn R (1996). Signatures of fast CMEs in interplanetary space. Adv Space Res.

[CR25] Bothmer V, Schwenn R (1998). The structure and origin of magnetic clouds in the solar wind. Ann Geophys.

[CR26] Burgess D, Lucek EA, Scholer M, Bale SD, Balikhin MA, Balogh A, Horbury TS, Krasnoselskikh VV, Kucharek H, Lembège B, Möbius E, Schwartz SJ, Thomsen MF, Walker SN (2005). Quasi-parallel shock structure and processes. Space Sci Rev.

[CR27] Burlaga LF (1988). Magnetic clouds and force-free fields with constant alpha. J Geophys Res.

[CR28] Burlaga LFE, Schwenn R, Marsch E (1991). Magnetic clouds. Physics of the inner heliosphere II: particles, waves and turbulence.

[CR29] Burlaga L, Sittler E, Mariani F, Schwenn R (1981). Magnetic loop behind an interplanetary shock: Voyager, Helios, and IMP 8 observations. J Geophys Res.

[CR30] Burlaga LF, Klein L, Sheeley NR, Michels DJ, Howard RA, Koomen MJ, Schwenn R, Rosenbauer H (1982). A magnetic cloud and a coronal mass ejection. Geophys Res Lett.

[CR31] Burlaga LF, Behannon KW, Klein LW (1987). Compound streams, magnetic clouds, and major geomagnetic storms. J Geophys Res.

[CR32] Burlaga LF, Lepping RP, Jones JA, Russell CT, Priest ER, Lee LC (1990). Global configuration of a magnetic cloud. Physics of magnetic flux ropes. Geophysical Monograph.

[CR33] Burlaga LF, Skoug RM, Smith CW, Webb DF, Zurbuchen TH, Reinard A (2001). Fast ejecta during the ascending phase of solar cycle 23: ACE observations, 1998–1999. J Geophys Res.

[CR34] Burlaga LF, Plunkett SP, St Cyr OC (2002). Successive CMEs and complex ejecta. J Geophys Res.

[CR35] Cane HV (1988). The large-scale structure of flare-associated interplanetary shocks. J Geophys Res.

[CR36] Cane HV (2000). Coronal mass ejections and forbush decreases. Space Sci Rev.

[CR37] Cane HV, Richardson IG (2003). Interplanetary coronal mass ejections in the near-Earth solar wind during 1996–2002. J Geophys Res.

[CR38] Cane HV, McGuire RE, von Rosenvinge TT (1986). Two classes of solar energetic particle events associated with impulsive and long-duration soft X-ray flares. Astrophys J.

[CR39] Cane HV, Reames DV, von Rosenvinge TT (1988). The role of interplanetary shocks in the longitude distribution of solar energetic particles. J Geophys Res.

[CR40] Cane HV, Richardson IG, Wibberenz G (1997). Helios 1 and 2 observations of particle decreases, ejecta, and magnetic clouds. J Geophys Res.

[CR41] Cane HV, von Rosenvinge TT, Cohen CMS, Mewaldt RA (2003). Two components in major solar particle events. Geophys Res Lett.

[CR42] Cane HV, Richardson IG, von Rosenvinge TT (2010). A study of solar energetic particle events of 1997–2006: their composition and associations. J Geophys Res.

[CR43] Carrington RC (1859). Description of a singular appearance seen in the Sun on September 1, 1859. Mon Not R Astron Soc.

[CR44] Chapman S, Ferraro VCA (1929). The electrical state of solar streams of corpuscles. Mon Not R Astron Soc.

[CR45] Chen PF (2011) Coronal mass ejections: models and their observational basis. Living Rev Sol Phys. 10.12942/lrsp-2011-1. http://www.livingreviews.org/lrsp-2011-1

[CR46] Chen GM, Xu J, Wang W, Lei J, Burns AG (2012). A comparison of the effects of CIR- and CME-induced geomagnetic activity on thermospheric densities and spacecraft orbits: case studies. J Geophys Res.

[CR47] Cliver EW, Kahler SW, Neidig DF, Cane HV, Richardson IG, Kallenrode MB, Wibberenz G (1995) Extreme “Propagation” of solar energetic particles. In: Iucci N, Lamanna E (eds) 24th International Cosmic Ray Conference, vol 4, IUPAP, p. 257

[CR48] Cocconi G, Gold T, Greisen K, Hayakawa S, Morrison P (1958). The cosmic ray flare effect. Nuovo Cimento.

[CR49] Cremades H, Bothmer V, Tripathi D (2006). Properties of structured coronal mass ejections in solar cycle 23. Adv Space Res.

[CR50] Crooker NU (2005) ICME-CME connections: outstanding questions (Tutorial Talk). In: Fleck B, Zurbuchen TH, Lacoste H (eds) Solar wind 11/SOHO 16, Connecting Sun and Heliosphere, ESA Special Publication, vol 592

[CR51] Crooker NU, Horbury TS (2006). Solar imprint on ICMEs, their magnetic connectivity, and heliospheric evolution. Space Sci Rev.

[CR52] Crooker NU, Intriligator DS (1996). A magnetic cloud as a distended flux rope occlusion in the heliospheric current sheet. J Geophys Res.

[CR53] Crooker NU, Gosling JT, Kahler SW (1998). Magnetic clouds at sector boundaries. J Geophys Res.

[CR54] Crooker NU, Forsyth R, Rees A, Gosling JT, Kahler SW (2004). Counterstreaming electrons in magnetic clouds near 5 AU. J Geophys Res.

[CR55] Crosley MK, Osten RA, Broderick JW, Corbel S, Eislöffel J, Grießmeier JM, van Leeuwen J, Rowlinson A, Zarka P, Norman C (2016). The search for signatures of transient mass loss in active stars. Astrophys J.

[CR56] Das I, Opher M, Evans R, Loesch C, Gombosi TI (2011). Evolution of piled-up compressions in modeled coronal mass ejection sheaths and the resulting sheath structures. Astrophys J.

[CR57] Dasso S, Mandrini CH, Démoulin P, Luoni ML, Gulisano AM (2005). Large scale MHD properties of interplanetary magnetic clouds. Adv Space Res.

[CR58] Dasso S, Nakwacki MS, Démoulin P, Mandrini CH (2007). Progressive transformation of a flux rope to an ICME. Comparative analysis using the direct and fitted expansion methods. Sol Phys.

[CR59] Davies JA, Harrison RA, Rouillard AP, Sheeley NR, Perry CH, Bewsher D, Davis CJ, Eyles CJ, Crothers SR, Brown DS (2009). A synoptic view of solar transient evolution in the inner heliosphere using the heliospheric imagers on STEREO. Geophys Res Lett.

[CR60] de Lucas A, Schwenn R, dal Lago A, Marsch E, Clúa de Gonzalez AL (2011). Interplanetary shock wave extent in the inner heliosphere as observed by multiple spacecraft. J Atmos Terr Phys.

[CR61] Démoulin P, Dasso S (2009). Causes and consequences of magnetic cloud expansion. Astron Astrophys.

[CR62] Démoulin P, Janvier M, Dasso S (2016). Magnetic flux and helicity of magnetic clouds. Sol Phys.

[CR63] Dere KP, Brueckner GE, Howard RA, Michels DJ, Delaboudiniere JP (1999). LASCO and EIT observations of helical structure in coronal mass ejections. Astrophys J.

[CR64] Dierckxsens M, Tziotziou K, Dalla S, Patsou I, Marsh MS, Crosby NB, Malandraki O, Tsiropoula G (2015). Relationship between solar energetic particles and properties of flares and cmes: statistical analysis of solar cycle 23 events. Sol Phys.

[CR65] Drake JJ, Cohen O, Garraffo C, Kashyap V (2016) Stellar flares and the dark energy of CMEs. In: Kosovichev AG, Hawley SL, Heinzel P (eds) Solar and stellar flares and their effects on planets, IAU symposium, vol 320, pp 196–201. 10.1017/S1743921316000260. arXiv:1610.05185

[CR66] Drake JJ, Cohen O, Yashiro S, Gopalswamy N (2013). Implications of mass and energy loss due to coronal mass ejections on magnetically active stars. Astrophys J.

[CR67] Dresing N, Gómez-Herrero R, Klassen A, Heber B, Kartavykh Y, Dröge W (2012). The large longitudinal spread of solar energetic particles during the 17 January 2010 solar event. Sol Phys.

[CR68] Drury LO (1983). An introduction to the theory of diffusive shock acceleration of energetic particles in tenuous plasmas. Rep Prog Phys.

[CR69] Dubinin E, Fraenz M, Fedorov A, Lundin R, Edberg N, Duru F, Vaisberg O (2011). Ion energization and escape on mars and venus. Space Sci Rev.

[CR70] Dungey JW (1961). Interplanetary magnetic field and the auroral zones. Phys Rev Lett.

[CR71] Ebert RW, Dayeh MA, Desai MI, Jian LK, Li G, Mason GM (2016). Multi-spacecraft analysis of energetic heavy ion and interplanetary shock properties in energetic storm particle events near 1 au. Astrophys J.

[CR72] Echer E, Gonzalez WD, Tsurutani BT, Gonzalez ALC (2008). Interplanetary conditions causing intense geomagnetic storms ($$Dst\le -100$$ nT) during solar cycle 23 (1996–2006). J Geophys Res.

[CR73] Edberg NJT, Nilsson H, Williams AO, Lester M, Milan SE, Cowley SWH, Fränz M, Barabash S, Futaana Y (2010). Pumping out the atmosphere of Mars through solar wind pressure pulses. Geophys Res Lett.

[CR74] Edberg NJT, Nilsson H, Futaana Y, Stenberg G, Lester M, Cowley SWH, Luhmann JG, McEnulty TR, Opgenoorth HJ, Fedorov A, Barabash S, Zhang TL (2011). Atmospheric erosion of Venus during stormy space weather. J Geophys Res.

[CR75] Eyles CJ, Simnett GM, Cooke MP, Jackson BV, Buffington A, Hick PP, Waltham NR, King JM, Anderson PA, Holladay PE (2003). The solar mass ejection imager (SMEI). Solar Phys.

[CR76] Farrugia CJ, Vasquez B, Richardson IG, Torbert RB, Burlaga LF, Biernat HK, Mühlbachler S, Ogilvie KW, Lepping RP, Scudder JD, Berdichevsky DE, Semenov VS, Kubyshkin IV, Phan TD, Lin RP (2001). A reconnection layer associated with a magnetic cloud. Adv Space Res.

[CR77] Farrugia CJ, Erkaev NV, Taubenschuss U, Shaidurov VA, Smith CW, Biernat HK (2008). A slow mode transition region adjoining the front boundary of a magnetic cloud as a relic of a convected solar wind feature: observations and MHD simulation. J Geophys Res.

[CR78] Feng H, Wang J (2013). Magnetic-reconnection exhausts in the sheath of magnetic clouds. Astron Astrophys.

[CR79] Fenrich FR, Luhmann JG (1998). Geomagnetic response to magnetic clouds of different polarity. Geophys Res Lett.

[CR80] Forbes JM, Lu G, Bruinsma S, Nerem S, Zhang X (2005). Thermosphere density variations due to the 15–24 April 2002 solar events from CHAMP/STAR accelerometer measurements. J Geophys Res.

[CR81] Forbush SE (1937). On the effects in cosmic-ray intensity observed during the recent magnetic storm. Phys Rev.

[CR82] Forsyth RJ, Bothmer V, Cid C, Crooker NU, Horbury TS, Kecskemety K, Klecker B, Linker JA, Odstrčil D, Reiner MJ, Richardson IG, Rodriguez-Pacheco J, Schmidt JM, Wimmer-Schweingruber RF (2006). ICMEs in the inner heliosphere: origin, evolution and propagation effects. Report of Working Group G. Space Sci Rev.

[CR83] Foster JC, Wygant JR, Hudson MK, Boyd AJ, Baker DN, Erickson PJ, Spence HE (2015). Shock-induced prompt relativistic electron acceleration in the inner magnetosphere. J Geophys Res.

[CR84] Fox NJ, Peredo M, Thompson BJ (1998). Cradle to grave tracking of the January 6–11, 1997 Sun–Earth connection event. Geophys Res Lett.

[CR85] Futaana Y, Barabash S, Yamauchi M, McKenna-Lawlor S, Lundin R, Luhmann JG, Brain D, Carlsson E, Sauvaud JA, Winningham JD, Frahm RA, Wurz P, Holmström M, Gunell H, Kallio E, Baumjohann W, Lammer H, Sharber JR, Hsieh KC, Andersson H, Grigoriev A, Brinkfeldt K, Nilsson H, Asamura K, Zhang TL, Coates AJ, Linder DR, Kataria DO, Curtis CC, Sandel BR, Fedorov A, Mazelle C, Thocaven JJ, Grande M, Koskinen HEJ, Sales T, Schmidt W, Riihela P, Kozyra J, Krupp N, Woch J, Fränz M, Dubinin E, Orsini S, Cerulli-Irelli R, Mura A, Milillo A, Maggi M, Roelof E, Brandt P, Szego K, Scherrer J, Bochsler P (2008). Mars express and Venus express multi-point observations of geoeffective solar flare events in December 2006. Planet Space Sci.

[CR86] Ganushkina NY, Amariutei OA, Welling D, Heynderickx D (2015). Nowcast model for low-energy electrons in the inner magnetosphere. Space Weather.

[CR87] Garrett HB (1981). The charging of spacecraft surfaces. Rev Geophys Space Phys.

[CR88] Gazis PR, Balogh A, Dalla S, Decker R, Heber B, Horbury T, Kilchenmann A, Kota J, Kucharek H, Kunow H, Lario D, Potgieter MS, Richardson JD, Riley P, Rodriguez L, Siscoe G, von Steiger R (2006). ICMEs at high latitudes and in the outer heliosphere. Report of Working Group H. Space Sci Rev.

[CR89] Gibson SE, Low BC (2000). Three-dimensional and twisted: an MHD interpretation of on-disk observational characteristics of coronal mass ejections. J Geophys Res.

[CR90] Gillon M, Triaud AHMJ, Demory BO, Jehin E, Agol E, Deck KM, Lederer SM, de Wit J, Burdanov A, Ingalls JG, Bolmont E, Leconte J, Raymond SN, Selsis F, Turbet M, Barkaoui K, Burgasser A, Burleigh MR, Carey SJ, Chaushev A, Copperwheat CM, Delrez L, Fernandes CS, Holdsworth DL, Kotze EJ, Van Grootel V, Almleaky Y, Benkhaldoun Z, Magain P, Queloz D (2017). Seven temperate terrestrial planets around the nearby ultracool dwarf star TRAPPIST-1. Nature.

[CR91] Gold T, Hoyle F (1960). On the origin of solar flares. Mon Not R Astron Soc.

[CR92] Goldstein H (1983) On the field configuration in magnetic clouds. In: Neugebauer M (ed) JPL solar wind five, Washington, DC, NASA conference publication, vol 228, pp 731–733. http://hdl.handle.net/2060/19840005057

[CR93] Gómez-Herrero R, Dresing N, Klassen A, Heber B, Lario D, Agueda N, Malandraki OE, Blanco JJ, Rodríguez-Pacheco J, Banjac S (2015). Circumsolar energetic particle distribution on 2011 November 3. Astrophys J.

[CR94] Gonzalez WD, Joselyn JA, Kamide Y, Kroehl HW, Rostoker G, Tsurutani BT, Vasyliunas VM (1994). What is a geomagnetic storm?. J Geophys Res.

[CR95] Gonzalez WD, de Gonzalez ALC, Dal Lago A, Tsurutani BT, Arballo JK, Lakhina GK, Buti B, Ho CM, Wu ST (1998). Magnetic cloud field intensities and solar wind velocities. Geophys Res Lett.

[CR96] Good SW, Forsyth RJ (2016). Interplanetary coronal mass ejections observed by MESSENGER and Venus express. Sol Phys.

[CR97] Gopalswamy N (2016). History and development of coronal mass ejections as a key player in solar terrestrial relationship. Geosci Lett.

[CR98] Gopalswamy N, Lara A, Lepping RP, Kaiser ML, Berdichevsky D, St Cyr OC (2000). Interplanetary acceleration of coronal mass ejections. Geophys Res Lett.

[CR99] Gopalswamy N, Mäkelä P, Xie H, Akiyama S, Yashiro S (2010) Solar sources of “Driverless” interplanetary shocks. In: Maksimovic M (ed) Twelfth international solar wind conference, American Institute of Physics, Melville, NY, AIP conference proceedings, vol 1216, pp 452–458. 10.1063/1.3395902

[CR100] Gosling JT (1990) Coronal mass ejections and magnetic flux ropes in interplanetary space. In: Physics of magnetic flux ropes Geophysical Monograph, vol 58, American Geophysical Union, Washington, DC, pp 343–364. 10.1029/GM058p0343

[CR101] Gosling JT (1997) Coronal mass ejections: an overview. In: Crooker N, Joselyn JA, Feynman J (eds) Coronal mass ejections. Geophysical Monograph, vol 99, American Geophysical Union, Washington, DC, pp 9–16. 10.1029/GM099p0009

[CR102] Gosling JT (1993). The solar flare myth. J Geophys Res.

[CR103] Gosling JT (1999). On the determination of electron polytrope indices within coronal mass ejections in the solar wind. J Geophys Res.

[CR104] Gosling JT, McComas DJ (1987). Field line draping about fast coronal mass ejecta: a source of strong out-of-the-ecliptic interplanetary magnetic fields. Geophys Res Lett.

[CR105] Gosling JT, Pizzo V, Bame SJ (1973). Anomalously low proton temperatures in the solar wind following interplanetary shock waves—evidence for magnetic bottles?. J Geophys Res.

[CR106] Gosling JT, Baker DN, Bame SJ, Feldman WC, Zwickl RD, Smith EJ (1987). Bidirectional solar wind electron heat flux events. J Geophys Res.

[CR107] Gosling JT, McComas DJ, Phillips JL, Bame SJ (1991). Geomagnetic activity associated with earth passage of interplanetary shock disturbances and coronal mass ejections. J Geophys Res.

[CR108] Gosling JT, McComas DJ, Phillips JL, Weiss LA, Pizzo VJ, Goldstein BE, Forsyth RJ (1994). A new class of forward–reverse shock pairs in the solar wind. Geophys Res Lett.

[CR109] Gosling JT, Bame SJ, McComas DJ, Phillips JL, Balogh A, Strong KT (1995). Coronal mass ejections at high heliographic latitudes: ULYSSES. Space Sci Rev.

[CR110] Grechnev VV, Kiselev VI, Meshalkina NS, Chertok IM (2015). Relations between microwave bursts and near-earth high-energy proton enhancements and their origin. Sol Phys.

[CR111] Gruesbeck JR, Lepri ST, Zurbuchen TH (2012). Two-plasma model for low charge state interplanetary coronal mass ejection observations. Astrophys J.

[CR112] Gulisano AM, Démoulin P, Dasso S, Ruiz ME, Marsch E (2010). Global and local expansion of magnetic clouds in the inner heliosphere. Astron Astrophys.

[CR113] Guo J, Wei F, Feng X, Forbes JM, Wang Y, Liu H, Wan W, Yang Z, Liu C (2016). Prolonged multiple excitation of large-scale Traveling Atmospheric Disturbances (TADs) by successive and interacting coronal mass ejections. J Geophys Res.

[CR114] Harrison RA, Davis CJ, Eyles CJ (2005). The STEREO heliospheric imager: how to detect CMEs in the heliosphere. Adv Space Res.

[CR115] Henke T, Woch J, Schwenn R, Mall U, Gloeckler G, von Steiger R, Forsyth RJ, Balogh A (2001). Ionization state and magnetic topology of coronal mass ejections. J Geophys Res.

[CR116] Hidalgo MA (2016). A global magnetic topology model for magnetic clouds—IV. Astrophys J.

[CR117] Hidalgo MA, Cid C, Vinas AF, Sequeiros J (2002). A non-force-free approach to the topology of magnetic clouds in the solar wind. J Geophys Res.

[CR118] Hietala H, Kilpua EKJ, Turner DL, Angelopoulos V (2014). Depleting effects of ICME-driven sheath regions on the outer electron radiation belt. Geophys Res Lett.

[CR119] Hildner E (1977) Mass ejections from the solar corona into interplanetary space. In: Shea MA, Smart DF, Wu ST (eds) Study of travelling interplanetary phenomena, vol 71. Astrophysics and Space Science Library, D. Reidel, Dordrecht, pp 3–20. 10.1007/978-90-277-0860-1_1

[CR120] Hirshberg J, Alksne A, Colburn DS, Bame SJ, Hundhausen AJ (1970). Observation of a solar flare induced interplanetary shock and helium-enriched driver gas. J Geophys Res.

[CR121] Hirshberg J, Bame SJ, Robbins DE (1972). Solar flares and solar wind helium enrichments: July 1965 July 1967. Sol Phys.

[CR122] Hood AW, Priest ER (1981). Critical conditions for magnetic instabilities in force-free coronal loops. Geophys Astrophys Fluid Dyn.

[CR123] Horne RB, Glauert SA, Meredith NP, Boscher D, Maget V, Heynderickx D, Pitchford D (2013). Space weather impacts on satellites and forecasting the Earth’s electron radiation belts with SPACECAST. Space Weather.

[CR124] Houdebine ER, Foing BH, Rodonò M (1990). Dynamics of flares on late-type dMe stars. I. Flare mass ejections and stellar evolution. Astron Astrophys.

[CR125] Hu Q, Sonnerup BUÖ (2002). Reconstruction of magnetic clouds in the solar wind: orientations and configurations. J Geophys Res.

[CR126] Hu Q, Qiu J, Krucker S (2015). Magnetic field line lengths inside interplanetary magnetic flux ropes. J Geophys Res.

[CR127] Hundhausen AJ, Sawyer CB, House L, Illing RME, Wagner WJ (1984). Coronal mass ejections observed during the solar maximum mission: latitude distribution and rate of occurrence. J Geophys Res.

[CR128] Huttunen K, Koskinen H (2004). Importance of post-shock streams and sheath region as drivers of intense magnetospheric storms and high-latitude activity. Ann Geophys.

[CR129] Huttunen KEJ, Koskinen HEJ, Schwenn R (2002). Variability of magnetospheric storms driven by different solar wind perturbations. J Geophys Res.

[CR130] Huttunen KEJ, Schwenn R, Bothmer V, Koskinen HEJ (2005). Properties and geoeffectiveness of magnetic clouds in the rising, maximum and early declining phases of solar cycle 23. Ann Geophys.

[CR131] Illing RME, Hundhausen AJ (1983). Possible observation of a disconnected magnetic structure in a coronal transient. J Geophys Res.

[CR132] Intriligator DS, Rees A, Horbury TS (2008). First analyses of planar magnetic structures associated with the Halloween 2003 events from the Earth to Voyager 1 at 93 AU. J Geophys Res.

[CR133] Isavnin A (2016). FRiED: a novel three-dimensional model of coronal mass ejections. Astrophys J.

[CR134] Isavnin A, Kilpua EKJ, Koskinen HEJ (2011). Grad–Shafranov reconstruction of magnetic clouds: overview and improvements. Sol Phys.

[CR135] Isavnin A, Vourlidas A, Kilpua EKJ (2014). Three-dimensional evolution of flux-rope CMEs and its relation to the local orientation of the heliospheric current sheet. Sol Phys.

[CR136] Ivanov KG, Harshiladze AF, Eroshenko EG, Stiazhkin VA (1989). Configuration, structure, and dynamics of magnetic clouds from solar flares in light of measurements on broad Vega 1 and Vega 2 in January–February 1986. Sol Phys.

[CR137] Jacobs C, Roussev II, Lugaz N, Poedts S (2009). The internal structure of coronal mass ejections: are all regular magnetic clouds flux ropes?. Astrophys J Lett.

[CR138] Jakosky BM, Grebowsky JM, Luhmann JG, Connerney J, Eparvier F, Ergun R, Halekas J, Larson D, Mahaffy P, McFadden J, Mitchell DF, Schneider N, Zurek R, Bougher S, Brain D, Ma YJ, Mazelle C, Andersson L, Andrews D, Baird D, Baker D, Bell JM, Benna M, Chaffin M, Chamberlin P, Chaufray YY, Clarke J, Collinson G, Combi M, Crary F, Cravens T, Crismani M, Curry S, Curtis D, Deighan J, Delory G, Dewey R, DiBraccio G, Dong C, Dong Y, Dunn P, Elrod M, England S, Eriksson A, Espley J, Evans S, Fang X, Fillingim M, Fortier K, Fowler CM, Fox J, Gröller H, Guzewich S, Hara T, Harada Y, Holsclaw G, Jain SK, Jolitz R, Leblanc F, Lee CO, Lee Y, Lefevre F, Lillis R, Livi R, Lo D, Mayyasi M, McClintock W, McEnulty T, Modolo R, Montmessin F, Morooka M, Nagy A, Olsen K, Peterson W, Rahmati A, Ruhunusiri S, Russell CT, Sakai S, Sauvaud JA, Seki K, Steckiewicz M, Stevens M, Stewart AIF, Stiepen A, Stone S, Tenishev V, Thiemann E, Tolson R, Toublanc D, Vogt M, Weber T, Withers P, Woods T, Yelle R (2015). MAVEN observations of the response of Mars to an interplanetary coronal mass ejection. Science.

[CR139] Janvier M, Démoulin P, Dasso S (2014). Mean shape of interplanetary shocks deduced from in situ observations and its relation with interplanetary CMEs. Astron Astrophys.

[CR140] Janvier M, Dasso S, Démoulin P, Masías-Meza JJ, Lugaz N (2015). Comparing generic models for interplanetary shocks and magnetic clouds axis configurations at 1 AU. J Geophys Res.

[CR141] Jian L, Russell CT, Luhmann JG, Skoug RM (2006). Properties of interplanetary coronal mass ejections at one AU during 1995–2004. Sol Phys.

[CR142] Jones GH, Balogh A (2000). Context and heliographic dependence of heliospheric planar magnetic structures. J Geophys Res.

[CR143] Jordan AP, Spence HE, Blake JB, Shaul DNA (2011). Revisiting two-step Forbush decreases. J Geophys Res.

[CR144] Kahler SW (1982). The role of the big flare syndrome in correlations of solar energetic proton fluxes and associated microwave burst parameters. J Geophys Res.

[CR145] Kahler SW, Reames DV (1991). Probing the magnetic topologies of magnetic clouds by means of solar energetic particles. J Geophys Res.

[CR146] Kahler SW, Sheeley NR, Howard RA, Koomen MJ, Michels DJ, McGuire RE, von Rosenvinge TT, Reames DV (1984). Associations between coronal mass ejections and solar energetic proton events. J Geophys Res.

[CR147] Kajdič P, Blanco-Cano X, Aguilar-Rodriguez E, Russell CT, Jian LK, Luhmann JG (2012). Waves upstream and downstream of interplanetary shocks driven by coronal mass ejections. J Geophys Res.

[CR148] Kamide Y, McPherron RL, Gonzalez WD, Hamilton DC, Hudson HS, Joselyn JA, Kahler SW, Lyons LR, Lundstedt H, Szuszczewicz E (1997) Magnetic storms: current understanding and outstanding questions. In: Tsurutani BT, Gonzalez WD, Kamide Y, Arballo JK (eds) Magnetic storms. Geophysical Monograph, vol 98, American Geophysical Union, Washington, DC, pp 1–19. 10.1029/GM098p0001

[CR149] Kanekal SG, Baker DN, Fennell JF, Jones A, Schiller Q, Richardson IG, Li X, Turner DL, Califf S, Claudepierre SG, Wilson LB, Jaynes A, Blake JB, Reeves GD, Spence HE, Kletzing CA, Wygant JR (2016). Prompt acceleration of magnetospheric electrons to ultrarelativistic energies by the 17 March 2015 interplanetary shock. J Geophys Res.

[CR150] Kataoka R, Watari S, Shimada N, Shimazu H, Marubashi K (2005). Downstream structures of interplanetary fast shocks associated with coronal mass ejections. Geophys Res Lett.

[CR151] Kataoka R, Shiota D, Kilpua E, Keika K (2015). Pileup accident hypothesis of magnetic storm on 17 March 2015. Geophys Res Lett.

[CR152] Kaymaz Z, Siscoe G (2006). Field-line draping around ICMEs. Sol Phys.

[CR153] Khodachenko ML, Ribas I, Lammer H, Grießmeier JM, Leitner M, Selsis F, Eiroa C, Hanslmeier A, Biernat HK, Farrugia CJ, Rucker HO (2007). Coronal mass ejection (CME) activity of low mass M stars as an important factor for the habitability of terrestrial exoplanets. I. CME impact on expected magnetospheres of Earth-like exoplanets in close-in habitable zones. Astrobiology.

[CR154] Kilpua EKJ, Pomoell J, Vourlidas A, Vainio R, Luhmann J, Li Y, Schroeder P, Galvin AB, Simunac K (2009). STEREO observations of interplanetary coronal mass ejections and prominence deflection during solar minimum period. Ann Geophys.

[CR155] Kilpua EKJ, Jian LK, Li Y, Luhmann JG, Russell CT (2011). Multipoint ICME encounters: pre-STEREO and STEREO observations. J Atmos Terr Phys.

[CR156] Kilpua EKJ, Li Y, Luhmann JG, Jian LK, Russell CT (2012). On the relationship between magnetic cloud field polarity and geoeffectiveness. Ann Geophys.

[CR157] Kilpua EKJ, Hietala H, Koskinen HEJ, Fontaine D, Turc L (2013a) Magnetic field and dynamic pressure ULF fluctuations in coronal-mass-ejection-driven sheath regions. Ann Geophys 31:1559–1567. 10.5194/angeo-31-1559-2013

[CR158] Kilpua EKJ, Isavnin A, Vourlidas A, Koskinen HEJ, Rodriguez L (2013b) On the relationship between interplanetary coronal mass ejections and magnetic clouds. Ann Geophys 31:1251–1265. 10.5194/angeo-31-1251-2013

[CR159] Kilpua EKJ, Hietala H, Turner DL, Koskinen HEJ, Pulkkinen TI, Rodriguez JV, Reeves GD, Claudepierre SG, Spence HE (2015). Unraveling the drivers of the storm time radiation belt response. Geophys Res Lett.

[CR160] Kilpua EKJ, Lumme E, Andreeova K, Isavnin A, Koskinen HEJ (2015). Properties and drivers of fast interplanetary shocks near the orbit of the Earth (1995–2013). J Geophys Res.

[CR161] Kilpua E, Balogh A, von Steiger R, Liu Y (2017) Geoeffective properties of solar transients and stream interaction regions. Space Sci Rev. 10.1007/s11214-017-0411-3

[CR162] Klein LW, Burlaga LF (1982). Interplanetary magnetic clouds at 1 AU. J Geophys Res.

[CR163] Korhonen H (2016). Properties of stellar activity cycles. IAU Focus Meeting.

[CR164] Koskinen HEJ, Huttunen KEJ (2006). Geoeffectivity of coronal mass ejections. Space Sci Rev.

[CR165] Krauss S, Temmer M, Veronig A, Baur O, Lammer H (2015). Thermospheric and geomagnetic responses to interplanetary coronal mass ejections observed by ACE and GRACE: statistical results. J Geophys Res.

[CR166] Krymskii GF (1977). A regular mechanism for the acceleration of charged particles on the front of a shock wave. Akademiia Nauk SSSR Doklady.

[CR167] Laming JM (2015) The FIP and inverse FIP effects in solar and stellar coronae. Living Rev Sol Phys 12:lrsp-2015-2. 10.1007/lrsp-2015-2. http://www.livingreviews.org/lrsp-2015-2. arXiv:1504.08325

[CR168] Lammer H, Lichtenegger HIM, Kulikov YN, Grießmeier JM, Terada N, Erkaev NV, Biernat HK, Khodachenko ML, Ribas I, Penz T, Selsis F (2007). Coronal mass ejection (CME) activity of low mass M stars as an important factor for the habitability of terrestrial exoplanets. II. CME-induced ion pick up of Earth-like exoplanets in close-In habitable zones. Astrobiology.

[CR169] Lanzerotti LJ, LaFleur K, Maclennan CG, Maurer DW (1998). Geosynchronous spacecraft charging in January 1997. Geophys Res Lett.

[CR170] Lario D, Decker RB, Roelof EC, Viñas AF (2015). Energetic particle pressure at interplanetary shocks: STEREO-A observations. Astrophys J.

[CR171] Lario D, Kwon RY, Richardson IG, Raouafi NE, Thompson BJ, von Rosenvinge TT, Mays ML, Mäkelä PA, Xie H, Bain HM, Zhang M, Zhao L, Cane HV, Papaioannou A, Thakur N, Riley P (2017). The solar energetic particle event of 2010 August 14: connectivity with the solar source inferred from multiple spacecraft observations and modeling. Astrophys J.

[CR172] Larson DE, Lin RP, McTiernan JM, McFadden JP, Ergun RE, McCarthy M, Rème H, Sanderson TR, Kaiser M, Lepping RP, Mazur J (1997). Tracing the topology of the October 18–20, 1995, magnetic cloud with 0.1–$$10^2$$ keV electrons. Geophys Res Lett.

[CR173] Lavraud B, Ruffenach A, Rouillard AP, Kajdic P, Manchester WB, Lugaz N (2014). Geo-effectiveness and radial dependence of magnetic cloud erosion by magnetic reconnection. J Geophys Res.

[CR174] Lepping RP, Burlaga LF, Jones JA (1990). Magnetic field structure of interplanetary magnetic clouds at 1 AU. J Geophys Res.

[CR175] Lepping RP, Burlaga LF, Szabo A, Ogilvie KW, Mish WH, Vassiliadis D, Lazarus AJ, Steinberg JT, Farrugia CJ, Janoo L, Mariani F (1997). The Wind magnetic cloud and events of October 18–20, 1995: interplanetary properties and as triggers for geomagnetic activity. J Geophys Res.

[CR176] Lepping RP, Berdichevsky DB, Wu CC, Szabo A, Narock T, Mariani F, Lazarus AJ, Quivers AJ (2006). A summary of WIND magnetic clouds for years 1995–2003: model-fitted parameters, associated errors and classifications. Ann Geophys.

[CR177] Lepri ST, Zurbuchen TH (2010). Direct observational evidence of filament material within interplanetary coronal mass ejections. Astrophys J.

[CR178] Lepri ST, Zurbuchen TH, Fisk LA, Richardson IG, Cane HV, Gloeckler G (2001). Iron charge distribution as an identifier of interplanetary coronal mass ejections. J Geophys Res.

[CR179] Li X, Baker DN, Temerin M, Cayton T, Reeves GD, Araki T, Singer H, Larson D, Lin RP, Kanekal SG (1998). Energetic electron injections into the inner magnetosphere during the Jan. 10–11, 1997 magnetic storm. Geophys Res Lett.

[CR180] Li Y, Luhmann JG, Lynch BJ, Kilpua EKJ (2011). Cyclic reversal of magnetic cloud poloidal field. Sol Phys.

[CR181] Li Y, Luhmann JG, Lynch BJ, Kilpua EKJ (2014). Magnetic clouds and origins in STEREO era. J Geophys Res.

[CR182] Li X, Baker DN, Zhao H, Zhang K, Jaynes AN, Schiller Q, Kanekal SG, Blake JB, Temerin M (2017). Radiation belt electron dynamics at low l (4): Van Allen Probes era versus previous two solar cycles. J Geophys Res.

[CR183] Lilensten J, Coates AJ, Dehant V, Dudok de Wit T, Horne RB, Leblanc F, Luhmann J, Woodfield E, Barthélemy M (2014). What characterizes planetary space weather?. Astron Astrophys.

[CR184] Lindeman FA (1911). Note on the theory of magnetic storms. Philos Mag.

[CR185] Lindsay GM, Luhmann JG, Russell CT, Gosling JT (1999). Relationships between coronal mass ejection speeds from coronagraph images and interplanetary characteristics of associated interplanetary coronal mass ejections. J Geophys Res.

[CR186] Liu Y, Richardson JD, Belcher JW, Kasper JC, Skoug RM (2006). Plasma depletion and mirror waves ahead of interplanetary coronal mass ejections. J Geophys Res.

[CR187] Liu Y, Richardson JD, Belcher JW, Wang C, Hu Q, Kasper JC (2006). Constraints on the global structure of magnetic clouds: transverse size and curvature.. J Geophys Res.

[CR188] Liu Y, Luhmann JG, Huttunen KEJ, Lin RP, Bale SD, Russell CT, Galvin AB (2008) Reconstruction of the 2007 May 22 magnetic cloud: how much can we trust the flux-rope geometry of CMEs? Astrophys J Lett 677:L133. 10.1086/587839

[CR189] Liu YD, Luhmann JG, Kajdič P, Kilpua EKJ, Lugaz N, Nitta NV, Möstl C, Lavraud B, Bale SD, Farrugia CJ, Galvin AB (2014). Observations of an extreme storm in interplanetary space caused by successive coronal mass ejections. Nat Commun.

[CR190] Lockwood JA (1971). Forbush decreases in the cosmic radiation. Space Sci Rev.

[CR191] Lopez RE (1987). Solar cycle invariance in solar wind proton temperature relationships. J Geophys Res.

[CR192] Lugaz N, Manchester WB, Gombosi TI (2005). Numerical simulation of the interaction of two coronal mass ejections from Sun to Earth. Astrophys J.

[CR193] Lugaz N, Farrugia CJ, Manchester WB, Schwadron N (2013). The interaction of two coronal mass ejections: influence of relative orientation. Astrophys J.

[CR194] Lugaz N, Farrugia CJ, Smith CW, Paulson K (2015). Shocks inside CMEs: a survey of properties from 1997 to 2006. J Geophys Res.

[CR195] Lugaz N, Temmer M, Wang Y, Farrugia CJ (2017). The interaction of successive coronal mass ejections: a review. Sol Phys.

[CR196] Luhmann JG, Kasprzak WT, Russell CT (2007). Space weather at Venus and its potential consequences for atmosphere evolution. J Geophys Res.

[CR197] Lundquist S (1950). Magnetohydrostatic fields. Ark Fys.

[CR198] Lynch BJ, Zurbuchen TH, Fisk LA, Antiochos SK (2003). Internal structure of magnetic clouds: plasma and composition. J Geophys Res.

[CR199] Manchester WB, Gombosi TI, De Zeeuw DL, Sokolov IV, Roussev II, Powell KG, Kóta J, Tóth G, Zurbuchen TH (2005). Coronal mass ejection shock and sheath structures relevant to particle acceleration. Astrophys J.

[CR200] Manchester WB, Kozyra JU, Lepri ST, Lavraud B (2014). Simulation of magnetic cloud erosion during propagation. J Geophys Res.

[CR201] Manchester WB, Kilpua EKJ, Liu YD, Lugaz N, Riley P, Török T, Vršnak B (2017). The physical processes of CME/ICME evolution. Space Sci Rev.

[CR202] Mann IR, Ozeke LG, Murphy KR, Claudepierre SG, Turner DL, Baker DN, Rae IJ, Kale A, Milling DK, Boyd AJ, Spence HE, Reeves GD, Singer HJ, Dimitrakoudis S, Daglis IA, Honary F (2016). Explaining the dynamics of the ultra-relativistic third Van Allen radiation belt. Nature Phys.

[CR203] Marubashi K, Lepping RP (2007). Long-duration magnetic clouds: a comparison of analyses using torus- and cylinder-shaped flux rope models. Ann Geophys.

[CR204] Mason GM (2007). $$^{3}$$He-rich solar energetic particle events. Space Sci Rev.

[CR205] Mason GM, Nitta NV, Wiedenbeck ME, Innes DE (2016). Evidence for a common acceleration mechanism for enrichments of $$^{3}$$He and heavy ions in impulsive SEP events. Astrophys J.

[CR206] Mayaud PN (1980). Derivation, meaning, and use of geomagnetic indices. Geophysical Monograph.

[CR207] McComas DJ, Gosling JT, Winterhalter D, Smith EJ (1988). Interplanetary magnetic field draping about fast coronal mass ejecta in the outer heliosphere. J Geophys Res.

[CR208] McComas DJ, Goldstein R, Gosling JT, Skoug RM (2001). Ulysses’ second orbit: remarkably different solar wind. Space Sci Rev.

[CR209] McComas DJ, Elliott HA, Schwadron NA, Gosling JT, Skoug RM, Goldstein BE (2003). The three-dimensional solar wind around solar maximum. Geophys Res Lett.

[CR210] Mironova IA, Aplin KL, Arnold F, Bazilevskaya GA, Harrison RG, Krivolutsky AA, Nicoll KA, Rozanov EV, Turunen E, Usoskin IG (2015). Energetic particle influence on the Earth’s atmosphere. Space Sci Rev.

[CR211] Morrison P (1956). Solar origin of cosmic-ray time variations. Phys Rev.

[CR212] Möstl C, Farrugia CJ, Miklenic C, Temmer M, Galvin AB, Luhmann JG, Kilpua EKJ, Leitner M, Nieves-Chinchilla T, Veronig A, Biernat HK (2009). Multispacecraft recovery of a magnetic cloud and its origin from magnetic reconnection on the Sun. J Geophys Res.

[CR213] Möstl C, Farrugia CJ, Kilpua EKJ, Jian LK, Liu Y, Eastwood JP, Harrison RA, Webb DF, Temmer M, Odstrčil D, Davies JA, Rollett T, Luhmann JG, Nitta N, Mulligan T, Jensen EA, Forsyth R, Lavraud B, de Koning CA, Veronig AM, Galvin AB, Zhang TL, Anderson BJ (2012). Multi-point shock and flux rope analysis of multiple interplanetary coronal mass ejections around 2010 August 1 in the inner heliosphere. Astrophys J.

[CR214] Mulligan T, Russell CT (2001). Multispacecraft modeling of the flux rope structure of interplanetary coronal mass ejections: cylindrically symmetric versus nonsymmetric topologies. J Geophys Res.

[CR215] Mulligan T, Russell CT, Luhmann JG (1998). Solar cycle evolution of the structure of magnetic clouds in the inner heliosphere. Geophys Res Lett.

[CR216] Mulligan T, Russell CT, Anderson BJ, Lohr DA, Rust D, Toth BA, Zanetti LJ, Acuna MH, Lepping RP, Gosling JT (1999). Intercomparison of NEAR and Wind interplanetary coronal mass ejection observations. J Geophys Res.

[CR217] Munro RH, Gosling JT, Hildner E, MacQueen RM, Poland AI, Ross CL (1979). The association of coronal mass ejection transients with other forms of solar activity. Sol Phys.

[CR218] Mursula K, Lukianova R, Holappa L (2015). Occurrence of high-speed solar wind streams over the grand Modern Maximum. Astrophys J.

[CR219] Myllys M, Kilpua EKJ, Lavraud B, Pulkkinen TI (2016). Solar wind-magnetosphere coupling efficiency during ejecta and sheath-driven geomagnetic storms. J Geophys Res.

[CR220] Nakagawa T, Nishida A, Saito T (1989). Planar magnetic structures in the solar wind. J Geophys Res.

[CR221] Neugebauer M, Clay DR, Gosling JT (1993). The origins of planar magnetic structures in the solar wind. J Geophys Res.

[CR222] Nieves-Chinchilla T, Viñas AF (2008). Solar wind electron distribution functions inside magnetic clouds. J Geophys Res.

[CR223] Odstrčil D, Pizzo VJ (1999). Three-dimensional propagation of CMEs in a structured solar wind flow: 1. CME launched within the streamer belt. J Geophys Res.

[CR224] Odstrčil D, Pizzo VJ (1999). Three-dimensional propagation of coronal mass ejections in a structured solar wind flow 2. CME launched adjacent to the streamer belt. J Geophys Res.

[CR225] Osherovich VA, Farrugia CJ, Burlaga LF (1993). Dynamics of aging magnetic clouds. Adv Space Res.

[CR226] Osherovich VA, Farrugia CJ, Burlaga LF (1995). Nonlinear evolution of magnetic flux ropes. 2: finite beta plasma. J Geophys Res.

[CR227] Owens MJ (2016). Do the legs of magnetic clouds contain twisted flux-rope magnetic fields?. Astrophys J.

[CR228] Owens MJ, Cargill PJ, Pagel C, Siscoe GL, Crooker NU (2005). Characteristic magnetic field and speed properties of interplanetary coronal mass ejections and their sheath regions. J Geophys Res.

[CR229] Palmer ID, Allum FR, Singer S (1978). Bidirectional anisotropies in solar cosmic ray events—evidence for magnetic bottles. J Geophys Res.

[CR230] Palmerio E, Kilpua EKJ, Savani NP (2016). Planar magnetic structures in coronal mass ejection-driven sheath regions. Ann Geophys.

[CR231] Palmroth M, Partamies N, Polvi J, Pulkkinen TI, McComas DJ, Barnes RJ, Stauning P, Smith CW, Singer HJ, Vainio R (2007). Solar wind–magnetosphere coupling efficiency for solar wind pressure impulses. Geophys Res Lett.

[CR232] Paulikas GA, Blake JB (1979) Effects of the solar wind on magnetospheric dynamics: energetic electrons at the synchronous orbit. In: Olson WP (ed) Quantitative modeling of magnetospheric processes. Geophysical Monograph, vol 21, American Geophysical Union, Washington, DC, pp 180–202. 10.1029/GM021p0180

[CR233] Petrosian V, Jiang YW, Liu S, Ho GC, Mason GM (2009). Relative distributions of fluences of $$^{3}$$He and $$^{4}$$He in solar energetic particles. Astrophys J.

[CR234] Phan TD, Paschmann G, Gosling JT, Oieroset M, Fujimoto M, Drake JF, Angelopoulos V (2013). The dependence of magnetic reconnection on plasma $$\beta $$ and magnetic shear: evidence from magnetopause observations. Geophys Res Lett.

[CR235] Piddington JH (1958). Interplanetary magnetic field and its control of cosmic-ray variations. Phys Rev.

[CR236] Plainaki C, Lilensten J, Radioti A, Andriopoulou M, Milillo A, Nordheim TA, Dandouras I, Coustenis A, Grassi D, Mangano V, Massetti S, Orsini S, Lucchetti A (2016). Planetary space weather: scientific aspects and future perspectives. J Space Weather Space Clim.

[CR237] Prikryl P, Jayachandran PT, Mushini SC, Richardson IG (2014). High-latitude GPS phase scintillation and cycle slips during high-speed solar wind streams and interplanetary coronal mass ejections: a superposed epoch analysis. Earth, Planets and Space.

[CR238] Pulkkinen T (2007) Space weather: terrestrial perspective. Living Rev Sol Phys 4:lrsp-2007-1. 10.12942/lrsp-2007-1

[CR239] Pulkkinen A (2015). Geomagnetically induced currents modeling and forecasting. Space Weather.

[CR240] Qiu J, Hu Q, Howard TA, Yurchyshyn VB (2007). On the magnetic flux budget in low-corona magnetic reconnection and interplanetary coronal mass ejections. Astrophys J.

[CR241] Reames DV (1999). Particle acceleration at the Sun and in the heliosphere. Space Sci Rev.

[CR242] Reames DV (2015). What are the sources of solar energetic particles? Element abundances and source plasma temperatures. Space Sci Rev.

[CR243] Rees A, Forsyth RJ (2004). Two examples of magnetic clouds with double rotations observed by the Ulysses spacecraft. Geophys Res Lett.

[CR244] Reeves GD, McAdams KL, Friedel RHW, O’Brien TP (2003). Acceleration and loss of relativistic electrons during geomagnetic storms. Geophys Res Lett.

[CR245] Reeves GD, Morley SK, Friedel RHW, Henderson MG, Cayton TE, Cunningham G, Blake JB, Christensen RA, Thomsen D (2011). On the relationship between relativistic electron flux and solar wind velocity: Paulikas and Blake revisited. J Geophys Res.

[CR246] Reeves GD, Spence HE, Henderson MG, Morley SK, Friedel RHW, Funsten HO, Baker DN, Kanekal SG, Blake JB, Fennell JF, Claudepierre SG, Thorne RM, Turner DL, Kletzing CA, Kurth WS, Larsen BA, Niehof JT (2013). Electron Acceleration in the heart of the Van Allen radiation belts. Science.

[CR247] Reisenfeld DB, Gosling JT, Forsyth RJ, Riley P, St Cyr OC (2003). Properties of high-latitude CME-driven disturbances during Ulysses second northern polar passage. Geophys Res Lett.

[CR248] Richardson IG, Crooker N, Joselyn JA, Feynman J (1997). Using energetic particles to probe the magnetic topology of ejecta. Coronal mass ejections. Geophysical Monograph.

[CR249] Richardson JD (2011). Shocks and sheaths in the heliosphere. J Atmos Terr Phys.

[CR250] Richardson IG (2013). Geomagnetic activity during the rising phase of solar cycle 24. J Space Weather Space Clim.

[CR251] Richardson IG (2014). Identification of interplanetary coronal mass ejections at Ulysses using multiple solar wind signatures. Sol Phys.

[CR252] Richardson IG, Cane HV (1993). Signatures of shock drivers in the solar wind and their dependence on the solar source location. J Geophys Res.

[CR253] Richardson IG, Cane HV (1995). Regions of abnormally low proton temperature in the solar wind (1965–1991) and their association with ejecta. J Geophys Res.

[CR254] Richardson IG, Cane HV (2004). Identification of interplanetary coronal mass ejections at 1 AU using multiple solar wind plasma composition anomalies. J Geophys Res.

[CR255] Richardson IG, Cane HV (2010). Near-Earth interplanetary coronal mass ejections during solar cycle 23 (1996–2009): catalog and summary of properties. Sol Phys.

[CR256] Richardson IG, Cane HV (2011). Galactic cosmic ray intensity response to interplanetary coronal mass ejections/magnetic clouds in 1995–2009. Sol Phys.

[CR257] Richardson IG, Cane HV (2012). Near-earth solar wind flows and related geomagnetic activity during more than four solar cycles (1963–2011). J Space Weather Space Clim.

[CR258] Richardson IG, Cane HV (2012). Solar wind drivers of geomagnetic storms during more than four solar cycles. J Space Weather Space Clim.

[CR259] Richardson JD, Paularena KI, Wang C, Burlaga LF (2002). The life of a CME and the development of a MIR: from the Sun to 58 AU. J Geophys Res.

[CR260] Richardson JD, Liu Y, Wang C, Burlaga LF (2006). ICMEs at very large distances. Adv Space Res.

[CR261] Richardson IG, von Rosenvinge TT, Cane HV, Christian ER, Cohen CMS, Labrador AW, Leske RA, Mewaldt RA, Wiedenbeck ME, Stone EC (2014). $$> 25$$ MeV proton events observed by the high energy telescopes on the STEREO A and B spacecraft and/or at Earth during the first $$\sim $$ seven years of the STEREO mission. Solar Phys.

[CR262] Riley P, Crooker NU (2004). Kinematic treatment of coronal mass ejection evolution in the solar wind. Astrophys J.

[CR263] Riley P, Richardson IG (2013). Using statistical multivariable models to understand the relationship between interplanetary coronal mass ejecta and magnetic flux ropes. Sol Phys.

[CR264] Riley P, Gosling JT, Pizzo VJ (2001). Investigation of the polytropic relationship between density and temperature within interplanetary coronal mass ejections using numerical simulations. J Geophys Res.

[CR265] Riley P, Linker JA, Lionello R, Mikić Z, Odstrčil D, Hidalgo MA, Cid C, Hu Q, Lepping RP, Lynch BJ, Rees A (2004). Fitting flux ropes to a global MHD solution: a comparison of techniques. J Atmos Terr Phys.

[CR266] Riley P, Schatzman C, Cane HV, Richardson IG, Gopalswamy N (2006). On the rates of coronal mass ejections: remote solar and in situ observations. Astrophys J.

[CR267] Riley P, Caplan RM, Giacalone J, Lario D, Liu Y (2016). Properties of the fast forward shock driven by the July 23 2012 extreme coronal mass ejection. Astrophys J.

[CR268] Rivera EJ, Lissauer JJ, Butler RP, Marcy GW, Vogt SS, Fischer DA, Brown TM, Laughlin G, Henry GW (2005). A $$\sim 7.5 M_E$$ Planet orbiting the nearby Star, GJ 876. Astrophys J.

[CR269] Rodriguez L, Masías-Meza JJ, Dasso S, Démoulin P, Zhukov AN, Gulisano AM, Mierla M, Kilpua E, West M, Lacatus D, Paraschiv A, Janvier M (2016). Typical profiles and distributions of plasma and magnetic field parameters in magnetic clouds at 1 AU. Sol Phys.

[CR270] Romashets EP, Vandas M (2003). Force-free field inside a toroidal magnetic cloud. Geophys Res Lett.

[CR271] Rouillard AP (2011). Relating white light and in situ observations of coronal mass ejections: a review. J Atmos Solar Terr Phys.

[CR272] Rouillard AP, Sheeley NR, Tylka A, Vourlidas A, Ng CK, Rakowski C, Cohen CMS, Mewaldt RA, Mason GM, Reames D, Savani NP, StCyr OC, Szabo A (2012). The longitudinal properties of a solar energetic particle event investigated using modern solar imaging. Astrophys J.

[CR273] Ruffenach A, Lavraud B, Owens MJ, Sauvaud JA, Savani NP, Rouillard AP, Démoulin P, Foullon C, Opitz A, Fedorov A, Jacquey CJ, Génot V, Louarn P, Luhmann JG, Russell CT, Farrugia CJ, Galvin AB (2012). Multispacecraft observation of magnetic cloud erosion by magnetic reconnection during propagation. J Geophys Res.

[CR274] Ruffenach A, Lavraud B, Farrugia CJ, Démoulin P, Dasso S, Owens MJ, Sauvaud JA, Rouillard AP, Lynnyk A, Foullon C, Savani NP, Luhmann JG, Galvin AB (2015). Statistical study of magnetic cloud erosion by magnetic reconnection. J Geophys Res.

[CR275] Russell CT, Mulligan T (2002). On the magnetosheath thicknesses of interplanetary coronal mass ejections. Planet Space Sci.

[CR276] Russell CT, Shinde AA, Jian L (2005). A new parameter to define interplanetary coronal mass ejections. Adv Space Res.

[CR277] Russell CT, Mewaldt RA, Luhmann JG, Mason GM, von Rosenvinge TT, Cohen CMS, Leske RA, Gomez-Herrero R, Klassen A, Galvin AB, Simunac KDC (2013). The very unusual interplanetary coronal mass ejection of 2012 July 23: a blast wave mediated by solar energetic particles. Astrophys J.

[CR278] Schmidt JM, Cargill PJ (2003). Magnetic reconnection between a magnetic cloud and the solar wind magnetic field. J Geophys Res.

[CR279] Schrijver CJ, Kauristie K, Aylward AD, Denardini CM, Gibson SE, Glover A, Gopalswamy N, Grande M, Hapgood M, Heynderickx D, Jakowski N, Kalegaev VV, Lapenta G, Linker JA, Liu S, Mandrini CH, Mann IR, Nagatsuma T, Nandy D, Obara T, Paul O’Brien T, Onsager T, Opgenoorth HJ, Terkildsen M, Valladares CE, Vilmer N (2015). Understanding space weather to shield society: a global road map for 2015–2025 commissioned by COSPAR and ILWS. Adv Space Res.

[CR280] Schwenn R (1983). Direct correlations between coronal transients and interplanetary disturbances. Space Sci Rev.

[CR281] Schwenn R (2006) Space weather: the solar perspective. Living Rev Sol Phys 3:2. 10.12942/lrsp-2006-2. http://www.livingreviews.org/lrsp-2006-2

[CR282] Schwenn R, dal Lago A, Huttunen E, Gonzalez WD (2005). The association of coronal mass ejections with their effects near the Earth. Ann Geophys.

[CR283] Scurry L, Russell CT, Gosling JT (1994). Geomagnetic activity and the beta dependence of the dayside reconnection rate. J Geophys Res.

[CR284] Seppälä A, Matthes K, Randall CE, Mironova IA (2014). What is the solar influence on climate? Overview of activities during CAWSES-II. Progr Earth Planet Sci.

[CR285] Sheeley NR, Howard RA, Michels DJ, Koomen MJ, Schwenn R, Muehlhaeuser KH, Rosenbauer H (1985). Coronal mass ejections and interplanetary shocks. J Geophys Res.

[CR286] Sheeley NR, Walters JH, Wang YM, Howard RA (1999). Continuous tracking of coronal outflows: two kinds of coronal mass ejections. J Geophys Res.

[CR287] Shimazu H, Vandas M (2002). A self-similar solution of expanding cylindrical flux ropes for any polytropic index value. Earth Planets Space.

[CR288] Shodhan S, Crooker NU, Kahler SW, Fitzenreiter RJ, Larson DE, Lepping RP, Siscoe GL, Gosling JT (2000). Counterstreaming electrons in magnetic clouds. J Geophys Res.

[CR289] Siscoe G, Odstrčil D (2008). Ways in which ICME sheaths differ from magnetosheaths. J Geophys Res.

[CR290] Siscoe G, MacNeice PJ, Odstrčil D (2007). East-west asymmetry in coronal mass ejection geoeffectiveness. Space Weather.

[CR291] Steinberg JT, Gosling JT, Skoug RM, Wiens RC (2005). Suprathermal electrons in high-speed streams from coronal holes: counterstreaming on open field lines at 1 AU. J Geophys Res.

[CR292] Summers D, Omura Y, Nakamura S, Kletzing CA (2014). Fine structure of plasmaspheric hiss. J Geophys Res.

[CR293] Taylor JB (1986). Relaxation and magnetic reconnection in plasmas. Rev Mod Phys.

[CR294] Temmer M (2017). On flare-CME characteristics from Sun to Earth combining remote-sensing image data with in-situ measurements supported by modeling. Solar Phys.

[CR295] Thorne RM, Li W, Ni B, Ma Q, Bortnik J, Chen L, Baker DN, Spence HE, Reeves GD, Henderson MG, Kletzing CA, Kurth WS, Hospodarsky GB, Blake JB, Fennell JF, Claudepierre SG, Kanekal SG (2013). Rapid local acceleration of relativistic radiation-belt electrons by magnetospheric chorus. Nature.

[CR296] Török T, Kliem B (2005). Confined and ejective eruptions of kink-unstable flux ropes. Astrophys J Lett.

[CR297] Tousey R, Rycroft MJ, Runcorn SK (1973). The solar corona. Space research XIII.

[CR298] Trottet G, Samwel S, Klein KL, Dudok de Wit T, Miteva R (2015). Statistical evidence for contributions of flares and coronal mass ejections to major solar energetic particle events. Solar Phys.

[CR299] Tsurutani BT, Smith EJ, Gonzalez WD, Tang F, Akasofu SI (1988). Origin of interplanetary southward magnetic fields responsible for major magnetic storms near solar maximum (1978–1979). J Geophys Res.

[CR300] Vainio R, Pönni A, Battarbee M, Afanasiev A, Koskinen HEJ, Laitinen T (2014). A semi-analytical foreshock model for energetic storm particle events inside 1 AU. J Space Weather Space Clim.

[CR301] van Driel-Gesztelyi L, Démoulin P, Mandrini CH (2003). Observations of magnetic helicity. Adv Space Res.

[CR302] Vandas M, Romashets EP (2003). A force-free field with constant alpha in an oblate cylinder: a generalization of the Lundquist solution. Astron Astrophys.

[CR303] Vandas M, Fischer S, Pelant P, Geranios A (1993). Spheroidal models of magnetic clouds and their comparison with spacecraft measurements. J Geophys Res.

[CR304] Vandas M, Romashets EP, Watari S, Geranios A, Antoniadou E, Zacharopoulou O (2006). Comparison of force-free flux rope models with observations of magnetic clouds. Adv Space Res.

[CR305] Vandas M, Romashets E, Geranios A (2015). Modeling of magnetic cloud expansion. Astron Astrophys.

[CR306] Verkhoglyadova OP, Zank GP, Li G (2015). A theoretical perspective on particle acceleration by interplanetary shocks and the Solar Energetic Particle problem. Phys Rep.

[CR307] Verronen PT, Rodger CJ, Clilverd MA, Wang S (2011). First evidence of mesospheric hydroxyl response to electron precipitation from the radiation belts. J Geophys Res.

[CR308] Vida K, Kriskovics L, Oláh K, Leitzinger M, Odert P, Kővári Z, Korhonen H, Greimel R, Robb R, Csák B, Kovács J (2016). Investigating magnetic activity in very stable stellar magnetic fields. Long-term photometric and spectroscopic study of the fully convective M4 dwarf V374 Pegasi. Astron Astrophys.

[CR309] von Steiger R, Richardson JD (2006). ICMEs in the outer heliosphere and at high latitudes: an introduction. Space Sci Rev.

[CR310] Vourlidas A, Lynch BJ, Howard RA, Li Y (2013). How many cmes have flux ropes? Deciphering the signatures of shocks, flux ropes, and prominences in coronagraph observations of cmes. Sol Phys.

[CR311] Wang Y, Zhuang B, Hu Q, Liu R, Shen C, Chi Y (2016). On the twists of interplanetary magnetic flux ropes observed at 1 AU. J Geophys Res.

[CR312] Webb DF, Howard TA (2012) Coronal mass ejections: observations. Living Rev Sol Phys 9:3. 10.12942/lrsp-2012-3. http://www.livingreviews.org/lrsp-2012-3

[CR313] Webb DF, Cliver EW, Crooker NU, Cry OCS, Thompson BJ (2000). Relationship of halo coronal mass ejections, magnetic clouds, and magnetic storms. J Geophys Res.

[CR314] Wei F, Liu R, Fan Q, Feng X (2003). Identification of the magnetic cloud boundary layers. J Geophys Res.

[CR315] Wei F, Liu R, Feng X, Zhong D, Yang F (2003). Magnetic structures inside boundary layers of magnetic clouds. Geophys Res Lett.

[CR316] Wibberenz G, Le Roux JA, Potgieter MS, Bieber JW (1998). Transient effects and disturbed conditions. Space Sci Rev.

[CR317] Wild JP, Smerd SF, Weiss AA (1963). Solar bursts. Ann Rev Astron Astrophys.

[CR318] Wilson RM, Hildner E (1984). Are interplanetary magnetic clouds manifestations of coronal transients at 1 AU?. Sol Phys.

[CR319] Wimmer-Schweingruber RF, Crooker NU, Balogh A, Bothmer V, Forsyth RJ, Gazis P, Gosling JT, Horbury T, Kilchenmann A, Richardson IG, Richardson JD, Riley P, Rodriguez L, Steiger RV, Wurz P, Zurbuchen TH (2006). Understanding interplanetary coronal mass ejection signatures. Report of Working Group B. Space Sci Rev.

[CR320] Witasse O, Sánchez-Cano B, Mays ML, Kajdič P, Opgenoorth H, Elliott HA, Richardson IG, Zouganelis I, Zender J, Wimmer-Schweingruber RF, Turc L, Taylor MGGT, Roussos E, Rouillard A, Richter I, Richardson JD, Ramstad R, Provan G, Posner A, Plaut JJ, Odstrčil D, Nilsson H, Niemenen P, Milan SE, Mandt K, Lohf H, Lester M, Lebreton JP, Kuulkers E, Krupp N, Koenders C, James MK, Intzekara D, Holmstrom M, Hassler DM, Hall BES, Guo J, Goldstein R, Goetz C, Glassmeier KH, Gnot V, Evans H, Espley J, Edberg NJT, Dougherty M, Cowley SWH, Burch J, Behar E, Barabash S, Andrews DJ, Altobelli N (2017) Interplanetary coronal mass ejection observed at STEREO-A, Mars, comet 67P/Churyumov-Gerasimenko, Saturn, and New Horizons en route to Pluto: comparison of its Forbush decreases at 1.4, 3.1, and 9.9 AU. J Geophys Res. 10.1002/2017JA023884

[CR321] Woltjer L (1958). A theorem on force-free magnetic fields. Proc Natl Acad Sci USA.

[CR322] Wood BE, Müller HR, Zank GP, Linsky JL, Redfield S (2005). New mass-loss measurements from astrospheric Ly$$\alpha $$ absorption. Astrophys J Lett.

[CR323] Wood BE, Howard RA, Linton MG (2016). Imaging prominence eruptions out to 1 AU. Astrophys J.

[CR324] Wu CC, Lepping RP (2011). Statistical comparison of magnetic clouds with interplanetary coronal mass ejections for solar cycle 23. Sol Phys.

[CR325] Wu CC, Lepping RP (2016). Relationships among geomagnetic storms, interplanetary shocks, magnetic clouds, and sunspot number during 1995–2012. Sol Phys.

[CR326] Zank GP, Hunana P, Mostafavi P, Le Roux JA, Li G, Webb GM, Khabarova O, Cummings A, Stone E, Decker R (2015). Diffusive shock acceleration and reconnection acceleration processes. Astrophys J.

[CR327] Zhang J, Liemohn MW, Kozyra JU, Lynch BJ, Zurbuchen TH (2004). A statistical study of the geoeffectiveness of magnetic clouds during high solar activity years. J Geophys Res.

[CR328] Zhang J, Richardson IG, Webb DF, Gopalswamy N, Huttunen E, Kasper JC, Nitta NV, Poomvises W, Thompson BJ, Wu CC, Yashiro S, Zhukov AN (2007). Solar and interplanetary sources of major geomagnetic storms ($$Dst\le -100$$ nT) during 1996–2005. J Geophys Res.

[CR329] Zhao L, Zurbuchen TH, Fisk LA (2009). Global distribution of the solar wind during solar cycle 23: ACE observations. Geophys Res Lett.

[CR330] Zurbuchen TH, Richardson IG (2006). In-situ solar wind and magnetic field signatures of interplanetary coronal mass ejections. Space Sci Rev.

[CR331] Zurbuchen TH, Weberg M, von Steiger R, Mewaldt RA, Lepri ST, Antiochos SK (2016). Composition of coronal mass ejections. Astrophys J.

[CR332] Zwan BJ, Wolf RA (1976). Depletion of solar wind plasma near a planetary boundary. J Geophys Res.

